# A Comprehensive, Valid, and Reliable Tool to Assess the Degree of Responsibility of Digital Health Solutions That Operate With or Without Artificial Intelligence: 3-Phase Mixed Methods Study

**DOI:** 10.2196/48496

**Published:** 2023-08-28

**Authors:** Pascale Lehoux, Robson Rocha de Oliveira, Lysanne Rivard, Hudson Pacifico Silva, Hassane Alami, Carl Maria Mörch, Kathy Malas

**Affiliations:** 1 Department of Health Management, Evaluation and Policy, Université de Montréal; Center for Public Health Research Montréal, QC Canada; 2 Center for Public Health Research, Université de Montréal Montréal, QC Canada; 3 Interdisciplinary Research in Health Sciences, Nuffield Department of Primary Care Health Sciences, University of Oxford Oxford United Kingdom; 4 AI for the Common Good Institute Université Libre de Bruxelles Bruxelles Belgium; 5 Innovation and Artificial Intelligence, Executive Office Centre hospitalier de l’Université de Montréal Montréal, QC Canada

**Keywords:** Responsible Innovation in Health, digital health policy, artificial intelligence ethics, responsible research and innovation, mixed methods, e-Delphi, interrater agreement, mobile phone

## Abstract

**Background:**

Clinicians’ scope of responsibilities is being steadily transformed by digital health solutions that operate with or without artificial intelligence (DAI solutions). Most tools developed to foster ethical practices lack rigor and do not concurrently capture the health, social, economic, and environmental issues that such solutions raise.

**Objective:**

To support clinical leadership in this field, we aimed to develop a comprehensive, valid, and reliable tool that measures the responsibility of DAI solutions by adapting the multidimensional and already validated Responsible Innovation in Health Tool.

**Methods:**

We conducted a 3-phase mixed methods study. Relying on a scoping review of available tools, phase 1 (concept mapping) led to a preliminary version of the Responsible DAI solutions Assessment Tool. In phase 2, an international 2-round e-Delphi expert panel rated on a 5-level scale the importance, clarity, and appropriateness of the tool’s components. In phase 3, a total of 2 raters independently applied the revised tool to a sample of DAI solutions (n=25), interrater reliability was measured, and final minor changes were made to the tool.

**Results:**

The mapping process identified a comprehensive set of responsibility premises, screening criteria, and assessment attributes specific to DAI solutions. e-Delphi experts critically assessed these new components and provided comments to increase content validity (n=293), and after round 2, consensus was reached on 85% (22/26) of the items surveyed. Interrater agreement was *substantial* for a subcriterion and *almost perfect* for all other criteria and assessment attributes.

**Conclusions:**

The Responsible DAI solutions Assessment Tool offers a comprehensive, valid, and reliable means of assessing the degree of responsibility of DAI solutions in health. As regulation remains limited, this forward-looking tool has the potential to change practice toward more equitable as well as economically and environmentally sustainable digital health care.

## Introduction

### Background

Over the past decade, digital health solutions and those relying on artificial intelligence (AI) have exponentially grown and expanded research and health care practices in ways that were previously unthinkable [1]. As AI is entirely dependent on digital infrastructures [2], the inclusive term “digital health solutions that operate with or without AI” (“DAI solutions”) is used throughout this paper to refer to electronic systems that rely on software and possibly also hardware to generate, store, or process data and that operate with or without AI [3]. Although DAI solutions are steadily transforming health systems [4] as well as clinicians’ practices and scope of responsibilities [5], health care providers involved in the development and assessment of these tools mainly focus their attention on safety, effectiveness, and biases [6]. However, as the DAI solutions industry within which the digital health field evolves is not guided by a professional care ethos, strong clinical leadership is required for DAI solutions in health to remain aligned not only with patients’ needs and health care values and goals [4] but also with current knowledge on the effects of climate change on health [7,8]. Research shows that DAI solutions in health that are not properly designed or implemented increase digital health inequalities [9,10] and that their use requires more devices and data infrastructures that cause environmental harms (eg, material mining, e-waste disposal, and energy use) [11,12]. As “a relentless drive” to use larger amounts of data and more sophisticated computational capacities comes with higher environmental costs, the powerful clinical tools that DAI solutions offer thus entail substantial “trade-offs” that clinicians can no longer ignore [7].

As health care providers and health systems worldwide will be on the front line tackling the health effects of climate change and growing social and economic disparities [10,11], clinical leaders (eg, physicians, nurses, occupational therapists, and psychologists) will have to play a much broader role in the design and assessment of DAI solutions. They must be able to anticipate and properly prepare trainees and practitioners to address the health, social, economic, and environmental impacts of the DAI solutions they work with or recommend to patients or that their organizations acquire. As these multidimensional impacts are linked to each other, clinical leaders urgently require tools so they can comprehensively and efficiently assess the relevance of DAI solutions “prior to implementation” and “lead the change” needed in partnership with other health care stakeholders for such solutions to support patient care and health systems in a meaningful and responsible way [4].

### Research Gaps

Many ethical principles (eg, privacy, accountability, and robustness) have been proposed to foster responsibility in the digital industry, either specifically for health care [13] or for multiple sectors [14] and either for digital solutions [15] or for AI [16]. The scoping review our team recently completed highlights key gaps in the practice-oriented tools developed since 2015 [17]. First, these tools are highly heterogeneous, which may facilitate “mixing and matching” [18] principles that do not rely on a solidly defined conceptual framework. For instance, among the 56 tools we identified, ≥50% (≥10/19) of those from the health sector relied on a small number of principles (n=10), ≥50% (≥20/37) of the multisector tools covered twice as many principles (n=19), and most tools (≥29/56, ≥50%) disregarded 21 principles over a total of 40 principles found in the 56 tools. Second, the methodology used to develop 82% of the tools was not defined, 18% used engagement methods (eg, workshops and consultations), and none reported how quality was assessed. This is a major research gap as tools that lack a solid methodology may undermine at its roots the very goal of fostering responsible DAI solutions: clinical leaders are unlikely to adopt them “if their quality or credibility is perceived as low” [17].

### Goal of the Study and Approach

To support clinical leadership in this rapidly evolving field, the goal of our study was to develop a comprehensive, valid, and reliable tool to measure the degree of responsibility of DAI solutions in health. The Responsible Innovation in Health (RIH) framework [19], which brings together key health, social, economic, and environmental issues, offered a solid basis to develop such a tool as RIH is anchored in an evidence-informed health research tradition [20]. Its accompanying RIH assessment tool is one of the rare tools in the field of responsible research and innovation that is specific to the health care sector [21] and that provides a conceptually valid [22] and reliable [23] quantitative measure of responsibility. However, the RIH tool does not capture responsibility issues specific to DAI solutions (eg, data management).

Following Stilgoe et al [24], for whom responsible innovation means “taking care of the future through collective stewardship of science and innovation in the present,” RIH is forward-looking, pragmatic, and multidisciplinary [25]. RIH goes beyond deontology and bioethics as it aims to steer health innovation toward equitable as well as economically and environmentally sustainable health systems [26]. The RIH framework approaches responsibility as a matter of degree, which can be appraised by examining 9 responsibility attributes falling within five value domains: (1) population health value (health relevance; health inequalities; and ethical, legal, and social issues), (2) health system value (responsiveness, inclusiveness, and level and intensity of care), (3) economic value (frugality), (4) organizational value (business model), and (5) environmental value (eco-responsibility). Although the first 2 value domains are familiar to clinicians, the other 3 offer key considerations for taking care of the future when developing health innovations [26]. Through its “Frugality” attribute, RIH underscores that an innovation adds economic value when it is designed to be affordable and easy to use and optimized for its context of use without neglecting low-resource settings [27]. The “Business model” attribute emphasizes organizations that are stakeholder centered (ie, that create value for society, not only for shareholders [28,29]). Finally, the “Eco-responsibility” attribute recognizes that planetary health and human health are deeply intertwined [11].

**Figure 1:**
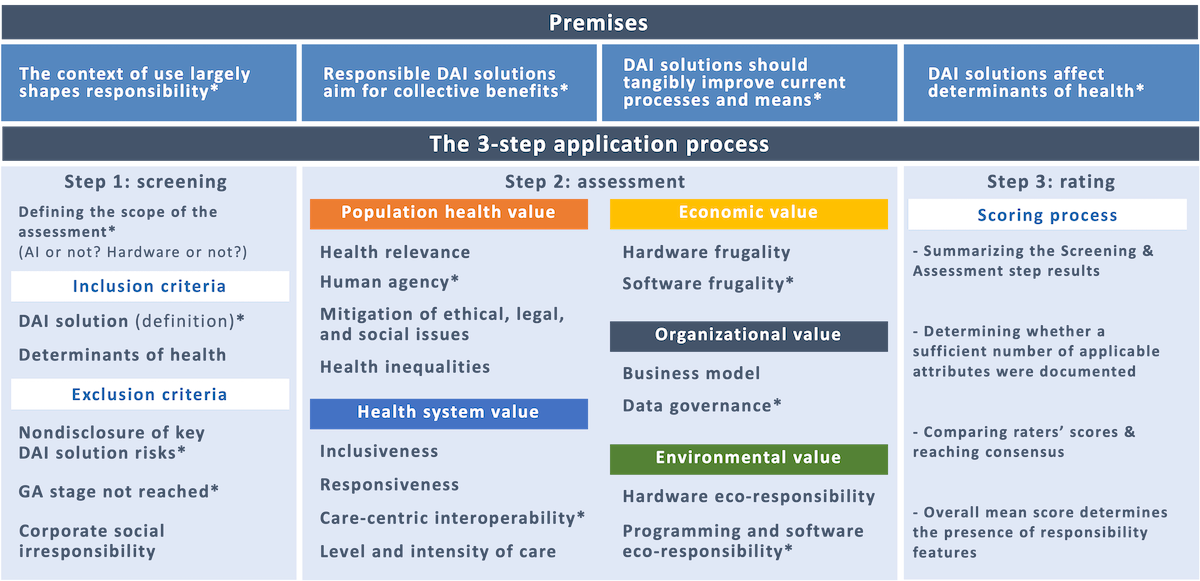
Overview of the key components of the Responsible DAI solutions Assessment Tool. *New components integrated to the original RIH Assessment Tool. AI: artificial intelligence; DAI solutions: digital health solutions that operate with or without artificial intelligence; GA: general availability.

In this study, the iterative research process that led to the RIH tool [22,23] was replicated to (1) adapt the RIH tool to the specificities of DAI solutions, (2) validate the constructs of the resulting Responsible DAI solutions Assessment Tool (hereafter referred to as the “tool”), and (3) assess its reliability. To facilitate readers’ understanding, Figure 1 summarizes the tool’s key components: 4 premises, 5 screening criteria, and 14 assessment attributes. The type of information its 3-step application process (screening, assessment, and scoring) requires is described in the tool, which can be found in Multimedia Appendix 1. The attributes use a 4-level scale ranging from A to D, where A implies a “high degree of responsibility” and D implies “no particular signs of responsibility.” As the attributes do not measure “irresponsibility,” the screening criteria constitute baseline responsibility requirements (eg, efficacy, safety, and privacy) that serve as a “stopping rule” in the assessment process.

## Methods

### Study Design

**Figure 2:**
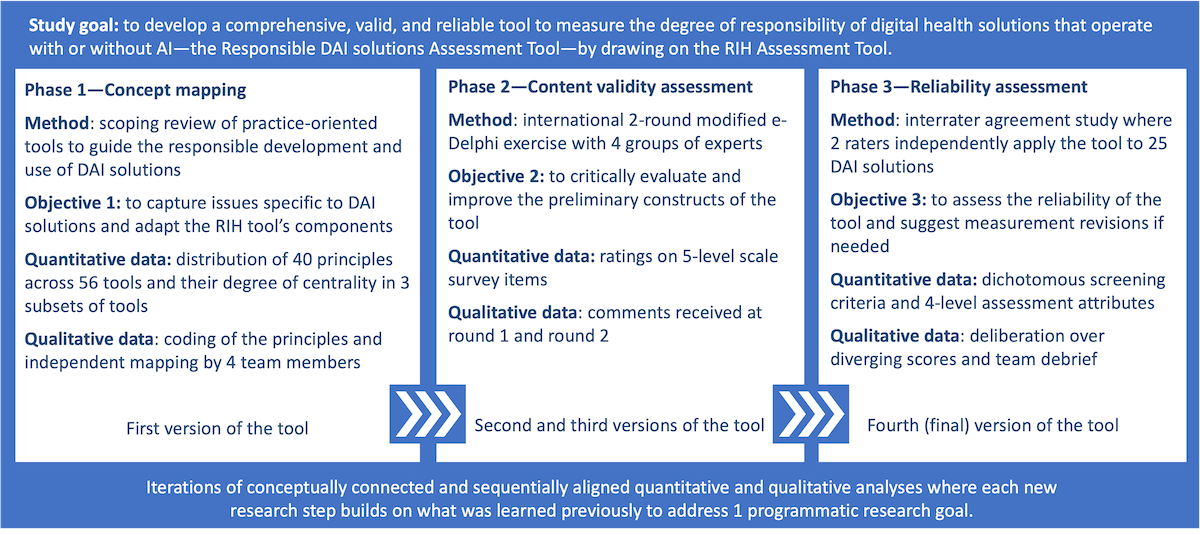
Mixed methods study design. AI: artificial intelligence; DAI solutions: digital health solutions that operate with or without artificial intelligence; RIH: Responsible Innovation in Health.

To achieve our study’s goal, we conducted a 3-phase mixed methods study, which is now mainstream in health services research [30]. It offers “an overarching methodological framework to a multiyear project” where the ability to build on what was learned previously is paramount [31]. Its purpose “is to address a set of incremental research questions” that all advance a broader research goal [31]. As Figure 2 shows, the 3 phases—concept mapping, content validity assessment, and interrater reliability assessment—were sequentially aligned to iteratively collect, analyze, and combine the quantitative and qualitative data needed for each incremental research objective (described in the following sections). Throughout the study, we placed a greater emphasis on quantitative methods as this is recommended when qualitative data supplement the development of robust instruments [31]. Our study is reported following the Mixed Methods Research checklist [30] (the study protocol is available elsewhere [32]).

### Phase 1: Concept Mapping

#### Overview

The objective of phase 1 was to identify responsibility principles and best practices specific to DAI solutions missing from the original RIH tool. This phase relied on our scoping review [17] and concept mapping, which refers to a “structured process” that gathers “input from multiple participants” and uses qualitative pattern matching and quantitative multivariate analyses to produce an exhaustive map of a conceptual domain [33]. The 3-step process we followed to generate, structure, and represent “as completely as possible all of the key facets” [33] of responsibility in DAI health solutions (ie, the conceptual domain of interest in our study) is fully described in Multimedia Appendix 2 [3,12-17,19,21-23,33-48].

#### Data Analyses

Following an accountable qualitative thematic analysis strategy [34], LR and RRO categorized each principle found in the 56 tools included in the scoping review using the definitions provided by their authors. The quantitative analyses first examined the distribution of the principles found across the tools, which shed light on the responsibility constructs that they prioritized or disregarded. We then stratified the analyses along 3 subsets of tools—those from academia, governments, and the business sector—and applied a network analysis [35]. The aim was to examine the connection patterns between the tools and the principles they promoted (ie, “Principle A” is linked to “Tool 1” when the latter relies on that principle). By calculating the degree of centrality (in-degree), which represents the proportion of connections that a principle has compared with all possible connections it may have with the other tools in the subset, we obtained a ranking order in which more than one principle could occupy the same position. This facilitated a systematic comparison of the responsibility constructs that these tools sought to operationalize through questions, recommendations, criteria, and “dos and don’ts,” among other things.

Then, 4 researchers (LR, RRO, PL, and an AI ethics expert) independently mapped all principles across the RIH tool’s components: (1) premises (how responsibility is defined for the purposes of the tool), (2) screening criteria (baseline responsibility requirements), and (3) assessment attributes (degree to which responsibility characteristics are present). Each team member examined what type of revision was needed to capture the responsibility principles specific to DAI solutions (eg, modifying an existing premise, eliminating it, or adding a new one). Finally, we identified through team deliberations adaptations that covered the principles already captured in the RIH tool (eg, revising the “Ethical, legal, and social issues” attribute to cover specific data-related consent and compliance issues), those that could be aggregated (eg, antidiscrimination and fairness), and those not captured in the RIH tool that called for new attributes (eg, interoperability and data governance).

### Phase 2: Content Validity Assessment

#### Overview

The objective of phase 2 was to ensure the tool’s content validity, which refers to “the degree to which the content of an instrument is an adequate reflection of the construct to be measured” [49]. It relied on an international 2-round e-Delphi exercise [22]. Delphi research techniques are often used in emerging clinical areas of practice that “span multiple areas of expertise” and where consensual guidelines are lacking [50]. For a multidisciplinary panel of international experts to critically assess and improve the content validity [49] of the first version of the tool, the round 1 survey of our e-Delphi exercise comprised 22 closed-ended items using a 5-level Likert scale and 22 free-text boxes for experts to explain their ratings (excluding the research ethics consent form and demographic survey items). As the original RIH components had already been validated, the round 1 survey focused on the importance, clarity, applicability, and comprehensiveness of the new components specific to DAI solutions (indicated in Figure 1). On the basis of the results of round 1, a revised version of the tool was developed for round 2, which comprised 20 closed-ended items and 20 free-text boxes. It excluded items for which consensus had been reached and introduced the scales associated with each attribute (the surveys can be found in Multimedia Appendix 2). After each round, personalized feedback with individual responses and measures of central tendency, as well as the panel comments, was sent to each expert [22].

#### Data Analyses

Three measures had to be met to reach consensus: (1) at least 51% of experts scoring the item on the 2 highest levels (4 and 5), (2) an SD of ≤1.5, and (3) an IQR of ≤1.0 [22]. We applied a rigorous qualitative thematic approach [34] to analyze free-text responses. In total, 3 researchers (LR, RRO, and PL) independently categorized the comments, made proposals to address them, and then agreed on the changes required to improve the tool.

### Phase 3: Interrater Reliability Assessment

#### Overview

The objective of phase 3 was to assess the reliability of the tool by measuring interrater agreement and suggest measurement revisions if needed [23]. Interrater reliability refers to the extent to which 2 or more raters classify the same set of objects in the same way [36]. Following the recommendations by Gwet [36] on the number of objects required to achieve a sufficient level of accuracy and minimize the percentage of agreement SE, an error margin of –0.20 to +0.20 was used to determine our sample size, that is, 25 DAI solutions. We first identified 45 real-world solutions, gathered publicly available information about them, and proceeded in a stepwise fashion to create a balanced and diversified sample. We selected solutions operating with or without AI, pursuing different purposes (eg, self-management, diagnosis, treatment, and administration), developed by diverse organizations (for-profit, not-for-profit, governmental, and nongovernmental organizations), and used in different contexts of care and regions. For the 2 raters (RRO and LR) to apply the tool as intended, we searched each solution’s website to collect information addressing the tool’s criteria and attributes (terms of reference, privacy or sustainability policy, user guides, governance structure, and annual reports). We tabulated relevant excerpts from all 25 DAI solutions in a Microsoft Excel (Microsoft Corp) “scorecard” that both raters completed independently. As start-ups tended to share less detailed documentation than large firms, PL adapted the content found on other developers’ websites for the scorecard to contain all the information needed to score each criterion and attribute for all 25 solutions (Multimedia Appendix 2).

#### Data Analyses

Once each rater had independently completed their assessment, we calculated (1) a nonadjusted index (percentage of agreement), (2) a more paradox-resistant chance-adjusted index (the Gwet agreement coefficient), (3) SEs, (4) 95% CIs, and (5) *P* values [23]. The interpretation of the strength of the Gwet agreement coefficient, where 1 represents maximum reliability and 0 represents no reliability, follows the Landis-Koch scale [51]: poor (<0.0), slight (0.0-0.20), fair (0.21-0.40), moderate (0.41-0.60), substantial (0.61-0.80), and almost perfect (0.81-1.00). Finally, a fourth team member (HPS) chaired a meeting for the 2 raters to deliberate over diverging scores, reach consensus, and identify final minor improvements to the tool.

### Ethics Approval

Ethics approval was obtained from the Health Sciences Research Ethics Review Board of the Université de Montréal (CERSES-20-144-D).

## Results

### Phase 1: Tool Comprehensiveness

**Figure 3:**
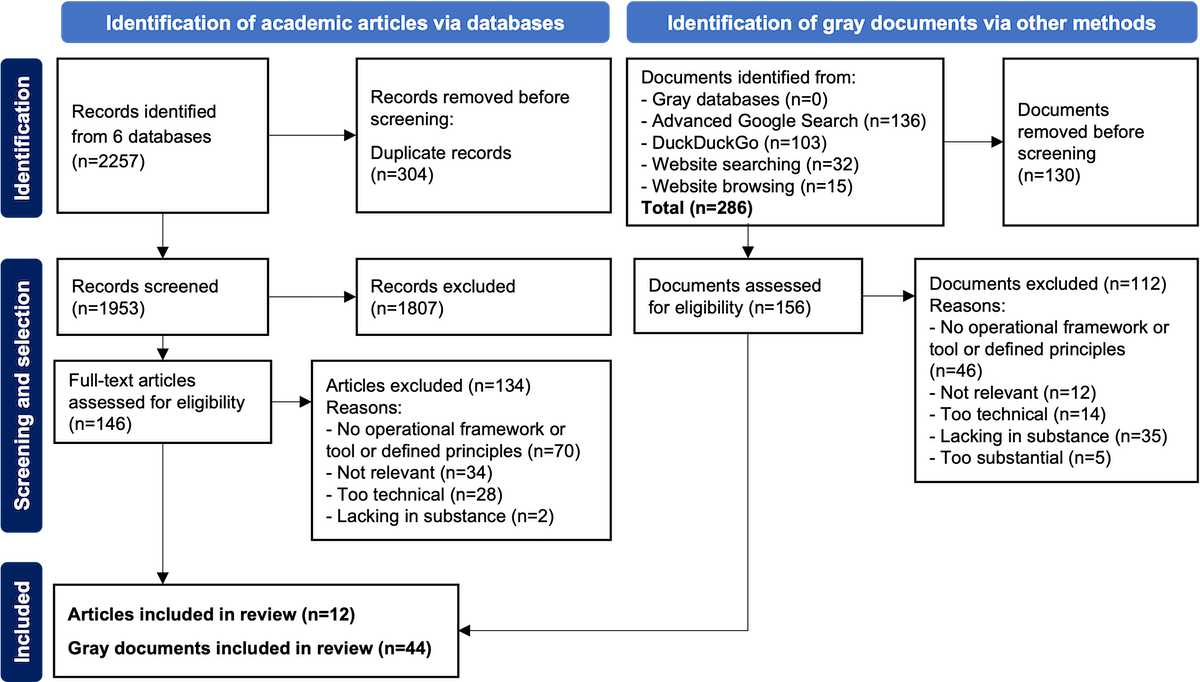
The scoping review flowchart following the PRISMA-ScR (Preferred Reporting Items for Systematic Reviews and Meta-Analyses extension for Scoping Reviews) guidelines.

Although the scoping review data set is available elsewhere [17], the databases used as well as the inclusion and exclusion criteria are described in Multimedia Appendix 2. Figure 3 [17] summarizes the selection process following the PRISMA-ScR (Preferred Reporting Items for Systematic Reviews and Meta-Analyses extension for Scoping Reviews) [52]. We retained a total of 56 tools, 12 (21%) from academic literature and 44 (79%) from gray literature.

**Figure 4:**
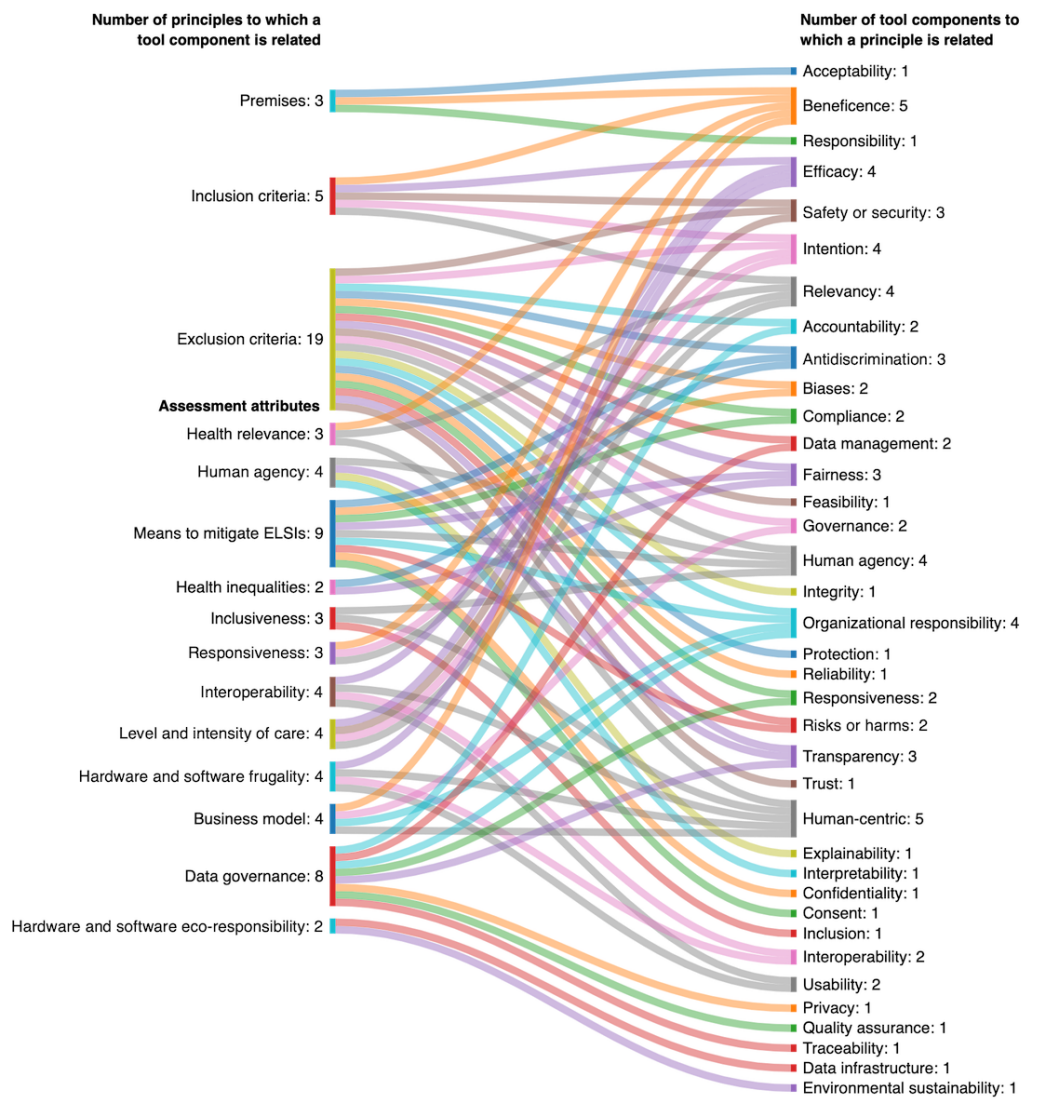
Results of the mapping process leading to the first version of the tool. Graphic created using SankeyMATIC. Premises included responsibility is linked to the context of use, responsibility means aiming for collective benefits, AI for good is not automatically responsible, and digital literacies and Internet connectivity are “superdeterminants” of health. Among the inclusion criteria were DAI solution definition and relevance of digitalization. The exclusion criteria included general availability stage not reached, data reselling as primary business model, deliberately deceptive solutions, AI relying on biased data sets, and lack of cybersecurity and personal data protection. The term Human-centered interoperability was used in the first version of the tool. ELSI: ethical, legal, and social issues.

Figure 4 illustrates the results of the mapping process that led to the first version of the tool. It shows how the 40 principles identified after systematically coding each tool are linked to the tool’s premises, inclusion and exclusion criteria, and assessment attributes. After team deliberation, we revised 2 RIH premises and introduced 2 new ones (“AI for good is not automatically responsible” and “Relevance of digitalization”). We revised all RIH inclusion criteria and added a new exclusion criterion that covered 4 areas considered particularly problematic in DAI solutions (“Data reselling as the primary business model,” “Deliberately deceptive solution,” “Lack of cybersecurity and personal data protection,” and “AI relying on biased datasets”). We revised existing RIH attributes and integrated 3 new attributes: “Human agency,” “Interoperability,” and “Data governance.” Finally, we adapted the RIH frugality and eco-responsibility attributes to account for both software and hardware that may be required to operate a DAI solution.

### Phase 2: Tool Content Validity

**Figure 5:**
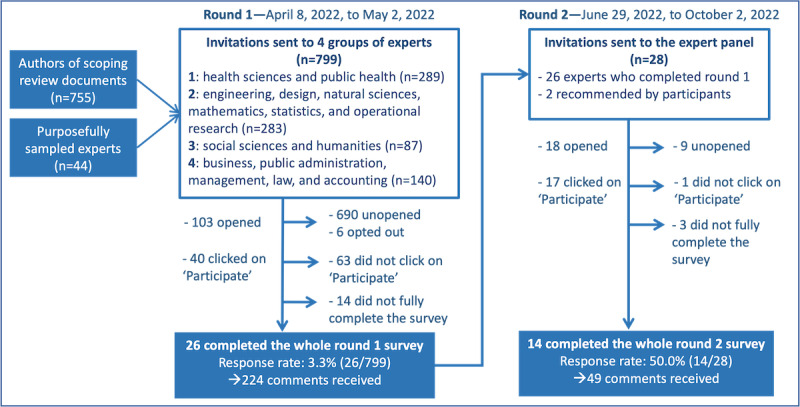
e-Delphi expert panel selection and data collection flowchart.

Figure 5 shows the flowchart of the e-Delphi expert solicitation process, which began in April 2022. The information sources used to identify experts in 4 main disciplinary fields—health sciences, engineering and computer sciences, social sciences, and administration and law—are described in Multimedia Appendix 2. A total of 799 invitations were sent to authors of documents screened during the phase 1 scoping review (n=755, 94.5%) and to purposefully sampled experts (n=44, 5.5%). After 3 reminders and excluding surveys not fully completed, 26 experts participated in the round 1 survey, with 224 comments and a 3.3% (26/799) response rate. Between June 2022 and October 2022, a total of 14 experts completed the round 2 survey, with 49 comments and a 50% (14/28) response rate.

Table 1 describes the final panel composition, which included a similar proportion of men and women (13/26, 50% and 12/26, 46% in round 1 and 6/14, 43% and 6/14, 43% in round 2, respectively). A well-balanced representation across the 4 disciplinary fields was observed in round 1 (ranging from 5/26, 19% to 7/26, 27%). In round 2, a higher participation of social scientists (6/14, 43%) and health scientists (4/14, 29%) was observed, whereas a similar proportion (2/14, 14%) of engineers and computer scientists and of administration and law experts completed the survey. Most experts had >10 years of experience (17/26, 65% in round 1 and 9/14, 64% in round 2) and were employed in higher education institutions (21/26, 81% in round 1 and 12/14, 86% in round 2) in North America (16/26, 62% in round 1 and 7/14, 50% in round 2).

Table 2 presents the results of the e-Delphi round 1 and round 2 surveys. In round 1, consensus was reached on 27% (6/22) of the survey items pertaining to the first version of the tool: the importance of 1 premise (“Context of use”), the applicability of 1 screening criterion (“DAI solution definition”), the importance of the “Human agency” attribute, the importance and clarity of the “Data governance” attribute, and the clarity of the “Programming and software eco-responsibility” attribute. On the basis of the comments received, which can be found in Multimedia Appendix 2, we withdrew 1 premise (“AI for good”) and 1 screening criterion (“Relevance of digitalization”); formulated 1 new premise (“DAI solutions should tangibly improve current processes and means”); revised all remaining premises, criteria, and attributes; and developed the scales for all attributes. In round 2, consensus was reached on 80% (16/20) of the items surveyed for the second version of the tool. Experts agreed on the importance and clarity of all premises except for the clarity of “DAI solutions affect the determinants of health.” They agreed on the applicability of 1 screening criterion (“GA stage not reached”) but not on the applicability of “Nondisclosure of key DAI risks.” Consensus was reached on the clarity, importance, and appropriateness of the scales of all assessment attributes except for the clarity of “Human-centered interoperability” and for the appropriateness of the scale of “Programming and software eco-responsibility.” Overall, the content validity of 85% (22/26) of the items surveyed was confirmed after round 2. The comments received enabled our team to generate a third version of the tool that addressed all round 2 experts’ criticisms (see our responses in Multimedia Appendix 2).

**Table 1:** The e-Delphi panel composition.

Characteristic		Round 1 (n=26), n (%)	Round 2 (n=14), n (%)
**Gender**			
	Woman (including cisgender and transgender)	12 (46)	6 (43)
	Man (including cisgender and transgender)	13 (50)	6 (43)
	Undisclosed or no response	1 (4)	2 (14)
**Discipline**			
	Health sciences or public health	5 (19)	4 (29)
	Engineering, computer sciences or data sciences, design, natural sciences, mathematics, statistics, or operational research	5 (19)	2 (14)
	Social sciences or humanities	7 (27)	6 (43)
	Business, public administration or management, and law or accounting	6 (23)	2 (14)
	Multidisciplinary field	3 (12)	0 (0)
**Years of experience**			
	<5	4 (15)	1 (7)
	5-10	5 (19)	4 (29)
	>10	17 (65)	9 (64)
**Primary employer**			
	Higher education	21 (81)	12 (86)
	For-profit organization, consultant firm, or privately funded research institution	3 (12)	1 (7)
	Government or arm’s length public administration agency	1 (4)	0 (0)
	Health care facility	1 (4)	1 (7)
**Region**			
	North America	16 (62)	7 (50)
	Europe	7 (27)	4 (29)
	Asia	1 (4)	1 (7)
	Oceania	2 (8)	2 (14)

**Table 2:** Results of the e-Delphi at round 1 and round 2^a^.

Survey item				Score of 4 to 5, n (%)					Values, SD					Values, IQR					Consensus status		
				Round 1		Round 2		Round 1			Round 2		Round 1			Round 2		Round 1			Round 2
**Premises (shortened for clarity)**																					
	**Context of use**																				
		Importance	23 (92)		N/A^b^		0.6			N/A		1.0			N/A		✓			N/A	
		Clarity	12 (46)		10 (100)		1.2			0.4		1.8			0					✓	
	**Collective benefits**																				
		Importance	18 (69)		9 (90)		1.1			0.7		2.0			0					✓	
		Clarity	17 (65)		8 (80)		1.1			1.2		2.0			1.0					✓	
	**AI^c^ for good (withdrawn in round 2)**																				
		Importance	16 (62)		N/A		1.2			N/A		2.0			N/A					N/A	
		Clarity	13 (50)		N/A		1.4			N/A		2.8			N/A					N/A	
	**Digital determinants of health**																				
		Importance	17 (65)		8 (89)		1.5			0.7		2.0			0					✓	
		Clarity	19 (73)		7 (70)		1.0			1.4		1.8			1.8						
	**Tangible improvements**																				
		Importance	N/A		10 (100)		N/A			0.5		N/A			0.8		N/A			✓	
		Clarity	N/A		9 (90)		N/A			1.3		N/A			1.0		N/A			✓	
**Screening step inclusion and exclusion criteria**																					
	**DAI solution^d^**																				
		Applicability	21 (81)		N/A		0.9			N/A		1.0			N/A		✓			N/A	
	**Relevance of digitalization (withdrawn in round 2)**																				
		Applicability	17 (65)		N/A		1.2			N/A		2.0			N/A					N/A	
	**GA^e^ stage not reached**																				
		Applicability	13 (50)		13 (93)		1.0			0.6		2.0			1.0					✓	
	**Nondisclosure of key DAI solutions risks**																				
		Applicability	17 (65)		10 (71)		1.2			1.2		2.0			1.8						
**Assessment step attributes**																					
	**Human agency**																				
		Importance	25 (96)		N/A		0.6			N/A		1.0			N/A		✓			N/A	
		Clarity	18 (69)		10 (83)		1.0			0.9		2.0			0.3					✓	
		Appropriate scale	N/A		11 (85)		N/A			0.8		N/A			1.0		N/A			✓	
	**Human-centered interoperability (round 1); care-centric interoperability (round 2)**																				
		Importance	19 (73)		11 (100)		1.0			0.5		1.8			0.5					✓	
		Clarity	18 (69)		9 (75)		1.0			1.0		2.0			1.3						
		Appropriate scale	N/A		9 (82)		N/A			0.8		N/A			1.0		N/A			✓	
	**Software frugality**																				
		Importance	19 (73)		10 (91)		0.8			0.9		1.8			1.0					✓	
		Clarity	18 (69)		11 (92)		0.9			0.9		2.0			1.0					✓	
		Appropriate scale	N/A		9 (82)		N/A			1.0		N/A			1.0		N/A			✓	
	**Data governance**																				
		Importance	25 (96)		N/A		0.7			N/A		1.0			N/A		✓			N/A	
		Clarity	21 (81)		N/A		0.9			N/A		1.0			N/A		✓			N/A	
		Appropriate scale	N/A		11 (92)		N/A			0.9		N/A			1.0		N/A			✓	
	**Programming and software eco-responsibility**																				
		Importance	18 (69)		10 (83)		1.0			1.2		2.0			1.0					✓	
		Clarity	21 (81)		N/A		0.9			N/A		1.0			N/A		✓			N/A	
		Appropriate scale	N/A		9 (75)		N/A			1.0		N/A			1.3		N/A				

^a^The survey items were formulated as follows: How important is this premise/criterion/attribute? How applicable is this criterion? Is this premise/attribute clearly defined? Is the scale appropriate?

^b^N/A: not applicable.

^c^AI: artificial intelligence.

^d^DAI solutions: digital health solutions that operate with or without artificial intelligence.

^e^GA: general availability.

### Phase 3: Tool Reliability

A description of the 25 DAI health solutions selected for assessing the tool’s reliability can be found in Multimedia Appendix 2. The sample comprised 52% (13/25) of solutions that operated with AI (eg, a wayfinding app for persons living with cognitive or physical impairment combining GPS technology and AI and an AI-based diabetic retinopathy screening system) and 48% (12/25) that operated without AI (eg, a platform to develop customized apps for health care facilities with limited digital infrastructures and a virtual reality–based treatment for individuals living with chronic lower back pain). An equal number of solutions (5/25, 20%) supported prevention, self-care, diagnostics, treatment, or administration. In total, 32% (8/25) of the solutions were designed to be used in a clinical setting only, 52% (13/25) were designed to be used in a nonclinical setting only, and 16% (4/25) were designed to be used in both settings. A total of 12% (3/25) of the solutions were developed by governmental agencies or user-led associations, 32% (8/25) were developed by not-for-profit organizations (universities and nongovernmental organizations), and 56% (14/25) were developed by for-profit organizations. According to their developers’ websites, 56% (14/25) of the solutions were in use in more than one continent.

Table 3 shows the results of the interrater reliability assessment (the data set is available in Multimedia Appendix 2). For screening criteria, an *almost perfect* agreement was found for “DAI solution,” for 2 subcriteria of “Nondisclosure of DAI risks” applicable to all DAI solutions, and for “GA stage not reached.” A “substantial agreement” was obtained for the “Nondisclosure of DAI risks” subcriterion applicable only to AI solutions. As we first reached a “moderate agreement” for “Human agency” (results can be found in Multimedia Appendix 2), we decided to revise its definition and perform a second interrater agreement. This was aligned with our objective of suggesting measurement revisions if needed. The reliability of the definitive version of the tool was high as an “almost perfect” agreement was obtained for all assessment attributes.

**Table 3:** Results of the interrater reliability assessment (N=25)^a^.

Survey item		Agreement (%), SE (95% CI)	DAI solutions^b^ (%)	*P* value	Gwet AC_1_^c^ or AC_2_^d^ coefficient (SE; 95% CI)	*P* value	Interpretation
**Screening step**							
	DAI solution definition	0 (1-1)	100	N/A^e^	1 (0; 1-1)	N/A	Almost perfect
	Nondisclosure of key risks	0 (1-1)	100	N/A	1 (0; 1-1)	N/A	Almost perfect
	Nondisclosure of key risks (applicable to AI^f^ only)	0.10415 (0.619-1)	85	<.001	0.792 (0.1602; 0.443-1)	<.001	Substantial
	GA^g^ stage not reached	0 (1-1)	100	N/A	1 (0; 1-1)	N/A	Almost perfect
**Assessment step**							
	Human agency (revised)	0.01021 (0.966-1)	99	<.001	0.964 (0.02946; 0.903-1)	<.001	Almost perfect
	Care-centric interoperability	0.02408 (0.905-1)	96	<.001	0.865 (0.07190; 0.717-1)	<.001	Almost perfect
	Software frugality	0.01992 (0.899-0.981)	94	<.001	0.837 (0.05622; 0.721-0.953)	<.001	Almost perfect
	Data governance	0.02552 (0.897-1)	95	<.001	0.881 (0.06450: 0.748-1)	<.001	Almost perfect
	Programming and software eco-responsibility	0.00245 (0.992-1)	100	<.001	0.994 (0.00594; 0.982-1)	<.001	Almost perfect

^a^Gwet first-order agreement coefficient is shown for the nominal ratings of the screening criteria (yes or no), and Gwet second-order agreement coefficient is shown for the ordinal ratings of the assessment attributes (A, B, C, and D). We used unweighted coefficients for nominal ratings and weighted coefficients for ordinal ratings (using quadratic weights). Interpretation follows the Landis-Koch scale: 0.8 to 1=almost perfect; 0.6 to 0.8=substantial; 0.4 to 0.6=moderate; 0.2 to 0.4=fair; 0 to 0.2=slight; and <0=poor [51]. The results of the first interrater agreement for “Human agency” as well as the changes made to this attribute can be found in Multimedia Appendix 2.

^b^DAI solutions: digital health solutions that operate with or without artificial intelligence.

^c^AC_1_: first-order agreement coefficient.

^d^AC_2_: second-order agreement coefficient.

^e^N/A: not applicable.

^f^AI: artificial intelligence.

^g^GA: general availability.

## Discussion

### Principal Findings and Comparison With Prior Work

Considering that the current biggest challenges to health are at the interface of climate change and growing inequalities [11] and that the carbon footprint of digital services is increasing by 8% annually [53], this study’s contributions are 3-fold.

#### Informing the Responsible Design and Adoption of DAI Solutions

First, the Responsible DAI solutions Assessment Tool is among the first tools to offer a comprehensive, valid, and reliable means to measure the degree of responsibility of DAI health solutions that can be applied by clinicians and other health innovation stakeholders. On the one hand, it can inform “supply side” decisions made by those who design DAI solutions, such as data scientists, programmers, clinical investigators, entrepreneurs, investors, research funders, and incubators. On the other hand, it can inform “demand side” decisions, including those of purchasers, implementers, patients, clinicians, and health care managers (see the “Who can apply the Tool and how?” section in Multimedia Appendix 1). Although the RIH framework and tool have been used to analyze responsibility challenges of DAI solutions [21,23,27], scholars, clinicians, and decision makers have been calling for a concise tool that could also account for issues specific to DAI health solutions [15,37].

#### Screening of Baseline Responsibility Requirements

Second, the tool’s 3-step application process enables clinicians to swiftly screen whether a DAI solution lacks *baseline* responsibility requirements before proceeding to a full assessment. For instance, 1 of the 5 screening criteria requires documenting whether “the DAI solution has been proven effective and safe to human health” by using publicly available evidence such as peer-reviewed scientific articles or reports by regulatory agencies (see the “Sources of information to look for before applying the Tool” section in Multimedia Appendix 1). Here, the assumption is that, if “must-have” requirements such as safety and effectiveness are not met, the solution cannot be considered responsible and, thus, there is little value in further assessing the extent to which responsibility attributes may or may not be fulfilled (unless the intent is to use the tool to improve the solution; see the following sections). A similar logic applies to the exclusion criterion “Nondisclosure of key DAI risks,” but in this case, information sources that may be used to apply the tool are those made publicly available by solution developers (eg, terms and conditions statement, data protection, and privacy policies). This criterion examines whether the organization that makes the DAI solution available to end users refrains from selling user-related data [54]; makes explicit its cybersecurity, privacy, and personal data protection measures; and clearly communicates how potential biases in the data set used to train an AI were mitigated (when applicable) [55]. Acknowledging that such information sources are of lower quality, the tool nonetheless strongly encourages solution developers (ie, data scientists, programmers, entrepreneurs, and high-level executives) to make their commitments to responsible DAI solutions explicit and, thus, accountable [5]. This seems particularly important as “patients and clinicians struggle to select digital health tools in an environment with inconsistent regulation and sparse information” on their risks; benefits; and ethical, legal, and social issues [38].

#### An Integrated Set of RIH Attributes Specific to DAI Solutions: From Human Agency to Eco-Responsibility

Third, the tool’s new attributes and their descriptive mutually exclusive scales can help clinicians identify and compare the degree of responsibility of different DAI solutions. For Obermeyer and Topol [6], the technical choices and human values underpinning the training of AI can either “scale up” biases based on socially determined characteristics such as race and gender or help “fight against” them. The “Human agency” attribute provides further practical guidance as its scale describes 4 concrete agency *enablers* that a DAI solution can proactively embed in its design and use. These enablers should help clinicians and patients (1) understand the measures, recommendations, decisions, or outputs of a DAI solution (eg, data visualization and transparency if an AI-based solution is unexplainable [14]); (2) discuss their implications with managers or staff when needed (eg, dedicated point of service); (3) act in accordance with their own goals without undue pressure (eg, freedom to override an AI-based decision [39]); and (4) have their concerns acted upon through an appeal, audit, review, or redress mechanism (eg, ombudsman [38]). This new attribute is aligned with recent efforts to define the “minimum information” required for users to better understand the “intended predictions, target populations, and hidden biases” of DAI solutions (see the Minimum Information for Medical AI Reporting) [56]. It also supplements other key RIH attributes in striving to reduce avoidable health status differences across individuals and groups (“Health inequalities”), avoid user parameters that preclude legal rights to be exercised (“Mitigation of ELSIs”), and overcome a poor understanding of different users’ varying needs (“Inclusiveness”) [40,57].

The “Care-centric interoperability” attribute refers to how smoothly a DAI solution can securely operate within and across clinical and nonclinical settings without adding cognitive or administrative burden to users [15]. It is based on a broader understanding of the interoperability standards promoted for a safe integration and interfacing of digital and nondigital devices in a health system [1]. Its scale stresses four *characteristics* that can be embedded in a DAI solution design: (1) aligning the solution with its users’ data management practices (and not vice versa) to minimize cognitive and administrative burden, (2) aligning the solution with its users’ digital infrastructures (eg, operable on widely available systems and devices [3,41]), (3) incorporating data sharing functionalities that “follow the patient” along clinical pathways or practitioners’ work processes (eg, nonproprietary software and data portability [58]), and (4) ensuring that it can securely evolve with users’ digital infrastructures (eg, built-in security features in software as a service and auditable logs [59]).

The “Software frugality” attribute refers to the ability to deliver greater value to more people by using fewer resources, such as capital, materials, energy, and labor time [19]. Frugal innovation may be easily overlooked in the health care sector, but it clearly matters to the future of health systems [60,61]. Grounded in an up-to-date scientific understanding, this attribute recognizes that frugality is not about creating “the cheapest products” [62]; rather, it is about increasing their economic value by designing high-quality solutions that are affordable and usable and fit with their context of use. The scale of this attribute stresses that responsible software should meet three frugal innovation *characteristics* [63]: (1) affordability (which may result from software development strategies; open-source programming tools; or low technical support, update, and maintenance needs [27]), (2) focus on core user-facing functionalities that meet a larger number of user capabilities (eg, universal interface design for users with low literacy), and (3) maximized fit between functionalities and user location–dependent digital capacities [58] (eg, edge computing for settings where connectivity is compromised).

The “Data governance” attribute responds to a widely shared consensus among scientific [4] and policy [3] communities for proper oversight of data. It refers to the stewardship, structures, and processes that an organization sets in place to ensure full control over the entire data life cycle. The scale brings forward four *mechanisms* that can be combined to support responsible data governance: (1) a chief data officer or committee accountable for the way employees gather, exploit, generate, store, share (voluntarily or not), or destroy data and for any data-related breaches or incidents [3]; (2) a training program for managers and employees to remain up-to-date and properly skilled in data management; (3) data protection practices relying on performance indicators or standards (eg, ISO/IEC 27001: information security management and ISO/TS 82304-2: quality and reliability of health and wellness apps [38]); and (4) an auditable data governance reporting system [39,59].

Finally, 2 distinct attributes were created to fully capture the environmental harms arising from hardware on the one hand and from programming and software on the other. “Programming and software eco-responsibility” refers to a product, process, or method that reduces as much as possible the negative environmental impacts. It spans the use of clean energy sources and the reduction of the energy consumed when developing AI and software and archiving data. The scale of this attribute highlights three eco-responsible *practices*: (1) choosing programming, modeling, or computational techniques that substantially reduce the quantity of energy and time required to develop a DAI solution (eg, TinyML); (2) using highly energy-efficient central processing units and computers; and (3) selecting data centers and server farms where greenhouse gas emissions are reduced to a minimum (net zero) or where more greenhouse gases are removed from the atmosphere than emitted (climate positive) [12,16]. This adaptation of the RIH tool responds to current knowledge on the environmental impacts of the ever-increasing energy demands of complex data computational practices, including the training of algorithms [7], and of the growing use of digital devices that consume rare-earth metals and have harmful end-of-life disposal outcomes [64]. Given the numerous hardware components that may surround a DAI solution, the tool clearly indicates whether the attributes “Hardware frugality” and “Hardware eco-responsibility” apply (see the “Scope of the assessment” section in Multimedia Appendix 1). Hardware equipment should be included in the assessment when its raison d’être is to support the DAI solution *and* it is part of the minimal requirements for the solution to deliver its service. For instance, a finger sensor used to record an ECG using a smartphone fulfills these 2 criteria but not the smartphone. Similarly, surgical robot hardware components meet the 2 criteria (their raison d’être is to support the surgical procedure, and the latter would not be possible without them) [50]. Attending to hardware eco-responsibility concerns implies reducing environmental harms at key *stages* in a product’s life cycle, which include (1) raw material sourcing (eg, free of substances that are harmful and toxic to ecosystems), (2) manufacturing (eg, compliance with national or international environmental regulations), (3) distribution (eg, packaging and transportation), (4) use (eg, durability and repairability), and (5) disposal (eg, designed to be recycled, disassembled, remanufactured, composted, or biologically degraded) [65].

### Implications for Practice

This concise yet comprehensive forward-looking tool is not without limitations, but it has the potential to change both thinking and practice in the rapidly evolving field of DAI health solutions. These solutions may drive many improvements in health care [6]. Nevertheless, the pace at which they are being developed remains unprecedented when compared with other medical advances such as minimally invasive surgery, interventional radiology, or genomics [5]. Although many scholars underscore that DAI solutions should be used “in compliance with relevant laws” [4], regulatory frameworks remain scant, and policy progresses are slow [3]. Current regulatory and policy limitations and the lack of robust assessment tools put patients, clinicians, and health care managers at risk [4] not only of biases but also of diversion from health systems’ key mission: improving health in an economically and environmentally sustainable way [26]. As many decisions driving the supply of DAI solutions are made outside the health sector [38], clinicians are currently largely unequipped to anticipate and handle their health, social, economic, and environmental impacts [7].

The tool was specifically designed to support clinicians in the broader role they should play as “change agents” [4] in the digital health field. Thanks to a multidisciplinary expert panel, the tool’s attributes are clearly defined, and its scales describe key responsibility enablers, characteristics, or mechanisms in a tangible way. Its practical value lies in the fact that it can be applied in two distinct ways: (1) as a formal evidence-informed assessment tool to measure the degree of responsibility of a DAI solution or (2) as a design or procurement brief (or template) to explore the suitability of a given DAI solution for patient care and clinical practice and guide its development, acquisition, implementation, or use. In both situations, the overall responsibility score is considered invalid if the screening criteria are not met. When used as a formal assessment tool, specific steps should be followed for the tool to deliver a valid score—after having searched, retrieved, and critically analyzed sources of information pertaining to each screening criterion and assessment attribute, an interdisciplinary team (2-5 raters with research skills) must first apply the tool independently and then reach consensus. As described in Multimedia Appendix 1, when disagreements between raters are found, the team should deliberate to identify potential errors or misunderstandings. The consensus score should neither be “forced” nor “averaged”—it should establish a strong correspondence between the information available and the question (for the screening criteria) or the scale item (for the assessment attributes) formulated in the tool.

Of course, one of the tool’s limitations lies in the information sources required to rate each criterion and attribute. Although the strongest sources of evidence remain independent peer-reviewed publications, few are likely to be available for an emerging DAI solution [6]. Moreover, the solution and the organization that makes it available to users may change over a brief period (eg, acquisitions of start-ups are frequent in the digital technology industry) [5]. The scope of these changes may significantly affect the adequacy of the scientific evidence available and the degree of responsibility of the solution. Therefore, those who apply the tool should remain critical of the information provided by developers and reconduct the assessment whenever significant changes are made to the solution or organization. As novel applications of AI keep emerging, such as generative AI that uses natural language processing to create textual content (eg, ChatGPT), it will be important to keep abreast of technological advances and apply the tool rigorously (ie, as described in Multimedia Appendix 1). Its definition of responsibility is anchored in the RIH scholarship, which largely differs from definitions found under the “responsible AI” umbrella term [42].

### Limitations

There are 3 limitations to this study that are partially mitigated by the strengths of a mixed methods study design [31]. When launching phase 1, we were challenged by the velocity at which tools to foster responsibility in DAI solutions had been developed (ranging from 3 in 2016 to 25 in 2020), and their quantity precluded an in-depth qualitative analysis of their conceptual overlaps. However, as 93% of the principles came from tools published before 2019, we are confident that significant principles have not been omitted [17]. In phase 2, we could not gather information about experts who ignored the invitation (690/799, 86.4%), did not click on “participate” (63/799, 7.9%), or did not complete the round 1 survey (14/799, 1.8%). However, the final panel size is adequate for an e-Delphi study, a high participation rate in round 2 (14/28, 50%) increases internal validity, and using 3 concurrent measures to determine consensus exceeds standards often seen in such studies [22]. In phase 3, objects and raters were not randomly selected, which limits the ability to draw inferences. The tool’s reliability is predicated on having raters sufficiently familiar with its premises, criteria, and attributes. An overarching strength of this 3-phase study was to have built on the scientific groundwork that led to the RIH tool [22,23]. Thus, our team had a good command of the methods needed for “measuring the constructs of primary interest” [31].

### Conclusions

Clinicians active in research have made great strides to work with DAI solution developers to address key clinical issues [6,43,44], and ground-breaking scholarly and policy work has brought to light the numerous ethical concerns that arise with their development and use [3,16,39,40]. However, tools developed to foster responsibility in DAI solutions focus on fragmented sets of principles, rarely offer measurable indicators, and lack methodological rigor [17]. Thus, we applied a rigorous study design to deliver a rigorous tool. Further actions include actively disseminating the tool through our research collaborators and developing multimedia materials to support its use [66]. Although strong clinical leadership is required for high-quality digital health care to materialize in practice, the Responsible DAI solutions Assessment Tool can help clinical leaders contribute to the design and use of DAI solutions with a high degree of responsibility. It offers a comprehensive, valid, and reliable means to help steer DAI solutions toward equitable as well as economically and environmentally sustainable digital health care.

## Appendix

Multimedia Appendix 1 Responsible DAI solutions Assessment Tool.

Multimedia Appendix 2 Methodological details and data sets.

## Abbreviations

AI: artificial intelligence
DAI solutions: digital health solutions that operate with or without artificial intelligence
PRISMA-ScR: Preferred Reporting Items for Systematic Reviews and Meta-Analyses extension for Scoping Reviews
RIH: Responsible Innovation in Health

## Data Availability

The data sets collected and analyzed are available in the body of the text or in Multimedia Appendix 2.
