# Supplementary material for: A Comprehensive, Valid, and Reliable Tool to Assess the Degree of Responsibility of Digital Health Solutions That Operate With or Without Artificial Intelligence: 3-Phase Mixed Methods Study
Source: J Med Internet Res. 2023 Aug 28;25:e48496. doi: 10.2196/48496 (PMC10495857; doi:10.2196/48496)
Supplement: Multimedia Appendix 2 [file jmir_v25i1e48496_app2.pdf]

Supplementary Material 2

Table of contents

A. Phase 1-Supplementary material 2
- A. 1. The concept mapping process leading to the first version of the Tool 2

B. Phase 2- Supplementary material 8
- B. 1. Sources used to identify four groups of experts for the e-Delphi panel 8
- B. 2. e-Delphi Round 1 survey 10
- B. 3. e-Delphi Round 2 survey 27
- B. 4. Dataset: Expert panel comments gathered at Round 1 40
- B. 5. Dataset: Expert panel comments gathered at Round 2 63

C. Phase 3- Supplementary material 72
- C. 1. Selection and documentation process of the D/AI solutions 72
- C. 2. Characteristics of the final sample of 25 D/AI solutions 75
- C. 3. Dataset: Raters' scores on each component of the Tool 77
- C. 4. Revision of the 'Human agency' attribute 78

A. Phase 1-Supplementary material

A. 1. The concept mapping process leading to the first version of the Tool

The objective of Phase 1 was to identify principles and best practices specific to D/AI solutions that could help us adapt the RIH Assessment Tool into the Responsible D/AI Solutions Assessment Tool. To do so, we followed a concept mapping process that relied on a scoping review our team published recently [17]. Concept mapping refers to a "structured process" that gathers "input from multiple participants" and uses qualitative as well as quantitative analyses to produce an exhaustive map of a conceptual domain [23]. To map "as completely as possible all of the key facets" [23] of responsibility in D/AI health solutions (i.e., the conceptual domain of interest in our study) and the relationships between key constructs, we followed the following three-step process:

1. Generating the conceptual domain;
2. Structuring the conceptual domain;
3. Representing the conceptual domain.

Before describing these steps, we explain below why the RIH Tool was used as the backbone to develop the Responsible D/AI Solutions Assessment Tool.

Rationale for adapting the RIH Tool

The RIH Tool is an evidence-informed tool that measures the degree of responsibility of a given health innovation along 9 attributes that capture product-, process-, and organization-level characteristics of responsibility [23]. It comprises 4 premises, 4 inclusion and exclusion criteria, 9 responsibility attributes with individual four-level Likert-like rating scales, and a scoring system that takes the quality of the sources of information used in the assessment into account. Construct validity and reliability were respectively established through a e-Delphi study conducted with 4 groups of international experts (health technology assessment, bioethics, biomedical engineering, and Responsible Research and Innovation) [22] and an interrater reliability assessment study [23]. To the best of our knowledge, this is one of the rare tools in the field of Responsible Research and Innovation (RRI) that is specific to the health sector and that establishes a quantitative measure of the degree of responsibility of a health innovation [21].

Though the RIH Tool is applicable to health innovations that contain D/AI components (e.g., data) [21], it does not capture specific responsibility issues that are raised by these components (e.g., data management). Since the development of the RIH framework [19] and its accompanying RIH Tool (within a seven-year research program funded by the Canadian Institutes of Health Research: 2015-2023), the field of digital and AI ethics has exponentially grown [37, 45, 46]. Ground-breaking advocacy, scholarly, and policy work has brought to light the numerous ethical and responsibility concerns that arise with the development and use of D/AI solutions, both in the health field and across other sectors. For instance, experts have made significant headway elucidating data management issues (e.g., biased datasets), the environmental footprint of D/AI solution development and use (e.g., data centres), AI governance mechanisms for organizations and businesses, and best practices for D/AI engineering professions [3, 12, 39, 15].

Because the state of the literature on responsible D/AI solutions now forms a solid body of knowledge and because the RIH Tool is scientifically sound in its structure and content, we aimed to adapt the RIH Tool to the specificities of D/AI solutions in health. Through a three-step adaptation process, we ensured that our

adaptations fit, both at a conceptual and a practical level, with the rationale, objective, and approach of the RIH Tool as well as with the realities of the rapidly evolving D/AI industry.

Step 1: Generating the conceptual domain: What key principles and best practices characterize responsible D/AI solutions?

To identify key D/AI responsibility principles and how they should be operationalized (through questions, recommendations, criteria, 'dos and don'ts,' etc.), we relied on our scoping review of practice-oriented tools that aim to guide the implementation of responsibility principles throughout the lifecycle of D/AI solutions [17]. Because many ethical principles (e.g., privacy, accountability, robustness, beneficence [3, 40, 45]) have been proposed, either specifically for health care [13] or for multiple sectors [14], and either for digital solutions [15] or for AI [16, 39], our search strategies were multidisciplinary and included the grey literature.

We searched 6 academic databases (PubMed, Web of Science, arXiv, Institute of Electrical and Electronics Engineers Xplore, IBSS Proquest Abstracts, Sociological Abstracts), 3 grey literature databases (OpenGrey, CMA CPG Infobase, Government of Canada Publications), and used 2 search engines (Advanced Google Search, DuckDuckGo) using key terms related to ethical AI (e.g., fairness, explainability), digital solutions (e.g., eHealth, mHealth), and RIH attributes (e.g., inequalities, environmental sustainability, inclusiveness, frugality). We included 1) practice-oriented tools defined as frameworks and/or sets of principles with clear explanations on how to apply them in practice; 2) applicable to solution design, development, assessment, use, or audit; 3) developed by academics, governments, NGOs, and/or reputable private sector sources; and 4) comprised of dimensions, criteria, and/or scales to reflect, judge, make a decision, identify, or report a responsibility challenge. We excluded tools that were 1) highly technical (e.g., code on Github); 2) highly specific (e.g., a cybersecurity measure); 3) very extensive (e.g., a fully detailed public procurement program); or 4) lacking in substance (e.g., a short blog post).

Our dataset comprised 56 tools stemming from the academic (n=12) and grey literature (n=44) and developed either specifically for the health field (n=19) or for generic applicability (n=37). Following a qualitative thematic analysis strategy [34], LR and RRO applied 40 codes, which are listed below, to identify the responsibility principles present in these tools. These codes as well as the definitions and practical recommendations provided by the tools' authors constituted the conceptual material to be structured in Step 2.

1. Acceptability
2. Accountability
3. Anti-discrimination
4. Beneficence
5. Biases
6. Compliance
7. Confidentiality
8. Consent
9. Data infrastructure
10. Data management
11. Efficacy
12. Environmental sustainability
13. Explainability
14. Fairness
15. Feasibility
16. Governance
17. Human-agency
18. Human-centric
19. Inclusion
20. Integrity
21. Intention
22. Interpretability
23. Interoperability
24. Liabilities & Sanctions
25. Organizational responsibility
26. Privacy
27. Protection
28. Quality assurance
29. Relevancy
30. Reliability
31. Reproducibility
32. Responsibility
33. Responsiveness
34. Risks/harms
35. Robustness
36. Safety/security
37. Traceability
38. Transparency
39. Trust
40. Usability

Step 2: Structuring the conceptual domain: What are the relationships between the principles and best practices aiming to foster responsible D/AI solutions?

To structure this conceptual material, we conducted different statistical analyses to define and visualize the relationships between the 40 principles. These quantitative analyses and their findings are reported in detail in our scoping review [17]. The descriptive analyses first examined the distribution of the principles across the 56 tools, which shed light on the responsibility constructs they prioritize or disregard. For instance, 'environmental sustainability' was not found in any of the health-specific tools (n=19) and 50% or more of all tools disregarded 21 principles. To get a better grasp of the key blind spots and normative preferences of the tool creators, we stratified the analyses along three subsets of tools —those from academia (n=15), governments (n=18), and the business sector (n=8)— and performed a network analysis. Table 1 indicates the results of these analyses, which provide a ranking order where more than one principle can occupy the same position. This facilitated a systematic comparison of the responsibility constructs these tools primarily sought to operationalize.

Academia (n=15)			Business sector (n=18)			Governments (n=8)		
**Rank**	**Principle**	**inDegree**	**Rank**	**Principle**	**inDegree**	**Rank**	**Principle**	**inDegree**
**1**	Beneficence	0.733	**1**	Privacy	0.889	**1**	Transparency	1.000
	Data management	0.733	**2**	Accountability	0.778	**2**	Data management	0.900
**2**	Privacy	0.667		Safety/security	0.778		Quality assurance	0.900
	Risks/harms	0.667		Transparency	0.778	**3**	Beneficence	0.800
**3**	Responsiveness	0.600	**3**	Compliance	0.722		Organizational responsibility	0.800
	Transparency	0.600		Data management	0.722		Risks/harms	0.800
**4**	Compliance	0.533		Fairness	0.722	**4**	Accountability	0.700
**5**	Consent	0.467		Human-centric	0.722		Biases	0.700
	Human-centric	0.467		Risks/harms	0.722		Compliance	0.700
	Quality assurance	0.467	**4**	Biases	0.667		Explainability	0.700
**6**	Biases	0.400		Quality assurance	0.667		Governance	0.700
	Fairness	0.400	**5**	Governance	0.611		Human-centric	0.700
	Human-agency	0.400		Human-agency	0.611		Safety/security	0.700
	Intention	0.400		Organizational responsibility	0.611	**5**	Human-agency	0.600
	Safety/security	0.400	**6**	Explainability	0.500		Inclusion	0.600
**7**	Accountability	0.333		Inclusion	0.500		Responsibility	0.600
	Governance	0.333		Responsibility	0.500	**6**	Fairness	0.500
	Inclusion	0.333		Responsiveness	0.500		Interpretability	0.500
	Organizational responsibility	0.333	**7**	Beneficence	0.444		Responsiveness	0.500
	Trust	0.333		Reliability	0.444		Usability	0.500
**8**	Data infrastructure	0.267	**8**	Consent	0.389	**7**	Confidentiality	0.400
	Efficacy	0.267		Integrity	0.389		Privacy	0.400
	Interpretability	0.267		Protection	0.389		Reliability	0.400
	Protection	0.267		Traceability	0.389	**8**	Anti-discrimination	0.300
**9**	Confidentiality	0.200		Trust	0.389		Consent	0.300
	Relevancy	0.200	**9**	Anti-discrimination	0.333		Efficacy	0.300
	Reliability	0.200		Interpretability	0.333		Liabilities/sanctions	0.300
**10**	Acceptability	0.133		Liabilities/sanctions	0.333		Relevancy	0.300
	Explainability	0.133	**10**	Environmental sustainability	0.278		Reproducibility	0.300
	Feasibility	0.133	**11**	Confidentiality	0.222		Robustness	0.300
	Integrity	0.133		Efficacy	0.222		Traceability	0.300
	Interoperability	0.133	**12**	Intention	0.111	**9**	Data infrastructure	0.200
	Responsibility	0.133		Reproducibility	0.111		Interoperability	0.200
	Usability	0.133		Robustness	0.111		Trust	0.200
**11**	Anti-discrimination	0.067		Usability	0.111	**10**	Environmental sustainability	0.100
	Irresponsibility	0.067	**13**	Acceptability	0.056		Intention	0.100
	Reproducibility	0.067		Data infrastructure	0.056			
	Robustness	0.067		Interoperability	0.056			

Table 1. Ranking of the principles found within three subsets of tools

In a network analysis, the prominence different tools give to certain principles can be revealed by looking at the connection patterns (‘links’) between different tools by identifying the principles (‘nodes’) that cooccur in the tools (i.e., ‘Principle A’ is linked to ‘Tool 1’ when the latter relies on that principle) [35]. We calculated the degree of centrality of the principles through a normalized inDegree, which represents the proportion of connections that a principle has compared to all possible connections it may have with the other tools in the network. It provides an indication of the relative importance of a principle within a subset of tools as it measures the extent to which a given principle is connected to other tools. The higher the inDegree is, the more influential the principle is within the network. Results from Table 1 thus facilitated a systematic comparison of the responsibility constructs these tools primarily sought to operationalize.

Step 3: Representing the conceptual domain: What adaptations are required and where shall they be introduced?

To develop the 1st version of the Responsible D/AI Solutions Assessment Tool, we followed an iterative and deliberative process led by PL. We began by mapping all the 40 principles to each key component of the RIH Tool (see Table 2, page 7):

- Its 4 premises, defining how responsibility is approached;
- Its 4 screening criteria, defining baseline requirements that must be met for an innovation to be eligible for assessment (i.e., to be considered potentially responsible); and
- Its 9 assessment attributes, which use a four-level scale to measure the degree to which a given responsibility characteristic is present.

To identify the adaptations the RIH Tool required to capture the D/AI responsibility principles, each component of the RIH Tool was isolated in a table and each team member (LR, RRO, PL, and an AI ethics expert) independently filled out their table to explain what type of revision was needed (e.g., modifying an existing premise, eliminating it, or adding a new one). We then copy-pasted each team member’s answers into a master table. On page 6, the ‘Business model’ and ‘Data governance’ attributes are used to illustrate how this exercise was structured: the definition was placed in the left column and proposed revisions highlighted in **red** in the right column, including additions, removals, and relocations of elements into other parts of the Tool. Relevant responsibility principles drawn from the scoping review were noted in **purple**. Through group discussions, we identified the main converging and diverging points and deliberated to reach consensus over the key adaptations required to generate a 1st version of the Responsible D/AI Solutions Assessment Tool.

This analytical process entailed going back to the definitions of the 40 principles and carefully examining the best practices recommended by the authors of the 56 tools (e.g., risks and issues to consider, indicators, criteria, or standards, technical, professional, or organizational guidelines). This content guided the revision of the existing RIH Tool attributes, for instance, making sure ‘Mitigation of ethical, legal, and social issues’ also cover specific personal information protection, user-related data consent, and compliance to regulatory frameworks. It also helped identify which principles could be aggregated without losing their specificity (e.g., anti-discrimination and fairness) and the principles that clearly called for the creation of new attributes (e.g., interoperability, data governance, human agency). Until the end of Phase 3, the team members discussed as a group and deliberated each new iteration, gradually confirming and refining each adaptation while ensuring coherence, consistency, clarity, as well as scope and fit with RIH and the D/AI solutions field (e.g., proper terms, applicability by stakeholders).

Assessment Attributes —Organizational Value Domain	Proposed revisions for new Tool
**Business model (original RIH attribute)**<br>Refers to the components through which an organization creates, delivers and captures social and economic value. A business model typically entails a tension between the redistribution of financial returns to shareholders and the provision of a high-quality innovation.<br>The business model of organizations that seek to provide more value to users, purchasers and society may possess the following **characteristics**:<br>• Pursue a social and/or environmental mission, operate on a not-for-profit basis or reinvest the majority of the revenues in their mission (e.g., social enterprises)<br>• Make the innovation freely usable or exploitable by others (i.e., open source, product licensing waivers, do-it-yourself)<br>• Adopt a pricing scheme based on ability to pay or a redistributive logic (e.g., customers who “buy one, give one”)<br>• Employ people with particular needs (e.g., low literacy, disabilities)<br>• Comply with social responsibility programs (e.g., Certified B Corporation, SA8000 standard for decent work, ISO26000 for social responsibility)<br>**The business model of the organization that produces the innovation possesses…**<br>Three of the characteristics described or more vs none	**Business model**<br>Refers to the components through which an organization creates, delivers and captures social and economic value. A business model typically entails a tension between the redistribution of financial returns to shareholders and the provision of a high-quality D/AI solution.<br>The business model of organizations that seek to provide more value to users, purchasers and society may possess the following **characteristics**:<br>• Pursue a social and/or environmental mission, operate on a not-for-profit basis or reinvest the majority of the revenues in their mission (e.g., social enterprises);<br>• Make the solution and its hardware components (see Scope of assessment) freely usable or exploitable by others (i.e., open source, product licensing waivers, do-it-yourself);<br>• Adopt a pricing scheme based on ability to pay or a redistributive logic (e.g., fees modulated according to user segments);<br>• Employ people with particular needs (e.g., low literacy, disabilities);<br>• Comply with social responsibility programs (e.g., Certified B Corporation, SA8000 standard for decent work, ISO26000 for social responsibility).<br>**The business model of the organization that makes the solution available to end users possesses…**<br>**Note:** Beneficence; Org. Responsibility; Governance; Human-centric

Data governance (Proposed additional attribute for new Tool)

Refers to the stewardship, structures and processes the organization sets in place to ensure full control over the entire lifecycle of the data it gathers, exploits, generates, stores and/or shares with users and third-parties (voluntarily or not). From data collection to data destruction, the organization and its leaders must remain transparent about, accountable for, and swiftly responsive to any breaches in data protection, and to any other issues affecting the D/AI solution data’s lifecycle.

Organizations producing responsible D/AI solutions make their high-level executives and employees knowledgeable about and able to report to external auditors on the sensitivity and scope of use of all datasets linked to their solutions. Mechanisms to achieve responsible data governance include:

- Fully active oversight committees whose members’ conflicts of interest are publicly declared;
- Explicit compliance to the laws and regulatory frameworks where users are located;
- Adherence to industry standards specific to D/AI solutions (e.g., ISO 13482:2014 for safety of personal care robots, ISO/TS 82304-2 for quality and reliability of health and wellness apps);
- Training programs and certification system for in-house data stewards;
- Fully functional and active reporting systems;

Data governance of the organization that makes the solution available to end users possesses…

(scale to be developed after the 1st construct validity step)

Note: Quality assurance; Accountability; Data management; Privacy; Responsiveness; Transparency; Org. responsibility

	Premises				Inclusion criteria		Exclusion criteria						Assessment attributes											
	Responsibility is linked to the context of use	Responsibility means aiming for collective benefits	AI for Good is not automatically responsible	Digital literacies & Internet connectivity are ' super-determinants' of health	Determinants of health	Relevance of digitalization	GA stage not reached	Irresponsible corporate actions	Data reselling as primary business model	Deliberately deceptive	Biased datasets	Lack of cybersecurity & personal data protection	Health relevance	Means to mitigate ELSIs	Health inequalities	Human agency	Inclusiveness	Responsiveness	Level & intensity of care	Human-centred interoperability	Frugality (hardware & software)	Business model	Data governance	Eco-responsibility (hardware & software)
Acceptability	√																							
Accountability								√															√	
Anti-discrimination											√			√	√									
Beneficence		√	√	√	√								√					√				√		
Biases											√			√										
Compliance							√							√										
Confidentiality														√										
Consent														√										
Data infrastructure																								√
Data management									√														√	
Efficacy					√														√	√	√			
Env. sustainability																								√
Explainability																√								
Fairness											√			√	√									
Feasibility							√																	
Governance								√				√										√		
Human-agency										√				√		√	√							
Human-centric													√				√			√	√	√		
Inclusion																	√							
Integrity										√														
Intention						√		√	√									√	√					
Interoperability																				√	√			
Interpretability																								
Liabilities/sanctions																√								
Org. responsibility								√	√	√				√								√	√	
Privacy																							√	
Protection										√	√	√												
Quality assurance																							√	
Relevancy						√							√					√	√					
Reliability							√																	
Reproducibility																								
Responsibility	√	√	√	√																				
Responsiveness								√															√	
Risks/harms										√	√	√		√										
Robustness																								
Safety/security					√		√												√					
Traceability																							√	
Transparency										√						√							√	
Trust								√		√		√												
Usability																				√	√			

Table 2. Linking the 40 principles to the 1st version of the Tool

B. Phase 2- Supplementary material

B. 1. Sources used to identify four groups of experts for the e-Delphi panel

The objective of Phase 2 was to ensure the Tool's content validity. To this end, experts from four broad disciplinary domains with high-quality publications in the D/AI solutions field were targeted: 1) health sciences and public health; 2) engineering, design, natural sciences, mathematics, statistics, and operational research; 3) social sciences and humanities; and 4) business, public administration, management, law, and accounting.

Ranking	Journal	Number of authors
1	Journal of Medical Internet Research	38
2	JMIR mHealth and uHealth	24
3	BMJ	22
	Human Genomics	22
5	Journal of the American Medical Informatics Association	18
6	Canadian Association of Radiologists Journal	17
7	Asian Bioethics Review	16
8	Annals of Rheumatic Diseases	14
	Methods of Information in Medicine	14
9	Academic Emergency Medicine	12
10	Science and Engineering Ethics	11
	Technological Forecasting & Social Change	11
	Digital Health	11
11	AIDS and Behavior	10
	Bulletin of the World Health Organization	10
	JMIR Mental Health	10
	BMC Medical Research Methodology	10
12	BMC Medical Informatics and Decision Making	9
	IEEE Access	9
	Journal of the American Academy of Child and Adolescent Psychiatry	9
13	Processes	8
	Journal of Medical Systems	8
	IEEE European Symposium on Security and Privacy	8
14	Studies in Health Technology and Informatics	7
	Progress in Cardiovascular Diseases	7
	BMJ Global Health	7
15	Journal of Global Health	6
	2018 Latin American Robotic Symposium	6
	Critical Care Medicine	6
	Biomedical Informatics Insights	6
	Radiology	6
	Sustainability	6
	PLoS One	6
	npj Digital Medicine —Nature	6
	2020 International Conference on Sensing, Measurement & Data Analytics in the era of Artificial Intelligence	6
	Others	204
	**Total**	**600**

Table 3. Overview of the academic journals in which experts published

Note: "Others" included 74 journals with 5 experts or less.

We first considered the authors of the 302 publications assessed for eligibility in our scoping review because it covered all key dimensions of the Tool (population health, health system, economic, organizational, and environmental issues). It included 146 peer-reviewed academic articles and 156 gray literature documents (see PRISMA-ScR flow chart in the article). These publications were screened again by RRO, LR, and PL to identify those that aligned with the scope of the e-Delphi exercise. We excluded publications that were highly technical or lacking in substance. We retained a total of 177 publications: 140 academic articles (n=600 experts) and 37 grey literature documents (n=184 experts). Table 3 lists the most frequent academic journals in which these 600 experts' work has been published.

We then searched for and identified the emails of 755 authors over the total of 784 authors. We searched the Internet when e-mails were not available in the publications and included all co-authors. In Round 1, to these 755 experts, we added purposefully sampled experts (n=44) who had been identified through our research collaborators' networks. In Round 2, two experts were recommended by R1 study participants. The aim was to have a balanced representation of the 4 disciplinary fields.

B. 2. e-Delphi Round 1 survey

Section 1 – Introduction to the assessment tool under development

The **aim** of the tool that is under development is to assess the degree of responsibility of digital solutions in health and social care that operate with or without Artificial Intelligence (AI). We use the term "**D/AI solutions**" to refer to both types of solution, which are defined as follows:

- **Digital solution:** an electronic system, including both hardware and software, that can generate, store and/or process data.
  - For example, a virtual reality system can provide patients who suffer from mental health problems with a series of therapeutic sessions where they virtually interact with various avatars, objects, animals and/or environments.
- **AI solution:** an algorithmic system that can infer patterns and draw conclusions from data without explicit programming.
  - For example, an AI solution can be trained to process electrocardiogram (ECG) recordings to detect the presence of cardiac problems and provide a medical interpretation.

What kind of tool are we developing? Who will apply the tool? Who will use its results?

The tool under development is inspired by our work on Responsible Innovation in Health (RIH), which led to the RIH Assessment Tool. The latter is an 8-page supplementary file in: Silva, H.P., Lefebvre, A.-A., Oliveira, R.R., Lehoux, P. (2021). Fostering Responsible Innovation in Health: An evidence-informed assessment tool for innovation stakeholders. *International Journal of Health Policy and Management*. 10(4):181-191.

The preliminary version of the new tool you are kindly asked to appraise follows from a structured review of the scientific and grey literature conducted between December 2020 and May 2021. We **analyzed a corpus of 56 practice-oriented frameworks and tools**, which yielded up to 40 principles used to approach responsibility in D/AI solutions (paper in preparation). We adapted and integrated to the RIH Assessment Tool the principles that were the most conducive to the measurement of the degree of responsibility of D/AI solutions in health and social care.

The tool is structured around the RIH conceptual framework and follows the logic of the RIH Tool. It is a normative tool that will provide a **quantitative measure** of the degree of responsibility of D/AI solutions. The tool entails an evidence-informed assessment process and will be applied by people who possess research skills and are able to search for, retrieve and critically read the scientific literature. It will be freely usable with proper academic citation.

The results generated by the tool are meant to inform the decisions of D/AI solution developers and of those who influence the supply side such as entrepreneurs, investors, research funders, incubators, accelerators, etc. The results will also inform the demand side, including individuals who purchase, prescribe, implement or use D/AI solutions such as patients, clinicians, health and social care managers, etc.

What falls within the scope of the assessment of a D/AI solution's degree of responsibility?

Because D/AI solutions typically rely on a wide-ranging network of digital devices and infrastructures, we set two conditions to determine what components should be included in the assessment: 1) their '*raison d'être*' is to support the D/AI solution; AND 2) they are part of the minimal requirements for the D/AI solution to deliver its service.

- For instance, a portable finger sensor enabling patients to make the ECG recordings that an AI solution uses to detect cardiac problems meets these two criteria:
  - its *raison d'être* lies with the AI solution because it fulfills no other purposes; and
  - it is a minimal requirement because the AI solution cannot detect cardiac problems without it.

- In contrast, the smartphone or tablet the patient uses to access the same ECG-based AI solution falls outside the scope of the assessment because <u>one</u> condition is not met:
    - the *raison d'être* of a smartphone or tablet is not to support this particular AI solution.

Overview of the components of the tool that are appraised in Round 1 of the e-Delphi survey

Figure 1 provides an overview of the tool's key components and highlights which components you are kindly asked to appraise and comment in Round 1 of this e-Delphi survey and which components will be part of Round 2.

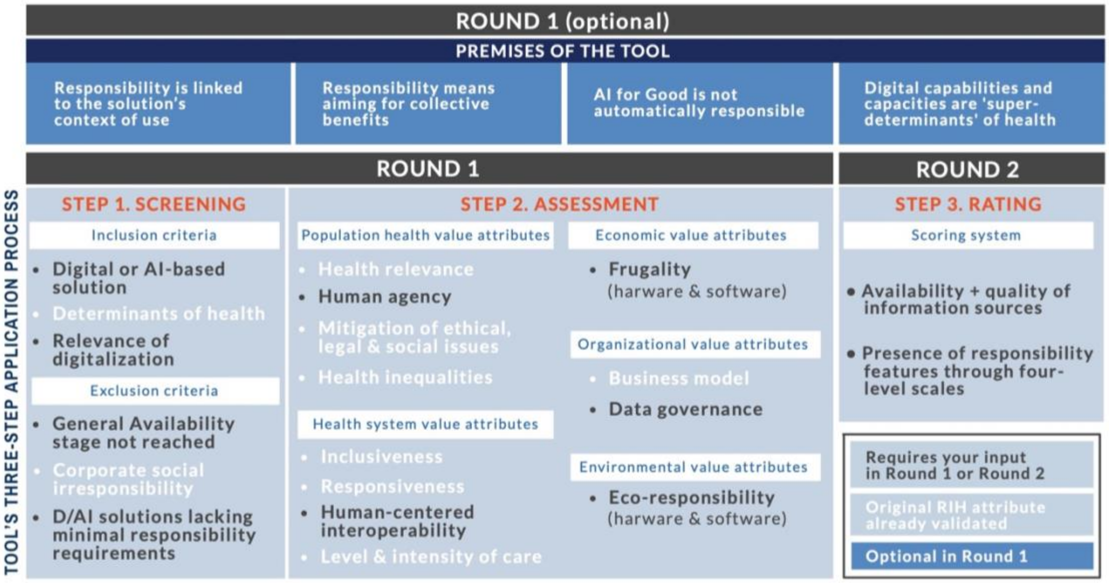

Figure 1. Flow chart of the tool's assessment components

- Located at the top of the figure, the tool's **premises** are meant to clarify how responsibility in D/AI solutions should be approached.
    - Your input on these premises is <u>optional</u> in Round 1.

The **application** of the tool will entail a three-step process:

- The **screening step** aims to determine whether a D/AI solution may potentially qualify as a responsible solution through nine inclusion and exclusion criteria.
    - We <u>need your input on two of these criteria</u> in Round 1.
    - The criteria from the original RIH Assessment Tool that have not undergone any changes do not need to be assessed because their validity and reliability are already established.
- The **assessment step** ascertains the presence of responsibility features through twelve attributes organized into five value domains. All attributes will be assessed through a four-level Likert-like scale, ranging from A to D, where A implies a high degree of responsibility and D implies no particular signs of responsibility.
    - We <u>need your input on five of these attributes</u> in Round 1.
    - We will develop the scales following your input and you will be kindly asked to appraise them in Round 2.
    - The attributes from the original RIH Assessment Tool do not need to be appraised.
- The **rating step** will determine the outcomes of the assessment with the help of a scoring system that takes the availability and the quality of the sources of information used to score each attribute into consideration.
    - The scoring system will be appraised in Round 2.

When completing the e-Delphi survey, please **keep in mind** that the tool should be:

- Consistent with current knowledge

- Easy to understand by people who possess research skills and can critically read scientific literature
- Applicable to a broad spectrum of digital solutions operating with or without AI
- Able to discriminate what is and what is not a responsible D/AI solution
- Concise

Ready to begin Round 1 of the e-Delphi survey? Click here. You may come back to this section at any time.

Section 2 - Participant demographics

This section contains six questions to help us describe the e-Delphi study participants.

Q 2.1 Which of the following groups of disciplines best captures your background and expertise? Select only one.

- ○ Business, public administration or management
- ○ Computer sciences, information sciences or data sciences
- ○ Engineering, design or other natural sciences (e.g., biology, physics, chemistry, geology, etc.)
- ○ Health sciences (e.g., medicine, nursing, rehabilitation, pharmacy, psychology, etc.)
- ○ Law or accounting
- ○ Mathematics, statistics or operational research
- ○ Public health (e.g., epidemiology, global health, health management, evaluation and policy, population health, etc.)
- ○ Social sciences and the humanities (e.g., economics, philosophy, ethics, history, anthropology, linguistics, communication, etc.)
- ○ Other. Please specify [free text]:

Q 2.2 [if above answer is not Health sciences or Public health] **Is most of your work devoted to the health and social care sector?**

- ○ Yes, all or most of it
- ○ No, or very little of it

Q 2.3 Your main affiliation is with what type of organization? Select only one.

- ○ Charity, not-for-profit organization, non-governmental organization (NGO) or multilateral body (e.g., WHO, United Nations)
- ○ For-profit organization, professional consultant firm or privately funded research institution
- ○ Government or arm's length public administration agency (e.g., assessment, regulation, standards and norms, procurement, etc.)
- ○ Higher education (e.g., college, university) or publicly funded research institution
- ○ Health care facility (e.g., hospital, outpatient clinic, community health service, etc.)
- ○ Other. Please specify [free text]:

To describe the diversity of the whole study participant sample, we ask one question pertaining to gender identity, one question about geographical location, and one question about years of experience. These data will be aggregated and will not be used for other purposes.

Q 2.4 To which gender do you identify?

- ○ Gender-diverse (including but not limited to nonbinary, gender-fluid, gender non-conforming)

- I prefer to self-describe as: [free text]
- Woman (including cisgender and transgender woman)
- Man (including cisgender and transgender man)
- I prefer not to disclose

Q 2.5 In which country are you primarily located? [predefined menu]

Q 2.6 Please indicate the year that marks the beginning of your work experience in your field? [predefined menu]

Section 3 - Premises of the tool under development (optional)

This section contains nine questions that are **optional**. You may wish to read the content before proceeding to the next section of the survey.

Recognizing the broader digital sociotechnical ecosystem in which D/AI solutions evolve, four premises clarify how this assessment tool approaches responsibility. You are invited to rate on a five-level scale their **importance** and **clarity**.

3.1 Responsibility is linked to the context of use

The overall responsibility of a given D/AI solution is intimately linked to how and where it is used. While a D/AI solution easily crosses geographic boundaries, it is more difficult for developers to know this context well. Nonetheless, the tool should be applied in view of the social, cultural, economic and political characteristics of the context where the intended users are located.

Q 3.1.1 How important is this premise?

1-Least important 5-Most important

Q 3.1.2 Is this premise clearly defined?

1 - Needs major revision 5 - No revisions needed

Q 3.1.3 If you scored 1 or 2, please explain your rating and/or share ideas for improvement. [free text]:

3.2 Responsibility means aiming for collective benefits

Although a D/AI solution that provides individual health benefits is valuable, a responsible D/AI solution should seek to increase our ability to attend to collective needs and challenges. This may imply, for instance, addressing the root causes of a health or social care problem rather than simply establishing an individual risk level.

Q 3.2.1 How important is this premise?

1-Least important 5-Most important

Q 3.2.2 Is this premise clearly defined?

1 - Needs major revision 5 - No revisions needed

Q 3.2.3 If you scored 1 or 2, please explain your rating and/or share ideas for improvement. [free text]:

3.3 AI for Good is not automatically responsible

Several D/AI solutions are being developed with the explicit intent to ‘do good’, that is, to alleviate social problems and/or contribute to major societal challenges such as the United Nations Sustainable Development Goals (SDGs). Though AI for Good (AI4Good) solutions may generate positive impacts, one should not presume that they are automatically responsible. We thus suggest applying the tool before concluding whether a given AI4Good solution is responsible or not.

Q 3.3.1 How important is this premise?

1-Least important 5-Most important

Q 3.3.2 Is this premise clearly defined?

1 - Needs major revision 5 - No revisions needed

Q 3.3.3 If you scored 1 or 2, please explain your rating and/or share ideas for improvement. [free text]:

3.4 Digital literacies and Internet connectivity are "super-determinants" of health

There is growing evidence that the capabilities to access, use and benefit from digital tools and systems, both at the individual- and group-level, are to be considered "super-determinants of health" because they affect 'upstream' other known determinants of health such as education, housing or employment. Access to the latter increasingly unfold through online transactions, thereby requiring various digital literacies, proper Internet connectivity, an affordable data plan, and low-cost devices that can run recent software releases.

Hence, from a health equity perspective, what is at stake for a D/AI solution in health and social care is not only knowing how to use it, but also having the broader digital capabilities (e.g., skills and competence) and capacities (e.g., means and resources) to materialize its likely benefits. This tool thus recognizes that most D/AI solutions are likely to increase health inequalities unless universal access to bandwidth is achieved and everyone is equipped and supported to become digitally literate.

Q 3.4.1 How important is this premise?

1-Least important 5-Most important

Q 3.4.2 Is this premise clearly defined?

1 - Needs major revision 5 - No revisions needed

Q 3.4.3 If you scored 1 or 2, please explain your rating and/or share ideas for improvement. [free text]:

At this point in the survey, you may wish to go through the other components of the tool and come back to this section later.

3.5 Additional premises

Q.3.5.1 Would you recommend an additional premise?

◯ Yes ◯ No

Q 3.5.2 If you answered YES, please explain what additional premise is needed. [free text]:

Section 4 – The Screening step inclusion criteria

This section contains three questions. You are invited to rate on a five-level scale the **applicability** of two inclusion criteria and suggest additional criteria.

The Screening step relies on three **inclusion criteria** that are meant to swiftly identify solutions that: 1) meet the Digital or AI-based solution definitions; 2) effectively and safely address at least one determinant of health; and 3) explain the relevance of digitalization.

4 Inclusion criteria

4.1 Digital or AI-based solution

An **AI solution** is an algorithmic system that can infer patterns and draw conclusions from data without explicit programming.

A **digital solution** is an electronic system that can generate, store, and/or process data and may include hardware.

Does the solution meet one of the two definitions above?

○ Yes, thus **include**
○ No

Q 4.1.1 How applicable is this inclusion criterion?

1-Least applicable 5-Most applicable

Q 4.1.3 If you scored 1 or 2, please explain your rating and/or share ideas to improve clarity. [free text]:

The following inclusion criteria is shown for your information and does not need to be assessed. It is part of the original RIH Assessment Tool and was already validated.

4.2 Determinants of health

Refer to the factors inside and outside the health system that determine health across one's life course, which include:

- Income and social status
- Social support networks
- Education and literacy
- Employment and working conditions
- Social or physical environments
- Personal health practises and coping skills
- Healthy child development
- Biology and genetic endowment
- Health services
- Gender
- Culture

To qualify as a potentially responsible solution in health and social care, the effectiveness and safety of a D/AI solution must have been demonstrated and it must address at least one of the above determinants of health.

Has the solution been proven effective and safe to human health?

○ Yes, thus **include**
○ No

Does the solution address at least one determinant of health?

○ Yes, thus **include**
○ No

4.3 Relevance of digitalization

Digitalization is a relatively recent technological trend where promises abound. Yet, because of the systemic nature of D/AI solutions and the lucrative industry that has emerged around data exploitation, not all D/AI solutions are relevant in and of themselves (e.g., using face recognition to admit a patient to a health or social care facility) and some may increase the overall burden of care. The decision to turn a non-digital means into a new D/AI solution should thus substantially improve current means of fulfilling such purpose and the relevance of a D/AI solution should be clearly explained.

Is the relevance of the D/AI solution explained in compelling terms?

○ Yes, thus **include**
○ No

Q 4.3.1 How applicable is this inclusion criterion?

1-Least applicable 5-Most applicable

Q 4.3.3 If you scored 1 or 2, please explain your rating and/or share ideas to improve clarity. [free text]:

At this point in the survey, you may wish to go through the other components of the tool and come back to this section later.

4.4 Additional inclusion criteria

Q.4.4.1 Is it necessary to include an additional inclusion criterion?

◯ Yes ◯ No

Q 4.4.2 If you answered YES, please explain what additional criterion is needed and why. [free text]:

Section 5 - The Screening step exclusion criteria

This section contains three questions. You are invited to rate on a five-level scale the **applicability** of two exclusion criteria and suggest additional criteria.

The tool aims to establish a degree of responsibility rather than measure 'irresponsibility.' The Screening step thus relies on three **exclusion criteria** meant to exclude from the assessment solutions that: 1) have not reached the General Availability (GA) stage; 2) are produced by an organization involved in irresponsible corporate actions; or 3) do not meet minimal responsibility requirements in the D/AI industry.

5 Exclusion criteria

5.1 General Availability stage not reached

Because a D/AI solution evolves rapidly, its degree of responsibility can be established more meaningfully at the General Availability (GA) stage. While at the Release to Manufacturing (RTM) stage a D/AI solution is of sufficient quality for mass distribution, GA refers to a point where necessary commercialization activities including security and compliance tests have been completed. When a D/AI solution has not reached the GA stage, we recommend postponing its assessment.

Has the solution reached GA stage in the region where users concerned by this assessment are located?

- Yes
- No, thus **exclude**

Q 5.1.1 How applicable is this exclusion criterion?

1-Least applicable 5-Most applicable

Q 5.1.2 If you scored 1 or 2, please explain your rating and/or share ideas to improve clarity. [free text]:

The following exclusion criteria is part of the original RIH Assessment Tool and does not need to be assessed.

5.2 Corporate Social Irresponsibility

Refers to legal or illegal corporate actions that can harm people, animals or the environment. Examples of such actions by organizations producing D/AI solutions are often linked to the lack of, or ability to circumvent country-specific regulations addressing labor, fiscality and environmental issues and to the difficulty of tracing their electronic suppliers' practices. Harmful corporate actions may be observed in the following domains:

- Animal welfare (physical and psychological, wildlife habitats)
- Community (indigenous or local communities, conflicts associated to rare-earth metals)
- Diversity (women or other underrepresented groups on board of directors or among senior managers)
- Environment (hazardous waste, toxic emissions, harmful mining practices, damages to ecosystems)
- Employees (unions, workers' health and safety, retirement benefits, freelance-based or abusive work conditions of the gig economy)
- Governance (fiscality, tax evasion, managers' compensation, ownership, accountability,)
- Human rights (labour rights, discrimination based on ethnicity, religion, gender or sexual orientation)

- Products (safety, marketing, antitrust, violation of terms of reference, data mismanagement, planned obsolescence, addictive functionalities)

Has the organization that makes the solution available been involved in the past decade or is currently involved in irresponsible corporate actions?

- o No
- o Yes, thus **exclude**

5.2 D/AI solutions that lack minimal responsibility requirements

There are situations where the tool cannot be applied meaningfully because basic minimal requirements for avoiding irresponsible practices in the D/AI industry are not met. D/AI solutions that must be excluded from the assessment may involve four irresponsible practices that are defined as follows:

- **Data reselling is the primary business model:** The main business model of an organization that makes a D/AI solution freely available to users or at low cost can be to generate revenues through data reselling. When a solution primarily exists for generating revenues through a means that is not disclosed to users or intentionally made obscure, it should be excluded from the assessment;
- **The D/AI solution is deliberately deceptive:** A D/AI solution, such as a text- or voice-based chatbot, can appear or be presented to users as if it were a real human interacting with them. When the non-human nature of the solution is not disclosed to users and when there are no reminders of the non-human nature of the solution, it should be excluded from the assessment;
- **There is a lack of cybersecurity and personal data protection:** When an organization that makes available to users a D/AI solution has not established proper measures to guarantee cybersecurity and personal data protection, its solution should be excluded from the assessment;
- **The AI relies on biased datasets:** The dataset used to train an AI solution may be biased, may produce results that cannot be generalized to the entire population of intended users, may lead to unfair decisions against particular individuals or groups or may entice discriminatory behaviours. When the appropriateness of the dataset used to train the algorithmic system has not been properly validated, the solution should be excluded from the assessment.

Does the organization behind the D/AI solution generate revenues primarily through data reselling, deliberately deceive their users, fail to meet established cybersecurity and personal data protection standards, or rely on biased datasets for its AI?

- o No
- o Yes, thus exclude

Q 5.2.1 How applicable is this exclusion criterion?

1-Least applicable 5-Most applicable

Q 5.2.2 If you scored 1 or 2, please explain your rating and/or share ideas to improve clarity. [free text]:

At this point in the survey, you may wish to go through the other components of the tool and come back to this section later.

5.3 Additional exclusion criteria

Q.5.3.1 Do you recommend an additional exclusion criterion?

◯ Yes ◯ No

Q 5.3.2 If you answered YES, please indicate what criterion is necessary and why. [free text]:

Section 6 - The assessment step: Population health value domain

This section contains four questions. You are invited to rate on a five-level scale the **importance** and **clarity** of one responsibility attribute and make suggestions for its **scale** and any other **additional attributes**.

The Assessment step relies on **twelve responsibility attributes** organized into five value domains. Two of the nine original RIH Assessment Tool attributes were substantially modified (Frugality and Eco-responsibility) and three new attributes were introduced (Human agency, Human-centred interoperability and Data governance).

While we only need your input on these five attributes, we show all attributes so you can also look at the four-level scales used in the original RIH Assessment Tool. Based on the input we gather in Round 1 of the e-Delphi survey, we will consolidate the new attributes and develop their scales. You will be kindly asked to appraise them in Round 2.

6 Population health value domain

The Population health value domain relies on four attributes that aim to capture whether the D/AI solution: 1) addresses an important burden of disease; 2) enables humans to exert their autonomy; 3) identifies means to mitigate the ethical, legal and social issues its use may raise; and 4) tackles health inequalities.

The following attribute is part of the original RIH Assessment Tool and does not need to be assessed. Looking at its four-level scale may help you think about the scales that need to be developed for the new attributes.

6.1 Health relevance

Refers to the respective importance of the health needs addressed by the innovation within the overall burden of disease, considering the causes of death, injury and disability and associated risk factors in the region where the intended users are located. Metrics of health relevance include number of deaths, disability-adjusted life years (DALYs), years lived with disabilities (YLDs), years of life lost (YLLs), prevalence and incidence rates. Recent data for such measures (at a global, national or regional level) can be found in the Global Burden of Disease Study of the Institute of Health Metrics and Evaluation.

The D/AI solution addresses a cause of death, injury or disability or a risk factor falling within:

A. The top quarter of all causes of death, injury or disability or risk factors (75% and above)
B. The upper middle quarter (50% to 74%)
C. The lower middle quarter (26% to 49%)
D. The bottom quarter (the lowest 25%)

6.2 Human agency

Refers to the capacity of individuals and groups to exert their autonomy when a given D/AI solution is in use. To enable, encourage and protect human agency, a D/AI solution seeks to empower its users and society more broadly by having full control over the way data about them is being collected and used. Human agency is more likely to be achieved when **procedures** enable individuals and groups:

- To know when a D/AI solution is in use;
- To fully understand a given algorithmic decision or advice;
- To know how to contest such decision or advice;
- To be able to engage in their own preferred course of action, without any undue pressure, personal or professional prejudices;
- To be heard and have one's rights protected when discrepancies arise.

Q 6.2.1 How important is this attribute?

1-Least important 5-Most important

Q 6.2.2 Is the attribute clearly defined?

1 - Needs major revision 5 - No revisions needed

Q 6.2.3 If you scored 1 or 2, please explain your rating and/or share ideas for improvement. [free text]:

Each attribute in the final version of the tool will be accompanied by its corresponding four-level scale, ranging from A to D, where A implies a high degree of responsibility and D implies no particular signs of responsibility.

Q 6.2.4 For the Human agency attribute, what procedures, characteristics or properties should the A on the scale emphasize? [free text]

The following attribute is part of the original RIH Assessment Tool and does not need to be assessed. Looking at its four-level scale may help you think about the scales that need to be developed for the new attributes.

6.3 Means to mitigate Ethical, Legal and Social Issues (ELSIs)

ELSIs refer to a D/AI solution's positive and negative impacts on the moral and sociocultural well-being of individuals and groups and to the legal and regulatory issues its use raises. Although not all ELSIs can be identified at an early stage, a responsible D/AI solution identifies the means by which negative impacts can be mitigated, which may include:

- For ethical issues: User-friendly terms of reference, patient decision-aids, psychological support, group empowerment, practice guidelines, etc.
- For legal and regulatory issues: Laws and regulatory frameworks regarding discrimination (health insurance, the workplace), individual rights, data stewardship (cybersecurity reporting, right to contest algorithmic decisions), privacy, confidentiality, adverse event monitoring, etc.
- For social issues: User support staff knowledgeable of the context where users are located, stigma-reduction programs, caregiver support, community-led educational forums, return to work strategies, etc.

Means to mitigate the negative impacts of the innovation are available for:

A. All three categories of ELSIs
B. Two categories of ELSIs
C. One category of ELSIs
D. None of these categories

The following attribute is part of the original RIH Assessment Tool and does not need to be assessed. Looking at its four-level scale may help you think about the scales that need to be developed for the new attributes.

6.4 Health inequalities

Refers to the avoidable health status differences across individuals and groups that are associated with one's socioeconomic status, social position and capabilities (skills, knowledge, perceived self-efficacy, social network, etc.). Groups who suffer a greater burden of mortality and morbidity due to who they are or where they grow up, live and work are considered vulnerable. Such groups include, but are not limited to:

- Subsistence farmers, long-term unemployed, informally employed, seasonal/daily workers
- People living in deprived urban or rural areas, living in poverty, experiencing homelessness, living with disabilities, living with mental illnesses
- Visible minority groups, asylum seekers, refugees, socially marginalized groups (e.g., lesbian, gay, bisexual, transgender and queer [LGBTQ+], low literacy, etc.).

The D/AI solution:

A. Reduces inequalities by being responsive to the specific capabilities and needs of a vulnerable group
B. May contribute to the reduction of inequalities since ability to benefit from the innovation is not affected by one's socioeconomic status, social position or capabilities
C. May contribute to the increase of inequalities since the ability to benefit from the innovation is affected by one's socioeconomic status, social position or capabilities
D. Increases inequalities by catering to the specific needs of groups whose socioeconomic status, social position or capabilities are amongst the highest

6.5 Additional attributes

Q 6.5.1 Are there additional attributes you think are required to assess the population health value of D/AI solutions?

◯ Yes ◯ No

Q 6.5.2 If you answered YES, please briefly explain the additional attribute(s) you have in mind. [free text; If available, please provide a scientific or grey literature reference]

Section 7 - The assessment step: Health system value domain

This section contains four questions. You are invited to rate on a five-level scale the **importance** and **clarity** of one responsibility attribute and make suggestions for its **scale** and any other **additional attributes**.

7 Health system value domain

The Health system value domain relies on four attributes aiming to capture whether: 1) the innovation design processes were inclusive; 2) the D/AI solution addresses an important system-level challenge in health and social care; 3) the solution is interoperable with existing digital health and social care infrastructures; and 4) the level and intensity of care required by the solution foster health system sustainability.

The following attribute is part of the original RIH Assessment Tool and does not need to be assessed. Looking at its four-level scale may help you think about the scales that need to be developed for the new attributes.

7.1 Inclusiveness

Refers to the degree of stakeholder engagement in the design, development, prototyping and testing of a D/AI solution. Different methods (e.g., codesign, interviews, hackathons, citizen juries, focus groups, workshops, pilot testing, user assessment and feedback) can be used to engage different types of stakeholders (e.g., health and social care practitioners, decision makers, patients, relatives, community and civil society representatives).

Involving a diverse and relevant set of stakeholders through an accountable method is likely to improve a D/AI solution. Hence, RIH makes explicit the rationale and scope of the stakeholder engagement process and its impact on the innovation design and delivery.

Those who developed the D/AI solution:

A. Engaged a diverse and relevant set of stakeholders through a formal method and explained how their input was integrated in the design process
B. Engaged a diverse and relevant set of stakeholders through a formal method, but did not explain how their input was integrated in the design process
C. Either engaged a limited set of stakeholders or did not explain the method used
D. Did not engage stakeholders

The following attribute is part of the original RIH Assessment Tool and does not need to be assessed. Looking at its four-level scale may help you think about the scales that need to be developed for the new attributes.

7.2 Responsiveness

Refers to the ability to provide dynamic solutions to existing and emerging challenges in health systems. To support health system sustainability, a responsible D/AI solution should address system-level challenges, which may include:

- Demographic shifts (ageing, populations affected by climate change, war or conflicts)
- Epidemiologic shifts (chronic diseases, new or re-emerging infectious diseases, orphan diseases)
- Human resources hurdles (training, supervision, turnover)
- Service delivery gaps (accessibility, quality, patient centeredness)

- Knowledge gaps (data analysis and interpretation, development and implementation of knowledge-based tools)
- Governance gaps (coordination, intersectoral action, community partnerships)

The D/AI solution addresses:

A. A system-level challenge that is documented as being of high importance in the target region
B. A system-level challenge that is documented as being of moderate importance in the target region
C. A system-level challenge that is documented as being of low importance in the target region
D. No specific system-level challenges

7.3 Human-centered interoperability

Refers to the ability of the D/AI solution to easily communicate and work with the digital infrastructures already in use in the clinical and non-clinical environments where its users evolve (e.g., the patient's home, community organizations, hospitals, public transit, etc.). A responsible D/AI solution should seamlessly interface with its users' established data management practices, without creating additional cognitive and administrative burden. To achieve human-centered interoperability, a D/AI solution may possess the following **characteristics**:

- Non-proprietary software solutions are used;
- Data sharing functionalities are aligned with the capabilities and needs of different users;
- Users are swiftly informed about the impact of maintenance activities, updates or EOL transition on interoperability;
- Data sharing functionalities robustly 'follow the patient' across non-clinical and clinical environments.

Q 7.3.1 How important is this attribute?

1-Least important 5-Most important

Q 7.3.2 Is the attribute clearly defined?

1 - Needs major revision 5 - No revisions needed

Q 7.3.3 If you scored 1 or 2, please explain your rating and/or share ideas for improvement. [free text]:

Each attribute in the final version of the tool will be accompanied by its corresponding four-level Likert-like scale, ranging from A to D, where A implies a high degree of responsibility and D implies no particular signs of responsibility.

Q 7.3.4 For the Human-centered interoperability attribute, what procedures, characteristics or properties should the A on the scale emphasize? [free text]

The following attribute is part of the original RIH Assessment Tool and does not need to be assessed. Looking at its four-level scale may help you think about the scales that need to be developed for the new attributes.

7.4 Level and intensity of care

Refers to the principle of subsidiarity according to which the most decentralized unit in the health and social care system, including the patient, should be mobilized to provide the service when it is possible to do so effectively and safely. Subsidiarity may be achieved, for instance, by supporting patients' capacity for self-care, enabling proper follow-up by general practitioners, community health and social care providers, or reducing unnecessary interventions at the most specialized level of care of the health system.

While many D/AI solutions may target the patient as the primary user, proper follow-up with formal care providers may still be required (e.g., chronic diseases, mental health, rehabilitation, etc.). To support health system sustainability, a responsible D/AI solution should seek to generate high-quality outcomes while optimizing labour intensity.

The solution was designed to be used safely and effectively mostly under the care of:

A. The patient, an informal caregiver or a health and social care provider operating in a nonclinical environment
B. The patient, an informal caregiver or a health and social care provider operating in a primary health care facility
C. Health and social care providers operating in a secondary or intermediate level of care facility
D. Health and social care providers operating at the most specialized level of care within the health system

7.5 Additional attributes

Q 7.5.1 Are there additional attributes you think are required to assess the health system value of D/AI solutions?

◯ Yes ◯ No

Q 7.5.2 If you answered YES, please briefly explain the additional attribute(s) you have in mind. [free text; If available, please provide a scientific or grey literature reference]

Section 8 - The assessment step: Economic value domain

This section contains four questions. You are invited to rate on a five-level scale the **importance** and **clarity** of one responsibility attribute and make suggestions for its **scale** and any other **additional attributes**.

8 Economic value domain

Frugality

The Economic value domain relies on the concept of frugality which highlights the ability to deliver greater value to more people by using fewer resources such as capital, materials, energy and labour time. Designers of frugal innovation aim to substantially reduce the costs of production, use and maintenance of an innovation, focus on the core functionalities its users require and optimize its performance level considering the intended purpose and context of use.

This attribute will always apply to software and it will apply to hardware when: 1) its *raison d'être* lies with a D/AI solution; and 2) is needed to deliver its service.

- For instance, a portable finger sensor enabling patients to make the ECG recordings that an AI solution uses to detect cardiac problems meets these two criteria: its *raison d'être* lies with the AI solution because it fulfills no other purpose and it is a minimal requirement because the AI solution cannot detect cardiac problems without it. The sensor influences the responsibility of the AI solution because it is a necessary component that would otherwise not exist.
- In contrast, the smartphone or tablet the patient uses to access the same ECG-based AI solution falls outside the scope of the assessment because one condition is not met: the *raison d'être* of a smartphone or tablet is not to support this particular AI solution.

The following attribute is part of the original RIH Assessment Tool and does not need to be assessed. Looking at its four-level scale may help you think about the scales that need to be developed for the new attributes.

8.1 Hardware frugality (when applicable)

The economic value of a D/AI solution may be increased when its hardware incorporates three frugal innovation **characteristics**:

- **Affordability**, which may result from optimized hardware production processes and/or lower maintenance needs;
- **Focus on core functionalities and ease of use** in order to meet the requirements of a larger number of users (e.g., in rural, isolated, remote or resource-constrained settings, etc.);
- **Optimized performance**, which maximizes the fit between the hardware's characteristics and its context of use (e.g., robustness if used in difficult climatic conditions, high autonomy if used in remote settings, economies of scale if used in large centers, etc.).

8.2 Software frugality

The economic value of a D/AI solution may be increased when its software incorporates three frugal innovation **characteristics**:

- **Affordability**, which may result from optimized software development strategy and lower maintenance needs;
- **Focus on core functionalities and ease of use** in order to meet the digital capabilities of a larger number of users (e.g., speaking different languages, with physical and/or cognitive limitations, lacking onsite technical support, etc.);
- **Optimized performance**, which maximizes the fit between the software and the digital capacities in the context of use of the solution (e.g., adapted to settings where connectivity is compromised or data plans are unaffordable, etc.).

Q 8.2.1 How important is this attribute?

1-Least important 5-Most important

Q 8.2.2 Is the attribute clearly defined?

1 - Needs major revision 5 - No revisions needed

Q 8.2.3 If you scored 1 or 2, please explain your rating and/or share ideas for improvement. [free text]:

Each attribute in the final version of the tool will be accompanied by its corresponding four-level Likert-like scale, ranging from A to D, where A implies a high degree of responsibility and D implies no particular signs of responsibility.

Q 8.2.4 For the Software frugality attribute, **what procedures, characteristics or properties should the A on the scale emphasize?** [free text]

8.3 Additional attributes

Q 8.3.1 Are there additional attributes you think are required to assess the economic value of D/AI solutions in health and social care?

◯ Yes ◯ No

Q 8.3.1 If you answered YES, please briefly explain the additional attribute(s) you have in mind. [free text; If available, please provide a scientific or grey literature reference]

Section 9 - The assessment step: Organizational value domain

This section contains four questions. You are invited to rate on a five-level scale the **importance** and **clarity** of one responsibility attribute and make suggestions for its **scale** and any other **additional attributes**.

9 Organizational value domain

The Organizational value domain relies on two attributes aiming to capture the extent to which the organization that produces the D/AI solution has: 1) developed a business model that can provide more value to users, purchasers, and society; and 2) full control over the entire lifecycle of the data its D/AI solution gathers, exploits, generates, archives and/or shares with users and with third parties (voluntarily or not).

The following attribute is part of the original RIH Assessment Tool and does not need to be assessed. Looking at its four-level scale may help you think about the scales that need to be developed for the new attributes.

9.1. Business model

Refers to the components through which an organization creates, delivers and captures social and economic value. A business model typically entails a tension between the redistribution of financial returns to shareholders and the provision of a high-quality D/AI solution. The business model of organizations that seek to provide more value to users, purchasers and society may possess the following **characteristics**:

- Pursue a social and/or environmental mission, operate on a not-for-profit basis or reinvest the majority of the revenues in their mission (e.g., social enterprises);
- Make the solution and its hardware components (see Scope of assessment) freely usable or exploitable by others (i.e., open source, product licensing waivers, do-it-yourself);
- Adopt a pricing scheme based on ability to pay or a redistributive logic (e.g., fees modulated according to user segments);
- Employ people with particular needs (e.g., low literacy, disabilities);
- Comply with social responsibility programs (e.g., Certified B Corporation, SA8000 standard for decent work, ISO26000 for social responsibility).

The business model of the organization that makes the solution available to end users the D/AI solution possesses:

A. Three of the characteristics described or more
B. Two of the characteristics described
C. One of the characteristics described
D. None of the characteristics described

9.2 Data governance

Refers to the stewardship, structures and processes the organization sets in place to ensure full control over the entire lifecycle of the data it gathers, exploits, generates, stores and/or shares with users and third-parties (voluntarily or not). From data collection to data destruction, the organization and its leaders must remain transparent about, accountable for, and swiftly responsive to any breaches in data protection, and to any other issues affecting the D/AI solution data's lifecycle.

Organizations producing responsible D/AI solutions make their high-level executives and employees knowledgeable about and able to report to external auditors on the sensitivity and scope of use of all datasets linked to their solutions. **Procedures** to achieve responsible data governance include:

- Fully active oversight committees whose members' conflicts of interest are publicly declared;
- Explicit compliance to the laws and regulatory frameworks where users are located;
- Adherence to industry standards specific to D/AI solutions (e.g., ISO 13482:2014 for safety of personal care robots, ISO/TS 82304-2 for quality and reliability of health and wellness apps);
- Training programs and certification system for in-house data stewards;
- Fully functional and active reporting systems.

Q 9.2.1 How important is this attribute?

1-Least important 5-Most important

Q 9.2.2 Is the attribute clearly defined?

1 - Needs major revision 5 - No revisions needed

Q 9.2.3 If you scored 1 or 2, please explain your rating and/or share ideas for improvement. [free text]:

Each attribute in the final version of the tool will be accompanied by its corresponding four-level Likert-like scale, ranging from A to D, where A implies a high degree of responsibility and D implies no particular signs of responsibility.

Q 9.2.4 For the Data governance attribute, what procedures, characteristics or properties should the A on the scale emphasize? [free text]

9.3 Additional attributes

Q 9.3.1 Are there additional attributes you think are required to assess the organizational value of D/AI solutions in health and social care?

◯ Yes ◯ No

Q 9.3.2 If you answered YES, please briefly explain the additional attribute(s) you have in mind. [free text; If available, please provide a scientific or grey literature reference]

Section 10 - The assessment step: Environmental value domain

This section contains four questions. You are invited to rate on a five-level scale the **importance** and **clarity** of one responsibility attribute and make suggestions for its **scale** and any other **additional attributes**.

10 Environmental value domain

Eco-responsibility

The Environmental value domain relies on the concept of eco-responsibility which refers to a product, process or method that reduces the negative environmental impacts of a D/AI solution along its lifecycle.

Like the Frugality attribute, eco-responsibility is assessed separately for hardware (applicable when the *raison d'être* of the physical components lies with the solution and are needed to deliver its service), and for programing and software.

The following attribute is part of the original RIH Assessment Tool and does not need to be assessed. Looking at its four-level scale may help you think about the scales that need to be developed for the new attributes.

10.1 Hardware eco-responsibility (when applicable)

The responsibility of a D/AI solution can be increased by attending to eco-responsibility concerns at **key stages in the lifecycle** of its hardware requirements, which include:

- **Raw material sourcing** (e.g., product or hardware made of recycled or renewable content materials, free of substances such as latex, metals or chemicals that are of major public health concern or harmful and toxic to ecosystems)*
- **Manufacturing** (e.g., efficient energy consumption, compliance with national or international environmental regulations, reduced solid or water waste)
- **Distribution** (e.g., packaging, transportation)
- **Use** (e.g., efficient energy consumption, reusability, durability)
- **Disposal** (e.g., product or hardware designed to be recycled, disassembled, remanufactured, composted or biologically degraded)

  * Arsenic, asbestos, benzene, bisphenol A, bromine & chlorine-based compounds, cadmium, chromium, dioxin & dioxin-like substances, lead, mercury, phthalate, PVC.

The solution was designed by integrating hardware eco-responsibility concerns at:

A. Three key lifecycle stages or more
B. Two key lifecycle stages
C. One key lifecycle stage
D. None of the key lifecycle stages

10.2 Programming and software eco-responsibility

Responsibility of a D/AI solution can be increased by using clean energy sources and reducing as much as possible the quantity of energy consumed when training, validating and feeding an algorithmic system or when developing software. Such eco-responsible practices include:

- Choosing programming, modeling or computational techniques that substantially reduce the quantity of energy and time required;
- Using Central Processing Units (CPUs) and computers that are highly energy-efficient (standards);

- Eliminating the use of non-renewable energy sources such as oil, gas and coal;
- Storing and archiving data in data centers and server farms that are net-zero or climate positive.

Q 10.2.1 How important is this attribute?

1-Least important 5-Most important

Q 10.2.2 Is the attribute clearly defined?

1 - Needs major revision 5 - No revisions needed

Q 10.2.3 If you scored 1 or 2, please explain your rating and/or share ideas for improvement. [free text]:

Each attribute in the final version of the tool will be accompanied by its corresponding four-level Likert-like scale, ranging from A to D, where A implies a high degree of responsibility and D implies no particular signs of responsibility.

Q 10.2.4 For the Programming and software eco-responsibility attribute, **what procedures, characteristics or properties should the A on the scale emphasize?** [free text]

10.3 Additional attributes

Q.10.3.1 Are there additional attributes you think are required to assess the environmental value of D/AI solutions in health and social care?

◯ Yes ◯ No

Q 10.3.1 If you answered YES, please briefly explain the additional attribute(s) you have in mind. [free text; If available, please provide a scientific or grey literature reference]

Section 11- General comments about the e-Delphi process

You have reached the final section of the Round 1 e-Delphi survey. You are invited to share any comments you may have about the study and the e-Delphi process.

11.1 Comments to improve the e-Delphi process

Q 11.1.1 In view of Round 2, you may share suggestions to improve the e-Delphi process or any comments about the study [free text]:

B. 3. e-Delphi Round 2 survey

Section 1 – Introduction to the assessment tool under development

Video presentation

In this 10 min. video presentation, we:

- Clarify the kind of tool we are developing;
- Summarise the changes brought to the tool after Round 1;
- Introduce you to the structure of the Round 2 survey.

Aim of the tool

The **aim** of the tool under development is to assess the degree of responsibility of **digital solutions in health and social care that operate with or without AI** (hereafter called "D/AI solutions).

The High-level expert group on AI set up by the European Commission (2019) defines AI systems as software and possibly also hardware systems "that, given a complex goal, act in the physical or digital dimension by perceiving their environment through data acquisition, interpreting the collected structured or unstructured data, reasoning on the knowledge, or processing the information, derived from this data and deciding the best action(s) to take to achieve the given goal."

In the tool under development, the term **D/AI solutions** is used for solutions that operate with or without AI, but it recognizes the specificities of AI, which is concisely defined as:

- An algorithmic system that uses data to infer patterns, draw conclusions and/or make decisions and this process may entail supervised (e.g., machine learning) as well as unsupervised (e.g., deep learning) programming.

For example, an AI solution can be trained to process electrocardiogram (ECG) recordings to detect the presence of cardiac problems and provide a diagnostic interpretation.

What kind of tool are we developing? How do we define responsibility?

The tool is informed by our work on Responsible Innovation in Health (RIH) as well as **a corpus of 56 practice-oriented frameworks and tools** that aim to support responsibility in D/AI solutions. It is structured around the RIH conceptual framework and follows the logic of the RIH Assessment Tool. The tool under development thus aims to provide a **quantitative measure of the degree of responsibility** of D/AI solutions in health and social care.

RIH draws on the policy-oriented field of Responsible Research and Innovation (RRI), which aims to steer innovation towards the 'right' societal impacts. RIH approaches **responsibility as a matter of degree** that can be identified by examining the extent to which an integrated set of process-, product- and organizational-level responsibility attributes are met.

These attributes are not static and thus the RIH Tool establishes whether, at a given point in time, they are present.

The **rationale** of the tool under development is not to measure 'irresponsibility' but rather to account for the extent to which a given D/AI solution brings us closer to achieving the 'right' health and social care impacts. The latter are defined through a health equity lens as well as a health system economic and environmental sustainability lens.

Trade-offs and synergies between responsibility attributes

The original RIH Assessment Tool helps developers identify potential **trade-offs** that can be made between responsibility attributes. For example, in certain situations, it may be legitimate to have a lower score on the Health relevance attribute to reach a higher score on the Eco-responsibility attribute (or vice versa). Conversely, upstream design decisions can be made to specifically increase **synergies** between attributes, thereby augmenting the solution's overall degree of responsibility. For instance, aiming for a higher score on the Inclusiveness attribute can help stakeholders develop a more frugal solution.

Who will apply the tool? At what stage? And to inform what kind of decisions?

The tool was developed to inform the decisions of those who develop D/AI solutions and influence the 'supply side' such as data scientists, programmers, entrepreneurs, investors, research funders, incubators, etc. as well as the decisions of those who influence the 'demand side', including purchasers, implementers, and users of D/AI solutions such as patients, clinicians, health, and social care managers, etc.

Like the original RIH Assessment Tool, this tool will be translated in French and Portuguese, made freely accessible and usable with proper academic citation, and it will be possible to use it in two ways:

1. **As a formal evidence-informed assessment tool**: It will be applied by people who possess research skills. Judgment over each attribute must be made by an interdisciplinary team after having searched, retrieved, and compiled the relevant sources of information. A formal assessment will be more accurate at the General Availability (GA) stage of a D/AI solution because it is a lifecycle juncture where more robust security, usability and compliance tests have been completed and peer-reviewed studies more likely to be published. A formal assessment is thus best performed <u>at the GA stage or after</u>. Repeated application of the tool over time will help track variations in the degree of responsibility of a D/AI solution.
2. **As a structured design or procurement brief**: The tool describes process-, product- and organizational-level responsibility features that can guide the design, development, purchasing, deployment, and use of D/AI solutions. Because the tool's attributes are defined in a tangible way and its four-level scales described in mutually exclusive terms, the tool can be used as a structured roadmap to guide the decisions made either <u>before or after the GA stage</u>.

What falls within the scope of the assessment of a D/AI solution's degree of responsibility?

Because D/AI solutions typically rely on a wide-ranging network of digital devices and infrastructures, we set two conditions to determine what components should be included in the assessment: 1) their '*raison d'être*' is to support the D/AI solution; AND 2) they are part of the minimal requirements for the D/AI solution to deliver its service.

- For instance, a **portable finger sensor** enabling patients to make the ECG recordings that an AI solution uses to detect cardiac problems meets these two criteria:
    - its *raison d'être* lies with the AI solution because it fulfills no other purposes; and
    - it is a minimal requirement because the AI solution cannot detect cardiac problems without it.
- In contrast, the **smartphone or tablet** the patient uses to access the same ECG-based AI solution falls outside the scope of the assessment because <u>one</u> condition is not met:
    - the *raison d'être* of a smartphone or tablet is not to support this particular AI solution.

Overview of the tool's components to be appraised in Round 2

Drawing on all Round 1 comments (n=202), we substantially revised the components of the tool where a robust construct quality threshold was not reached and improved many others. For 85% of the experts or more, additional premises, criteria or attributes were not required. We thus did not introduce new components to the tool. A summary of the results and corresponding changes can be found in Table 1 (hyperlink). Our

responses to the comments are listed in this downloadable document, where you will see insightful criticisms and suggestions.

Figure 1 highlights which components of the tool you are kindly asked to appraise in Round 2 of this e-Delphi survey. The **premises** at the top of the figure clarify the tool's overall approach to responsibility in D/AI solutions.

- You will find the revised premises at the end of the survey and your input is optional again in Round 2.

The **application** of the tool entails a three-step process:

- The **screening step** aims to determine whether a D/AI solution is eligible to a **formal assessment** through five inclusion and exclusion criteria.
  - We revised three criteria and introduced the sources of information that can be used to apply them.
  - We need your input on **two exclusion criteria** in Round 2.
- The **assessment step** ascertains the presence of responsibility features through fourteen attributes organized into five value domains. All attributes are assessed through a four-level Likert-like scale, ranging from A to D, where A implies a high degree of responsibility and D implies no particular signs of responsibility.
  - We revised five attributes and introduced their scales and the sources of information that can be used in the assessment.
  - You are now asked to review **five attributes** and **appraise their scales** in Round 2.
- The **rating step** determines the result of the assessment with the help of a scoring system that considers the availability and the quality of the sources of information used to score each attribute.
  - The scoring system has been already validated and is thus presented for your **information only**.

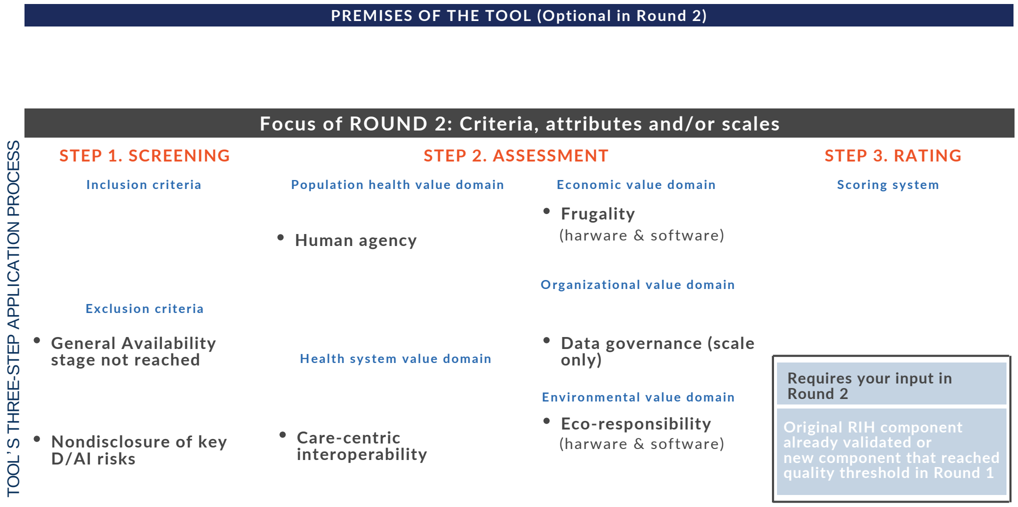

Figure 1. Flow chart of the tool's assessment components

Quality of the sources of information used to rate the criteria and attributes

The types of information source that can be used to assess each criterion and attribute are indicated in the survey. A simple classification for summarizing their quality is used in the scoring system. Because independent organizations and peer-reviewed publications are more likely to be objective in their reporting, they are classified as being of better quality for the tool's assessment purposes.

- **Type 1. Low quality** (1 point): Technical documentation made available by the organization that produces the D/AI solution. This includes the Terms and Conditions statement shared by D/AI solution developers with end users.
- **Type 2. Moderate quality** (2 pts): Reports by multilateral organizations (e.g., WHO, OECD), governments, regulatory agencies, certification bodies or independent not-for-profit organizations that monitor and report on human and labour rights, animal welfare and environmental regulation.
- **Type 3. High quality** (3 pts): Peer-reviewed scientific articles and systematic reviews of the scientific literature (including Health Technology Assessments, Cochrane Reviews, etc.).

Ready to begin Round 2 of the e-Delphi survey? Please click “Participate” to access the consent form. You may come back to this section at any time.

Section 2 – The Screening step inclusion criteria

The Screening step relies on two **inclusion criteria** that are meant to swiftly identify solutions that: 1) meet the D/AI solution definition; and 2) effectively and safely address at least one determinant of health.

The construct quality threshold was reached for the first criterion and the second is part of the original RIH Tool. To see the complete set of criteria and attributes that are part of the RIH Tool, you may download this document.

You may proceed to the next section of the survey.

Section 3 - The Screening step exclusion criteria

The Screening step relies on three **exclusion criteria** meant to identify solutions that: 1) have not reached the General Availability (GA) stage; 2) are produced by an organization involved in irresponsible corporate actions; or 3) do not disclose key D/AI risks to users.

To deliver a valid and meaningful responsibility score when the tool is used in a **formal evaluation process**, the assessment should be made when the D/AI solution has been sufficiently tested, i.e., when the GA stage has been reached. Before this stage, the tool may still be used as a **design or procurement brief** to inform decisions, but we suggest postponing a formal evaluation process. This is the key purpose of the exclusion criteria.

You are invited to rate on a five-level scale the **applicability** of two exclusion criteria that were substantially revised considering all participants’ comments.

3 Exclusion criteria

3.1 General Availability stage not reached

**Criterion definition**	**Question to be answered**
While at the Release to Manufacturing (RTM) stage a D/AI solution is of sufficient quality for mass distribution, General Availability (GA) refers to a point where necessary commercialization activities including security, usability and compliance tests have been completed.<br>When a D/AI solution has not reached the GA stage, this tool may be used to inform design or procurement decisions, but we recommend postponing a formal assessment of its degree of responsibility.	**Has the D/AI solution reached the GA stage in the region where users concerned by this assessment are located?**<br>○ Yes<br>○ No, thus **exclude** from a formal evaluation using this tool<br>**Sources of information required at this stage**<br>• Type 1 info indicating the stage of development of the D/AI solution.

Q 3.1.1 How applicable is this exclusion criterion?

1-Least applicable 5-Most applicable

Q 3.1.2 You may explain your rating or share ideas for improvement, especially if you scored 1 or 2. [free text]:

3.2 Nondisclosure of key D/AI risks

Criterion definition	Question to be answered
Regulation of the D/AI industry is currently scant and unevenly enforced within and across countries. Until proper regulation is implemented and enforced, organizations aiming to produce responsible D/AI solutions in health and social care should refrain from reselling data and publicly disclose their in-house mechanisms to mitigate key risks to users.<br><br>There are at least three **areas of concern** where clear disclosure statements need to be found before applying this tool in a formal evaluation process:<br><br>• **Data reselling**: e.g., an organization can make a D/AI solution freely available to users or at low cost while generating its core revenues by selling user-related data. To avoid any ambiguities regarding its core mission, an organization producing a D/AI solution should refrain from selling data and make its position explicit;<br>• **Cybersecurity and personal data protection**: e.g., cybersecurity and personal data protection require proper high-level governance oversight as well as operational procedures. An organization producing a D/AI solution should describe its data protection measures and disclose how risks are monitored and mitigated;<br>• **AI training datasets**: e.g., the dataset used to train an AI solution may be biased, may produce results that cannot be generalized to the entire population of intended users, may lead to unfair decisions against particular individuals or groups or may entice discriminatory behaviours. An organization producing an AI-based solution should justify the appropriateness of the dataset used to train its algorithm and disclose how potential biases are identified and mitigated.	**Are there public disclosure statements regarding data reselling, cybersecurity and personal data protection, and AI training datasets?**<br><br>○ **Exclude** from a formal evaluation if the answer is 'no' for any of the three applicable areas of concern<br>○ Yes<br><br>**Sources of information required at this stage**<br><br>• Type 1 info indicating whether the organization refrains from data reselling and what risk mitigation strategies it applies for cybersecurity and personal data protection, and AI training datasets.

Q 3.2.1 How applicable is this exclusion criterion?

1-Least applicable 5-Most applicable

Q 3.2.2 You may explain your rating or share ideas for improvement, especially if you scored 1 or 2. [free text]:

Section 4 - The assessment step: Population health value domain

The Assessment step relies on fourteen responsibility attributes organized into five value domains. This first section is for the Population health value domain and contains two questions. You are invited to assess the **clarity** of the Human agency attribute definition and the **appropriateness of its scale**.

4 Population health value domain

The Population health value domain relies on four attributes that aim to capture whether the D/AI solution: 1) addresses an important burden of disease; 2) supports human agency; 3) identifies means to mitigate the ethical, legal, and social issues its use may raise; and 4) tackles health inequalities.

4.1 Human agency (quality threshold reached for importance)

Attribute definition	Scale
Refers to the capacity of individuals and groups to actively and independently exert their decision-making autonomy and act in accordance with their goals when using a D/AI solution.<br>Though D/AI solutions may improve population health by facilitating a range of human decisions and actions, little is known about the way D/AI solutions affect in practice user behaviour, cognition and judgement (e.g., overreliance, avoidance, overconfidence,	**The D/AI solution is accompanied by:**<br><br>A. Procedures that enable users to understand its outputs, decide and act in accordance with their own goals, and include formal means to have their concerns acted upon<br>B. Procedures that enable users to understand its outputs and decide and act in accordance with their own goals, but do not include

hypervigilance) and thus their impact on care seeking behaviours and on health and social care provision.<br>Responsible D/AI solutions can support human agency when there are human oversight **procedures** to enable individuals and groups:<br>• To **understand** a D/AI solution's outputs, that is, the measures, recommendations or decisions it produces (e.g., data visualization and interpretation, decision tree, plain language recommendation, transparency if an AI-based solution is unexplainable);<br>• To discuss these outputs with properly trained staff when needed (e.g., dedicated point of service) and **decide** their own preferred course of action without undue pressure from the D/AI solution itself and from peers (e.g., freedom to use one's judgement or override an AI-based decision, education and training, guidelines);<br>• To have their concerns heard and **acted upon** through formal human oversight mechanisms (e.g., committees for audit, review, appeal, and redress, excluding chatbots, call centers, digital contact forms, etc.).*<br>* The High-level expert group on AI convened by the European Commission (2019) describes three levels of human oversight for AI: 1) human-in-the-loop: human intervention is found in every decision cycle of an AI-based solution (which may often prove neither possible nor desirable); 2) human-on-the-loop: humans intervene in the design of the solution and the monitoring of its operations; and 3) human-in-command: humans oversee the overall use of the solution, can decide whether to use it or not, determine the level of human discretion regarding when and how to use it, and override a decision made by it.	formal means to have their concerns acted upon<br>C. Procedures that enable users either to understand its outputs or decide and act in accordance with their own goals, but do not include formal means to have their concerns acted upon<br>D. No particular human oversight procedures<br>**Information sources**<br>• Type 1 info describing the human oversight procedures accompanying the D/AI solution.<br>• Type 2 or Type 3 info examining the effectiveness of the human oversight procedures accompanying the D/AI solution.

Q 4.1.2 Is the attribute clearly defined?

1 - Needs major revision 5 - No revisions needed

Q 4.1.3 Is the scale appropriate? (i.e., operationalizes the attribute's definition along mutually exclusive levels where A implies a high degree of responsibility and D no particular signs of responsibility)

1 - Needs major revision 5 - No revisions needed

Q 4.1.4 You may explain your rating or share ideas for improvement, especially if you scored 1 or 2 to the questions above. [free text]:

Section 5 - The assessment step: Health system value domain

This section contains three questions. You are invited to assess the **importance** and **clarity** of the Systemic interoperability attribute and the **appropriateness of its scale**.

5 Health system value domain

The Health system value domain relies on four attributes aiming to capture whether: 1) the innovation design processes were inclusive; 2) the D/AI solution addresses an important system-level challenge; 3) supports care-centric interoperability across clinical and non-clinical settings; and 4) the level and intensity of care it requires foster health system sustainability.

5.1 Care-centric interoperability

**Attribute definition**	**Scale**
Refers to how smoothly a D/AI solution can operate within and across the clinical and non-clinical settings where users provide care, manage care, receive care, or take care of themselves (e.g., hospitals, clinics, the patient's home, community organizations) without adding significant cognitive and/or administrative burden to users.	**The D/AI solution:**<br>A. Is periodically adjusted to fit users' digital infrastructures, aligns with their data management practices, and provides all relevant data sharing functionalities

Because the growing use of D/AI solutions may increase fragmentation of care, duplication in data collection processes, vendor lock-ins, or data sharing hurdles across settings (e.g., hospital care units, health and social care system organizations), responsible D/AI solutions are designed to increase their adoptability within the data management practices of their users and adjusted over time to seamlessly communicate and work with their digital infrastructures.<br>Care-centric interoperability can be achieved by:<br>• Designing a solution that is operable on widely available systems and devices and **aligns** with user capabilities, needs, work processes and task allocation to minimize cognitive and administrative burden;<br>• Providing data sharing functionalities that are **well-thought through** administrative processes and clinical pathways (e.g., 'following the patient' across care settings when relevant) and using non-proprietary software or solutions that facilitate data exportation;<br>• Testing the D/AI solution in the context of use before its full deployment and **regularly assessing** how it interfaces with users' evolving digital infrastructures (e.g., robustness, reliability, traceability).	B. Is periodically adjusted to fit users' digital infrastructures and aligns with their data management practices, but provides limited data sharing functionalities<br>C. Either requires substantial adaptations to users' data management practices or provides limited data sharing functionalities<br>D. Requires substantial adaptations to users' data management practices and provides limited data sharing functionalities<br>**Information sources**<br>• Type 1 info describing what makes the D/AI solution interoperable and adoptable.<br>• Type 2 or Type 3 info examining the extent to which the D/AI solution is interoperable and adoptable.

Q 5.1.1 How important is this attribute?

1-Least important 5-Most important

Q 5.1.2 Is the attribute clearly defined?

1 - Needs major revision 5 - No revisions needed

Q 5.1.3 Is the scale appropriate? (i.e., operationalizes the attribute's definition along mutually exclusive levels where A implies a high degree of responsibility and D no particular signs of responsibility)

1 - Needs major revision 5 - No revisions needed

Q 5.1.4 You may explain your rating or share ideas for improvement, especially if you scored 1 or 2 to the questions above. [free text]:

Section 6- The assessment step: Economic value domain

You are invited to assess the **importance** and **clarity** of the Software frugality attribute and the **appropriateness of its scale**.

6 Economic value domain

Frugality

The Economic value domain relies on the concept of frugality which highlights the ability to deliver greater value to more people by using fewer resources such as capital, materials, energy, and labour time. Designers of frugal innovation aim to substantially reduce the costs of production, use and maintenance of an innovation, focus on the core functionalities its users require and optimize its performance level considering the intended purpose and context of use.

Frugality is easily overlooked in the health and social care domain, but it clearly matters to its future. First, most healthcare systems in industrialized countries —be they publicly or privately funded— are struggling with the introduction of increasingly costly and labour-intensive products and services (e.g., gene therapies may cost 2 million US$ per patient per treatment). Second, as shortcomings in globalized supply chains are becoming more acute, there is an undeniable need for using much more wisely the natural and economic resources that go into the production of goods and services. Third, taking heed of frugal design principles will enable D/AI developers to benefit a greater number of patients within and across countries.

The Frugality attribute will always apply to software, and it will apply to hardware when: 1) its *raison d'être* lies with a D/AI solution; and 2) is needed to deliver its service.

- For instance, a **portable finger sensor** enabling patients to make the ECG recordings that an AI solution uses to detect cardiac problems **meets these two criteria**: its *raison d'être* lies with the AI solution because it fulfills no other purpose, and it is a minimal requirement because the AI solution cannot detect cardiac problems without it. The sensor influences the responsibility of the AI solution because it is a necessary component that would otherwise not exist.
- In contrast, the **smartphone or tablet** the patient uses to access the same ECG-based AI solution falls outside the scope of the assessment because **one condition is not met**: the *raison d'être* of a smartphone or tablet is not to support this particular AI solution.

6.1 Software frugality

**Attribute definition**	**Scale**
The economic value of a D/AI solution may be increased when its software incorporates three frugal innovation **characteristics**:<br>• **Affordability**, which may result from optimized software development strategies, open-source programming tools, and/or low technical support, update, and maintenance needs;<br>• **Focus on core functionalities and ease of use** in order to meet the needs and capabilities of a larger number of users (e.g., universal interface design for users with low literacy, physical and/or cognitive limitations, cognitive ergonomics);<br>• **Optimized performance**, which maximizes the fit between software functionalities and requirements and location-dependent digital capacities (e.g., edge-computing for settings where connectivity is compromised or data plans unaffordable).	**The D/AI solution incorporates…**<br>A. All three characteristics of software frugality<br>B. Two characteristics of software frugality<br>C. One characteristic of software frugality<br>D. No characteristics of software frugality<br>**Information sources**<br>• Type 1, Type 2 or Type 3 info describing the D/AI solution's core functionalities, usability and costs, and the resources required for its production, use and maintenance.

Q 6.1.1 How important is this attribute?

1-Least important 5-Most important

Q 6.1.2 Is the attribute clearly defined?

1 - Needs major revision 5 - No revisions needed

Q 6.1.3 Is the scale appropriate? (i.e., operationalizes the attribute's definition along mutually exclusive levels where A implies a high degree of responsibility and D no particular signs of responsibility)

1 - Needs major revision 5 - No revisions needed

Q 6.1.4 You may explain your rating or share ideas for improvement, especially if you scored 1 or 2 to the questions above. [free text]:

Section 7 - The assessment step: Organizational value domain

This section contains one question. You are invited to assess the **appropriateness of the scale** of the Data governance attribute.

7 Organizational value domain

The Organizational value domain relies on two attributes aiming to capture the extent to which the organization that produces the D/AI solution has: 1) developed a business model that can provide more value to users, purchasers, and society; and 2) proper control over the entire lifecycle of the data its D/AI solution gathers, exploits, generates, archives and/or shares with users and third parties (voluntarily or not).

7.1 Data governance (quality threshold reached for importance and clarity)

**Attribute definition**	**Scale**

Refers to the stewardship, structures and processes the organization sets in place to ensure full control over the entire lifecycle of the data it gathers, exploits, generates, stores, and/or shares with users and third parties (voluntarily or not). From data collection to data destruction, the organization and its leaders must remain transparent about, publicly accountable for, and swiftly responsive to any breaches or issues affecting the D/AI solution data's lifecycle.<br>Responsible data governance makes high-level executives and employees knowledgeable about and able to assess and report on the sensitivity and scope of use of all datasets linked to the D/AI solution and this can be achieved through an **integrated set of procedures:**<br>• Defining performance indicators for organizational data protection practices (e.g., certifiable standards such as ISO/IEC 27001 for information security management) and for the D/AI solution (e.g., ISO 13482 for safety of personal care robots, ISO/TS 82304-2 for quality and reliability of health and wellness apps, ISO 42001 for AI);<br>• Securing an ongoing training and certification program for managers and employees to be properly skilled in data management (e.g., data stewards);<br>• Assigning a high-level team, accountable to the board of directors, that monitors the way employees gather, exploit, generate, store, and/or share data and informs users and/or the public of any breaches and incidents;<br>• Integrating the above-described procedures into a reporting system that is auditable by a third party.	**Control over the D/AI solution's data lifecycle relies on:**<br>A. Data governance procedures that include performance indicators and training programs auditable by a third party<br>B. Data governance procedures that include performance indicators and training programs under the responsibility of a high-level team accountable to the board of directors<br>C. Data governance procedures that include either performance indicators or training programs<br>D. None of these procedures<br>**Information sources**<br>• Type 1 info describing the organization's data governance procedures.<br>• Type 2 or Type 3 info examining the quality and outcomes of the organization's data governance procedures.

Q 7.1.3 Is the scale appropriate? (i.e., operationalizes the attribute's definition along mutually exclusive levels where A implies a high degree of responsibility and D no particular signs of responsibility)

1 - Needs major revision 5 - No revisions needed

Q 7.1.4 You may explain your rating or share ideas for improvement, especially if you scored 1 or 2 to the questions above. [free text]:

Section 8 - The assessment step: Environmental value domain

This section contains two questions. You are invited to assess the **importance** of the Programming and software eco-responsibility attribute and the **appropriateness of its scale**.

8 Environmental value domain

Eco-responsibility

The Environmental value domain relies on the concept of eco-responsibility which refers to a product, process or method that reduces the negative environmental impacts of a D/AI solution along its lifecycle.

Like the Frugality attribute, eco-responsibility is assessed separately for hardware (applicable when the *raison d'être* of the physical components lies with the solution and are needed to deliver its service), and for programing and software.

The carbon footprint of current computational infrastructures is close to that of the global airline industry and predicted to double by 2025. Simple hardware modifications can cut in half the energy consumed by software procedures and "coordinated changes in software and hardware could increase the energy efficiency of computing by a million times" (MIT Energy Initiative).

8.1 Programming and software eco-responsibility (quality threshold reached for clarity)

Attribute definition	Scale
Responsibility of a D/AI solution can be increased by using clean energy sources and reducing as much as possible the quantity of energy	**The D/AI solution relies on:**

consumed when training, validating, and feeding an algorithmic system, when archiving data or when developing software. Software design decisions may also affect the quantity of energy used to operate the D/AI solution.<br>Eco-responsible programming and software **practices** may include:<br>• Choosing programming, modeling or computational techniques that substantially reduce the quantity of energy and time required to develop a D/AI solution (e.g., tinyML);<br>• Using Central Processing Units (CPUs) and computers that are highly energy-efficient (e.g., chips and circuits reducing heat transfer);<br>• Storing and archiving data in data centers and server farms where greenhouse gas emissions (GHGs) are reduced to a minimum (net zero) or where more GHSs are removed from the atmosphere than emitted (climate positive) (e.g., ISO/IEC 13273-1:2015 for Energy efficiency and renewable energy sources).	A. Three practices of programming and software eco-responsibility or more<br>B. Two practices of programming and software eco-responsibility<br>C. One practice of programming and software eco-responsibility<br>D. None of the programming and software eco-responsibility practices<br>**Information sources**<br>• Type 1 info describing whether and how the environmental impacts of programming, software development and data processing, storing, and archiving are addressed<br>• Type 2 or Type 3 info analyzing the environmental impacts of programming, software development and data processing, storing, and archiving

Q 8.1.1 How important is this attribute?

1-Least important 5-Most important

Q 8.1.3 Is the scale appropriate? (i.e., operationalizes the attribute's definition along mutually exclusive levels where A implies a high degree of responsibility and D no particular signs of responsibility)

1 - Needs major revision 5 - No revisions needed

Q 8.1.4 You may explain your rating or share ideas for improvement, especially if you scored 1 or 2 to the questions above. [free text]:

Section 9 – Scoring system

This section does not contain any survey questions. It explains the scoring system that is part of the original RIH Tool.

The tool should be applied in a transparent and accountable way. To this end, a **scorecard** to calculate and report the overall responsibility score will be made available as an Excel spreadsheet. Detailed extracts from the sources of information justifying the score given to each attribute should be reported in this scorecard along with a list of references. Because the responsibility attributes of a D/AI solution are not static, the overall responsibility score reflects, at a given point in time, the extent to which an integrated set of process-, product- and organizational-level responsibility attributes are met.

The scoring system relies on **two interrelated components**.

Component 1. Availability and quality of the sources of information

The assessment relies on a sufficient number of attributes when at least 11 of the 14 attributes are documented.

> **Number of attributes documented < 11/14** → The assessment is compromised by missing information
>
> **Number of attributes documented ≥ 11/14** → The assessment covers key aspects of responsible D/AI solutions

The scorecard indicates the sources of information used to score each attribute and the points associated to these sources. If more than one type of source is used for an attribute, the source of highest quality is retained and rated as follows:

- **Type 1. Low quality** (1 point): Technical documentation made available by the organization that produces the D/AI solution.
- **Type 2. Moderate quality** (2 pts): Reports by multilateral organizations, governments, regulatory agencies, certification bodies or independent not-for-profit organizations that monitor and report on human and labour rights, animal welfare and environmental regulation.
- **Type 3. High quality** (3 pts): Peer-reviewed scientific articles and systematic reviews of the scientific literature.

The overall **quality of the sources of information** is determined by calculating the mean value of the points obtained and is interpreted as follows:

Mean score < 2: Low to moderate quality → The assessment is compromised by information sources of inferior quality

Means core ≥ 2: Moderate to high quality → The assessment is based on information sources of superior quality

Component 2. Responsibility features of the D/AI solution

The attributes rely on a four-level Likert-like scale, where:

A = a high degree of responsibility (5 pts)
B = a moderate degree of responsibility (4 pts)
C = a low degree of responsibility (2 pts)
D = no particular signs of responsibility (1 pt)

The **overall responsibility features score** is determined by calculating the mean value of the points obtained, which will fall within one of the following four intervals:

4.1-5.0: Almost all RIH features are present
3.1-4.0: Many RIH features are present
2.1-3.0: Few RIH features are present
1.0-2.0: Almost no RIH features are present

Lastly, to interpret the overall score, one **must consider** whether the assessment relies on: i) a sufficient number of documented attributes (≥9/12); AND ii) information sources of superior quality (≥2).

→ When one of these two requirements is not met, the score is not considered meaningful.

Section 10 - Premises of the tool (optional)

This section of the survey contains six questions and is **optional**.

Drawing on the Responsible Innovation in Health (RIH) framework, four premises clarify how this assessment tool approaches responsibility. They have been revised following the comments shared in Round 1 and you are invited to assess their **importance** and/or **clarity**.

These premises are aligned with the aim of RIH, which is to steer the design and use of D/AI solutions towards the 'right' health and social care impacts. The latter include fostering health equity as well as the economic and environmental sustainability of health systems.

10.1 The context of use largely shapes responsibility (quality threshold reached for importance)

The overall responsibility of a given D/AI solution largely depends upon how and where it is used. The tool should thus be applied in view of the social, cultural, legal, economic, and political characteristics of the context where the users concerned by the assessment are located. Potential shifts in intended use, blind spots in

regulatory frameworks as well as possible shortcomings in public policies in the context of use may affect the overall responsibility of a D/AI solution and thus call for careful attention by those who apply the tool.

Q 10.1.2 Is this premise clearly defined?

1 - Needs major revision 5 - No revisions needed

Q 10.1.3 You may explain your rating or share ideas for improvement, especially if you scored 1 or 2. [free text]:

10.2 Responsible D/AI solutions aim for collective benefits

With the widespread use of smartphones, personal wearable devices and hospital-based devices generating digital data (in radiology, pathology, or cardiology to name just a few), there is a strong tendency among D/AI solution developers to view health needs through an individual perspective (i.e., screening, diagnosing, predicting and/or treating an individual's health problem). As a result, they overlook key opportunities to address through other types of D/AI solutions either the causes of ill-health in large groups of people (e.g., air pollution and cardiovascular diseases) or ways to reduce or eliminate health risks for the collectivity as a whole (e.g., legislation on soft drinks or ultra-processed foods).

An individual perspective also downplays persistent disparities in the distribution of health risks and health benefits across social groups. Those who suffer from ill-health are exposed to health risks that cumulate over their life course, lead to more complex comorbidities and exacerbate the mental and physical "wear and tear of daily life." From a population health perspective, D/AI solutions should be shaped by a thorough understanding of Why are some people healthy and others not?. Those who apply the tool should thus recognize that although a D/AI solution that provides individual health benefits is valuable, a responsible D/AI solution should aim for broader collective benefits.

Q 10.2.1 How important is this premise?

1-Least important 5-Most important

Q 10.2.2 Is this premise clearly defined?

1 - Needs major revision 5 - No revisions needed

Q 10.2.3 You may explain your rating or share ideas for improvement, especially if you scored 1 or 2. [free text]:

10.3 D/AI solutions should tangibly improve current processes and means

Digitalization is a relatively recent technological trend where promises abound and where opportunities around data exploitation have proliferated. Yet not all D/AI solutions are relevant in and of themselves and some may increase the overall cognitive, administrative and/or digital burden for both care providers and care recipients. Responsible digitalization should tangibly improve the digital or non-digital processes and means currently in use in health and social care. Those who apply the tool should thus examine whether the relevance of the D/AI solution is compelling and supported by research.

Q 10.3.1 How important is this premise?

1-Least important 5-Most important

Q 10.3.2 Is this premise clearly defined?

1 - Needs major revision 5 - No revisions needed

Q 10.3.3 You may explain your rating or share ideas for improvement, especially if you scored 1 or 2. [free text]:

10.4 D/AI solutions modulate determinants of health

There is growing evidence that being able to access, use and benefit from digital tools and systems increases health inequalities, both at the individual- and group-level, because it modulates well-known determinants of health. For instance, access to education, housing, or employment increasingly unfolds through online transactions, thereby requiring digital literacy, proper Internet connectivity, an affordable data plan, and low-cost devices that can run recent software releases. Those who apply the tool should not take for granted individuals' wish for using a D/AI solution in health and social care and should look at the broader digital **capabilities** (e.g., skills and competence) and **capacities** (e.g., means and resources) needed to materialize its likely benefits.

Q 10.4.1 How important is this premise?

1-Least important 5-Most important

Q 10.4.2 Is this premise clearly defined?

1 - Needs major revision 5 - No revisions needed

Q 10.4.3 You may explain your rating or share ideas for improvement, especially if you scored 1 or 2. [free text]:

Section 11- Focus of your work, next steps, and general comments about the study

You have reached the final section of the survey. There is one mandatory question left.

11.1 Focus of your work

Q 11.1.1 Is most of your work devoted to the health and social care sector?

Yes, all or most of it No, or very little of it

Next steps

Once the data of Round 2 will be analyzed, we are likely to bring additional minor changes to the tool and then we will assess its **inter-rater reliability** using a diversified sample of D/AI solutions. The final version of the tool, its scorecard and user guide will be sent to you once they are finalized.

Meanwhile, you will receive a report with the results of Round 2.

11.2 Comments about the study

Q 11.2.1 You may share suggestions or comments about the study [free text]:

If you have completed all survey questions, please click here to submit your final responses. Thank you!

B. 4. Dataset: Expert panel comments gathered at Round 1

Premises

**I** = How important is this premise? 1- *Least important; 5 - Most important*; **C** = Is this premise clearly defined? 1- *Needs major revision; 5 - No revisions needed*

**Premise 1 – Responsibility is linked to the context of use (I = threshold reached; C = threshold not reached)**		
**Comment**	**Issue**	**Response**
Although to an insider of the literature on responsible innovation it is clear what you mean by "responsibility", do not forget that this same word is used with a lot of other meanings in the context of AI. Maybe - to increase clarity - you could start by something like "The overall adherence to the principles of responsible innovation of a given D/AI etc." [I=5; C=4]	Wording: Definition of responsibility	1. Thank you. We introduced this suggestion.
The responsible parameters of a D/AI solution should be validated globally and offered to further specify locally (or context dependent) if necessary. [I=5; C=2]	Rationale of the premise	2. We clarified the purpose of this premise.
This premise tried to overcome a flaw the framing of this work, i.e., that a system can be assessed as being responsible or not. This starting point is an oversimplification as being responsible is an ongoing socio-technical act that can _only_ be assessed in a given socio-technical context at a particular point in time. 'Responsibility' is fundamentally _not_ a characteristic that can be measured and that value ascribed to a system or artefact, i.e., the pertinent question is more multifaceted, "who is being responsible to whom about what in which context". Overall, the framework seems to acknowledge and accommodate this view well, but the phrasing of this premise in implying the existence of a quality call 'responsibility' may be misleading. [I=5; C=1]	Rationale of the tool<br>Wording: Definition of responsibility	3. Thank you. We clarified the rationale of the tool and further explained the purpose of the premises.
Digital divide prohibits many people from accessing, using and benefitting from digital devices. [I=4; C=4]	Access to D/AI solutions	4. This is addressed in the premise about determinants of health.
The question of use is central. Probably clarify further what is meant by context: that of individuals, groups/communities, clinics/hospitals, developing country/"developed" country. Another element of context should also cover the "context of the development of the technology" itself. We need to have an idea of the circumstances and context in which the technology was developed, validated and tested. At present, there is a risk of replicating the excesses of the pharmaceutical industry which conducts its trials in countries where regulatory requirements are lighter and less considerate of patients' and individuals' rights. Moreover, it is not just for users to know the context of use. The people who evaluate, purchase and validate them also need to know the context of use. This is not always the case. It also needs to be the same in terms of the development, validation and test contexts. [I=5; C=3]	Clarity: Definition of context of use<br>Context of development	5. We revised the definition.<br>6. We agree. The context of development is underscored in exclusion criteria (Corporate Social Irresponsibility, Minimal requirements) and attributes (Data governance, Business model).
It seems like "context of use" here is limited to the environment. It may be good to clarify the case where a D/AI solution is being used for another purpose than the original intention in the design. For example, same environment but different goal when using the D/AI solution. Then one could call this "responsible usage". [I=4; C=4]	Shifts in intended use	7. We revised the premise to highlight potential shifts in intended use as well as off-label uses.
Pour moi, ce n'est pas clair à quel contexte le "this context" réfère dans la phrase " While a D/AI solution easily crosses geographic boundaries, it is more difficult for developers to know this context well." Si je comprends bien, on parle du fait que les solutions D/AI dépassent les frontières géographiques, et donc, que c'est difficile pour les développeurs de connaître "tous les contextes dans lesquels leurs solutions seraient appliquée"? Si je comprends bien cela, je trouve alors que le "this context" ne capture pas cette	Clarity: Definition of context of use<br>Ambiguity in wording	8. Thank you. We introduced your suggestion ("how and where") in the definition of the premise.

compréhension. Après, la dernière phrase dit "néanmoins, l'outil devrait être appliqué en fonction des caractéristiques du contexte où les utilisateurs cibles se situent. Est-ce que cela veut dire donc que "Même s'il est possible que les solutions D/AI traversent les frontières et que les développeurs ne peuvent pas anticiper et connaître tous les contextes dans lesquels leur solution sera utilisée, iels devraient appliquer l'outil en ayant en tête le contexte dans lequel leurs utilisateurs cible se trouvent"? Donc, cela ne ferait pas vraiment partie de leur responsabilité de se soucier de contextes qu'elleux ne connaissent pas mais dans lesquels la solution D/AI pourrait se propager? Si l'énoncé veut dire ce que j'interprète, je me demande si le modifier comme suit pourrait clarifier: The overall responsibility of a given D/AI solution is intimately linked to how and where it is used. While a D/AI solution easily crosses geographic boundaries, it is difficult for developers to know all possible contexts well. Therefore, the tool should be applied in view of the social, cultural, economic, and political characteristics of the context where the intended users are located. Mais d'un autre côté, si c'est cela que l'énoncé veut dire, je ne suis pas certaine que je suis en accord avec lui. Dans une société mondialisée, je pense que c'est important de se soucier de la propagation des solutions développées au-delà du contexte cible initial. J'ai du mal à répondre donc à l'importance de cette prémisse. [I=?; C=3]		9. The application of the tool requires evidence that properly reflects the context where users are located. This does not imply that the diffusion of D/AI solutions is not of concern.
Though something like the premise above is clearly relevant and important, there is some unclarity in its expression. In the second sentence, for example, it is difficult to know the significance of the sentence clause, "it is more difficult for developers to know this context well." Is this meant only to refer to the possibility that D/AI product developers are unlikely to intimately understand the various contexts in which the products are likely to be used? If so, how might developers approach thinking about the cultural, social, and other factors that would affect how their product is interpreted? [I=4; C=2]	Ambiguity in wording	10. See responses no 3 and no 5.
I would like to see two concepts incorporated into this principle. First, while the principle is not perfect, to apply a phrase, "Perfect is the enemy of the good." It would be a mistake to await perfection because to do so, runs the risk of never realizing the benefits. Second, the impacts of social, cultural, economic and political characteristics can be acknowledged in the guidance given to users of the tool. [I=4; C=4]	Rationale of the tool's premises	11. Thank you. See response no 3.
May be some illustrations of how a given tool can be framed to take into account some of these contextual variations. [I=4; C=3]	Add example	12. A user guide will be developed once the tool is finalized.
This is an important point, but "tool should be applied in view of" still seems rather vague -- is there need of a process before applying it in different contexts, for example, stakeholder engagement etc.? [I=5; C=3]	Stakeholder engagement	13. This is addressed in the Inclusiveness attribute.
While context should be assessed in understanding whether a D/AI solution may be beneficial against the potential risks it could have on the intended users for facial recognition applications as one example, are controversial in most contexts. Just because a technology exists, does not mean it should be used, or it may have limited uses in extreme circumstances. Further, some oppressive regimes may facilitate controversial applications or lack governance/regulation around them, and as such applications should be assessed through human rights and/or patient safety lens as appropriate rather than through political or economic factors (which may be hindrances rather than facilitators for good change). Moreover, many algorithms have been found to be biased and have discriminatory outcomes, in this case it is important that they are trained and developed to ensure they do not harm those subjected to them, which does call for meaningfully understanding the socio-cultural factors where D/AI technologies may be implemented. D/AI Innovations where applied in frugal or developing contexts should be sensitive and designed to the circumstances for example for women's health and hygiene. [I=3; C=3]	Scope of premise: deleterious contexts of use	14. Thank you for raising the issue. We now highlight that risks associated to the context of use are to be carefully considered when applying the tool. See also the exclusion criteria and the ELSIs attribute.
There may be two responsibilities: one located and the other more generic. [I=4; C=2]	Scope of premise	15. See response no 9.

I struggled with this premise: 1. Not clear what is meant by 'overall responsibility'--responsibility of whom? to whom? to do what? This might be obvious to people who work in the field of 'responsible' innovation but less to those outside the discipline. 2. Not clear what is meant by 'the tool should be applied in view of the social, cultural, economic and political characteristics of the context where the intended users are located." Applied by whom? By developers? Users? Both? I also rated this premise only moderately important because there is a risk of cutting ethical and regulatory corners if the context in which the tool will be applied has lax regulations. [I=3; C=2]	Who is responsible to whom<br>Rationale & scope of the tool<br>Unregulated contexts of use	16. See responses no 1 and no 6.
The tool should be applied in view of the social, cultural, etc. : que cela signifie-t-il exactement? ces variables doivent définitivement être prises en considération, mais le comment (et dans quelle mesure) de cette prise en considération est aussi très important. Or, cela n'est pas vraiment précisé ici. Une nuance importante devrait par conséquent être faite pour exprimer que si l'on souhaite prendre en considération les variables sociales, culturelles, etc. dans le design et application de l'outil, on est aussi soucieux de ne pas tomber dans une forme de relativisme culturel, où celles-ci auraient un poids trop grand dans la détermination de l'ensemble de l'évaluation. [I=5; C=3]	Rationale & scope of the premise	17. See responses no 8 and no 9.
Legal context seems important too. [I=4; C=3]	Regulation	18. We agree. This is addressed in the ELSIs attribute.
**Participants who did not complete the Delphi survey**		
The overall responsibility of a given D\AI solution rest primarily on the framing and resolution of the problem set in the D/Ai. Problem solving D/AI is not neutral. When used in a specific cultural context there’s a responsibility to assure the proper development of the users’ capabilities. In other words, there is a double responsibility in the creation and in the uses of D\AI which must be taken care of. [I=5; C=1]	Context of development	19. We agree. See response no 6.
The recent BBC Radio programme entitled Culture on the Couch made me much more aware of the importance of culture in health care, especially mental healthcare. I did not find the concept of geography clear. There are people from many cultures in my legal jurisdiction and my concentric geographic areas (hemisphere, continent, country, province, municipality, city, neighbourhood). [I=4; C=3]	Wording: Definition of context of use	20. Thank you for flagging the issue. See response no 5.
It is very important because social, cultural, economic and political characteristics of the context can either support implementation and access or systemic exclude to groups. [I=5; C=4]	Agreement	21. No specific action required.
I don’t understand this sentence: While a D/AI solution easily crosses geographic boundaries, it is more difficult for developers to know this context well what is meant by "this context". [I=5; C=2]	Wording	22. See response no 5.

**Premise 2 – Responsibility means aiming for collective benefits (I = threshold not reached; C = threshold not reached)**		
**Comment**	**Issue**	**Response**
I scored 3 concerning the "clarity" because the second part of the premise is probably still a bit too broad to allow an accurate assessment as to whether the tool under consideration really meets this criterion. I guess many could argue that even "establishing an individual risk level" could be used (secondarily) to attend collective health needs/challenges. [I=5; C=3]	Clarity: Individual vs. collective benefits	23. We clarified why a population health perspective better supports responsible innovation in health.

If a D/AI solution improves health for some individuals, it could naturally translate into collective benefits since these people will require fewer health resources, leaving more to others. Does that mean that the solution naturally aims for collective benefits? The answer to this question should be clear from the description. Seems like this is linked to responsibility being in the intention vs the result. In my example, the intention could be an individual benefit, but the result is a collective benefit. If you assume that the individual and collective benefits are linked (as in the example), then can we say that the intention is a collective benefit (although the problem is approached from an individual angle)? [I=5; C=4]	Clarity	24. We revised the premise for those who may not be familiar with a population health perspective.
I don't understand this - but it may be because i don't work in healthcare [I=3; C=3]	Clarity	25. See responses no 23 and 24.
The explanation text is fine, but the title is confusing in saying responsibilities _means_ aiming for collective benefits, i.e., it equates the two. Perhaps replace "means" with "includes" or "requires". [I=4; C=2]	Wording	26. We reworded the premise itself.
In personalised and predictive health care it becomes increasingly important that the root cause is attended to, as opposed to merely treating the symptoms. This will be the real value of D/AI used in combination with other personalised and predictive medicines. [I=4; C=4]	Clarity	27. Thank you for the example. See response no 23.
The collective benefit is important. However, there is a small risk that decision-makers and/or users will end up reducing the collective benefit to an "average". This risks leaving some groups/people who will not benefit in a blind spot, even though the technology theoretically has a high average collective benefit. The question of "proportionality" should be integrated. A collective benefit that takes into account the diversity within the target population. [I=5; C=3]	Clarity	28. Thank you for the suggestion. See response no 23.
Somewhat unclear if a product may be responsible even if it does NOT aim at addressing collective benefits. Use of the qualified 'should' leaves room for a product to not do so whilst still being responsible. I think this is probably right but may be worth revising if not an intended implication. [I=3; C=3]	Rationale	29. The value of addressing individual benefits is addressed in the Responsiveness attribute.
Very broad -- might help to have an example for example, is this meaning that data/findings should be able to be accessed for public health purposes? [I=4; C=2]	Clarity	30. See response no 23.
The individual and collective assessment perhaps could be split or this could be a dual premise whereby responsibility is assessed at the individual as well as the collective level. Both are important - what harms may come at the individual level and at the collective level could be unique or related. [I=4; C=3]	Rationale	31. See response no 23.
It is not clear to me whether this principle is saying: (i) in addition to individual health benefits, the tool should try to harvest societal benefits by examining the root causes or (ii) the root causes are more important than individual health benefits and individual health benefits may need to be sacrificed in order to achieve the societal benefits. That is why I have indicated that the premise needs a major revision. [I=2; C=1]	Clarity	32. Thank you for the suggestion. See response no 23.
L'articulation entre "individual health benefits" et "collective needs and challenges" n'est pas claire. Est-ce uniquement le nombre de personnes concernées qui permet d'en déduire qu'il s'agit d'un besoin collectif? [I=2; C=2]	Clarity	33. See response no 23.
The premise is clear but I think any given innovation can be 'responsible' even if it only benefits individuals. [I=2; C=5]	Rationale	34. See responses no 23 and 29.

La dimension collective d'une solution est définitivement capitale à prendre en considération. Toutefois, il doit être rendu plus clair que celle-ci ne prend pas la place des bénéfices individuels, mais plutôt qu'on recherche un équilibre entre le deux. [I=5; C=4]	Clarity	35. See responses no 23 and 29.
It's all context dependent IMHO. In some cases, collective benefits are simply non-relevant. [I=3; C=4]	Rationale	36. See response no 23.
**Participants who did not complete the Delphi survey**		
CLARITY: I do not understand the term "individual risk level" IMPORT: It is not my area of expertise but I am not confident that ignoring differences between individuals is often the best approach to improving health. If I do not understand enough of the reasoning/justification, then I think there is also a greater problem with the clarity. [I=2; C=4]	Clarity: Individual vs. collective benefits	37. See response no 23.
Same as above [it is very important because social, cultural, economic and political characteristics of the context can either support implementation and access or systemic exclude to groups] [I=5; C=4]	Agreement	38. No specific action required.
Does establishing an individual risk level will not indirectly benefit the collective needs? [I=2; C=3]	Clarity	39. See response no 23.
It is unclear what "collective benefits" means. For example, if something work well for the majority of the population but works very poorly for a smaller subset of the population, who are already disadvantaged. Based on this description it is unclear if this would be classified as a "collective benefit"? Who is included i the collective? How is it calculated. Additionally, while I agree with this statement "This may imply, for instance, addressing the root causes of a health or social care problem rather than simply establishing an individual risk level." AI is not designed to identify root causes, so how would this be carried out? [I=4; C=3]	Clarity: Individual vs. collective benefits	40. See response no 23.

**Premise 3 – AI for Good is not automatically responsible (I = threshold not reached; C = threshold not reached)**		
**Comment**	**Issue**	**Response**
In the current formulation, this premise seems a bit "limited" in scope since it simply points at the tool. Maybe you could consider reformulating along these lines, in order to make it more clear and also more relevant (in my view): "Several D/AI solutions are being developed with the explicit intent to 'do good', that is, to alleviate social problems and/or contribute to major societal challenges such as the United Nations Sustainable Development Goals (SDGs). Though AI for Good (AI4Good) solutions may generate positive impacts, simply declaring to adhere to the idea of AI4Good is not enough, but it is necessary for the D/AI solution to be developed transparently, so that it can undergo an assessment of responsibility, such as that offered by this tool". [I=2; C=2]	Clarity: Scope of premise	41. Thank you for the suggestion. We withdrew the premise.
Add a clarification that focusing on the good intentions in one area may lead to failure to notice unintended negative impacts in other areas. [I=5; C=3]	Wording	42. We agree. See response no 41.
It would be interesting to have an example of how/why an AI for Good solution could not be responsible. [I=4; C=4]	Add counter-example	43. Examples will be provided in the tool's user guide.
There is in fact no clear bar for calling something AI4good in the first place, and equally there is certainly no reason why an application that addresses an SDG could also not exhibit other harmful behaviours. So, it seems this premise is stating the obvious while also implying that there is something that can be assessed as being AI4good, whereas this is obviously open to misuse through ethics-washing. It would be better to phrase this premise in terms of how this assessment can be used on applications that _claim_ to be	Rationale & scope of premise	44. We agree. See response no 41.

AI4good or claim to be addressing an SDG goal and makes no assumption about an application based on the label ascribed to it before being assessed. [I=2; C=3]		
Just like human responsibilities, AI must always be held responsible, regardless of its intent. [I=2; C=1]	Agreement	45. No specific action required.
Very important. We should probably add that the question of "good" even raises an epistemological problem about the use of technology. Indeed, who decides that technology is "for good"? This brings me back to the vertical programs in international development. I am quite convinced that we are on this path for AI too, especially with this perspective of AI "for good". [I=5; C=4]	Wording	46. See response no 23.
may be providing a clear definition here of the specificity of responsible. One may make the assumption that if a tool contributes to SDG, it is indeed responsible. Clarify. [I=3; C=2]	Wording	47. See response no 23.
« that's not enough », that's not enough. I suggest specifying what would be enough (the balance of inconvenient I guess?) [I=3; C=2]	Rationale of premise	48. See response no 41.
We need to better define what Ai for good is. [I=3; C=3]	Clarity	49. See response no 23.
A clear and important premise. Stated intentions are often misleading in practice. [I=4; C=4]	Agreement	50. No specific action required.
Rather confusing -- 'responsible' is not quite clear what it means it almost sounds like it's meaning liability in one of the sentences plus not clear who "they" is in terms of responsibility also not clear what the tension is between "doing good" and being responsible within the paragraph - that could be made more explicit. [I=4; C=1]	Link with responsibility	51. We clarified how responsibility is defined and approached in this tool.
It depends how responsible innovation is defined. Also, AI for good may also be good for certain groups but not others e.g., discriminatory algorithms despite their best intentions to promote health and wellbeing. Examples of where AI for good as a concept is defined and has not been responsible would improve the definition here. [I=2; C=2]	Clarity<br>Add counter-example	52. See responses no 23 and 43.
Determining whether a D/AI solution is for good or responsible may have some overlap but cannot be assumed the same. I disagree with the last statement as stated: it implies that you will deploy the D/AI solution in order to assure whether or not it is responsible. That should not be the chosen method to determining responsibility. [I=4; C=3]	Clarify the premise's rationale	53. The aim of the premises is not to "authorize" the deployment of D/AI solutions.
Need more background info on AI4Good and what responsible means here. [I=3; C=2]	Clarity	54. See response no 23.
**Participants who did not complete the Delphi survey**		
All technologies have positive and negative consequences on the users, on the organisations, on society. When we classify a technology as good, we often only consider the Good side of things and forget to analyse the negative impacts on people, organizations or societies. An assessment tool of positive and negative effects is required. [I=5; C=3]	Reflection of the term AI4Good	55. We agree. No specific action required.
CLARITY: I do not understand what is meant by "applying the tool". In my field applying means actual use, not in a sandbox or pilot study. [I=5; C=4]	Clarify the tool's rationale	56. See response no 23.
Exploration of long-term impacts are important before implementation. [I=5; C=4]	The issue is unclear	57. See response no 41.

Why call out one organization? Simply applying the too will not determine if an AI solution is good or not. Additionally, it is unclear why the statement continues to call out AI4Good, specifically in the last sentence where there is no need. This last sentence should refer to all AI tools not just one projects tools. [I=1; C=2]	Clarify the tool's rationale	58. See response no 23.

**Premise 4 – Digital literacies and Internet connectivity are "super-determinants" of health (I = threshold not reached; C = threshold not reached)**		
**Comment**	**Issue**	**Response**
Too complicated and too much information. [I=4; C=2]	Wording	59. We revised the premise, keeping in mind that not all are familiar with public health concepts.
This is a reasonable inter-policy statement to make. [I=1; C=4]	Agreement	60. We do not understand the rating 1 for "importance" in view of the comment.
This point is perfect. In the figure, you had put "Digital capabilities and capacities are 'super determinants' of health", but here you have put "Digital literacies and Internet connectivity are "super-determinants" of health". The terms and concepts should be harmonised. [I=5; C=5]	Update figure	61. Thank you for flagging this discrepancy. The figure was revised.
The concept of "super-determinant "is not easy to understand. I would suggest a more understandable term to describe the concept, such as "baseline condition" or "core element". [I=1; C=3]	Clarity	62. See response no 59.
Important to note that even universal access to broadband and technical literacy will not perfectly protect against inequity. Rather, inequity might become entrenched according to who uses (or does not use) D/AI products. While universal access to these products might help reduce inequity, it could make things worse if primarily the disadvantaged are by circumstance pushed toward using digital health tools while more advantaged persons are able to access conventional health services. As several health systems in Canada increasingly adopt privatized models of care delivery, this might be worth thinking about. [I=4; C=3]	Inequalities<br>Universal access to care	63. Thank you. See the Health inequalities and the Interoperability attributes.
Generally, this seems clearly stated, although seems to be overlooking equity in design in the tools [I=4; C=4]	Equity in design	64. See the Frugality attribute.
I don't like the use of the term "Super-determinants" - it's not defined here and sounds elitist This statement: "that most D/AI solutions are likely to increase health inequalities unless universal access to bandwidth is achieved and everyone is equipped and supported to become digitally literate" ignores whether or not individuals wish to access their health care in this manner. Where is the agency of the user in whether they wish to become digitally literate or equipped? For many people, telemedicine is still new, unfamiliar and undesired. This argument also misses an opportunity to address human-machine interaction in terms of how D/AI solutions might work in conjunction with employees in the health setting. [I=3; C=2]	Wording<br>Human-machine interaction	65. We agree: individuals' wish for accessing and using D/AI solutions cannot be taken for granted (see the Human agency attribute). We revised the premise accordingly. See response no 59.
This principle appears to be more an observation, rather than anything else. Yes, it is conceivable that individual health inequities may be increased and that is an issue that should be examined. However, for individual D/AI solutions, the issues should be around the benefits that the D/AI solution delivers and whether the effort to achieve those benefits is justified in the face of competing priorities, rather than on inequities that arise because some individuals enjoy the benefits of the D/AI Solution while others may not. [I=2; C=3]	Clarify the premises' rationale	66. The current literature strongly supports this premise. See response no 59.

It's mostly speculative for the moment. Many barriers... think of Quebec health system. First step : move out faxes. [I=2; C=2]	Issue unclear	67. We do not understand the comment. See response no 66.
Plutôt clair dans l'ensemble, mais une précision/exemple quant à la façon plus précise dont l'accès aux outils digitaux affecte la santé rendrait le tout encore plus clair et concret. [I=5; C=4]	Add example	68. See response no 59 and no 43.
Like any tool, some expertise is required to interpret the results. Not sure that Digital literacy and internet connectivity are the real barriers here. [I=2; C=4]	Disagreement	69. See response no 59.
**Participants who did not complete the Delphi survey**		
CLARITY OF QUESTION: I would not use importance as labels on a Likert scale for clarity. I think the premise is highly flawed. Even if we assume that we could assess how competent computer users are (and see Alma Whitten's Why Johnny Can't Encrypt for an, old, example of the perennial problem) software and hardware are regularly redesigned to enable more people to use them (although I agree that bandwidth is not often enough a consideration). Furthermore, there are at least a few impoverished regions where cellular technology is used often but such use is often considered a sign of both wealth and sophistication. [I=3; C=5]	Disagreement	70. See responses no 59 and no 66.
Also understanding and adapting it to diverse local cultural context. [I=5; C=4]	Wording	71. See response no 59.
What do you mean by "this tool" in this statement: This tool thus recognizes that most D/AI solutions are likely to increase health inequalities unless universal access to bandwidth is achieved and everyone is equipped and supported to become digitally literate. I think there is a big jump from having access to digital resources is required to benefit from AI to AI (which I think you mean by "this tool") will increase health inequities because not everyone has these things. [I=2; C=2]	Clarify the premises' rationale<br>Disagreement	72. We clarified the purposes of the premises and of the tool.<br>73. See response no 66.

The Screening step

**A =** How applicable is this inclusion/exclusion criterion? 1- Least Applicable; 5- Most Applicable

**Inclusion criterion 1: Digital or AI-based solution (A = threshold reached)**		
**Comment**	**Issue**	**Response**
I would maybe add "draw conclusions...that have the potential to bear on how human decisions are taken or substitute human decisions". Also, i would like to point out that, given the very broad definition of "digital solution" one could argue that "AI solution" is a sub-category included in the broader category of "digital solution" (indeed an AI solution according to your definition would also be a "system that ....process data", and thus it would be also a digital solution). If you think this is the case, then maybe better to make it explicit, otherwise it is redundant to ask, "Does the solution meet one of the two definitions above?", it would suffice to aske "does the solution meet the second definition above?". [A=4]	Clarity : Definition of D/AI solution	74. We revised both definitions following participants' suggestions. We now draw from a well-established reference (High-level expert group on AI, set up by the European Commission, 2019).

The current definition of AI solution does not cover appropriately solutions based on reinforcement learning (an important subfield of AI). I would therefore reformulate: "An AI solution is an algorithmic system that can infer patterns, draw conclusions, and make decisions from data without explicit programming." [A=4]	Precision : Definition of D/AI solution	75. Thank you for the suggestion. See response no 74.
while it is critical to define core terms like AI, this is a fraught endeavour as there are many different competing definitions. It may be better to avoid adding yet another definition and defer to one in use by an authoritative body such as the OECD, EU or ISO/IEC JTC1 SC42 subcommittee on AI. Personally, I think the one defined in the proposed draft of the EU Ai act is good is that it is very broad and cover a range of technologies - potentially even an excel spreadsheet using statistical functions qualifies as an AI system, and if your aim is to avoid harm - why shouldn't it. Your current definition excludes AI that may be based on logical or knowledge based technology (rather than the statistical or machine leaning technologies the wording implies). While these technologies are less in vogue, then can still manifest irresponsible behaviour to they should be included. [A=5]	Clarity	76. Thank you for the suggestion. See response no 74.
This criterion is relevant and relatively easily applicable. However, there are inevitably other uses that will be made of the tool, whether it is digital technology and AI or other. Yes, we could keep this inclusion criterion, but we should keep in mind that the use will vary widely. [A=5]	Intended use vs. real-world use	77. We agree. See response no 7.
In the information section of the study, you gave an illustration on how to think about this inclusion criterion with a finger ECG device and other device - it was helpful for me to quickly grasp the criterion. Without the examples in mind, I don't know if I would have found the criterion as transparent. [A=4]	Use previous example	78. Thank you. We added the examples.
I mean, if the idea is to find digital/AI-based solutions, then sure - just would want to make sure that there's room for recognizing that often the solutions need to be aimed at the human/social layer of how a tool is built/used/implemented. [A=4]	The issue is not clear	79. We cannot address the comment.
For the AI solution, are you speaking about ML solutions or Deep Learning? Maybe not necessary to specify here, but the explanation given could vary. If just ML, for example, I would change the last part of the AI solution statement to : "beyond initial programming", because in ML there is initial programming (supervised) that then the system learns from there. For an AI solution using DL maybe more accurate in lay terms would be "without ongoing programming" (unsupervised). [A=5]	Precision	80. Thank you for the suggestion. See response no 74.
Si la digital solution peut "process data" est-ce que ça inclut de l'IA? Ça ne me semble pas mutuellement exclusif tel que décrit dans les énoncés. [A=3]	Clarity	81. See response no 74.
Bémol: n'étant pas spécialiste d'IA, je peux plus difficilement voir les problèmes avec une telle formulation. Par ailleurs, je préciserais que la solution d'IA va aussi "generate, store, and/or process data". [A=4]	Clarity	82. See response no 74.
Clearer definition of AI is required IMHO. [A=3]	Clarity	83. See response no 74.
**Participants who did not complete the Delphi survey**		
A digital solution includes an AI solution. Why not just talk about digital solutions then? [A=3]	Clarity	84. See response no 74.
I do not understand this question at all. What do you mean by "the solution" And what is this an inclusion criterion for? [A=1]	Clarify screening criteria's purposes	85. We clarified the purposes of the inclusion/exclusion criteria.

Inclusion criterion 3: Relevance of digitalization (A = threshold not reached)		
**Comment**	**Issue**	**Response**
It is not clear the meaning of "such purpose" in the sentence "The decision to turn a non-digital means into a new D/AI solution should thus substantially improve current means of fulfilling such purpose" Also, it is not clear why the last sentence of this criterion repeats the definition of digital solution. [A=3]	Wording	86. We reformulated this criterion into a premise.<br>87. We deleted the example and the misplaced sentence.
Digitalization is too broad to usefully include here - stick to the relevant aspects only for the project. [A=2]	Scope of criterion	88. See response no 86.
Use of 'relevance' may be a bit subjective, perhaps it would be better to talk about the 'relative benefit' of digitization. [A=4]	Relevance of criterion	89. See response no 86.
Je pense que le texte manque de précision ou comporte des partis-pris implicites. Par exemple dans la phrase "The decision to turn a non-digital means into a new D/AI solution should thus substantially improve current means of fulfilling such purpose and the relevance of a D/AI solution should be clearly explained. ", le "such purposes" n'a pas été défini préalablement. Or, c'est crucial pour l'application de ce critère que les lecteur.ices soient d'accord sur ce que sont les "such purposes". Ensuite, dans la question pour inclure ou non la solution, la phrase se limite à "Is the relevance of the D/AI solution explained in compelling terms?", donc on ne parle plus de "such purposes". Est-ce que le parti-pris de ce critère est que les purposes de la solution D/AI soient de "Decrease the burden of care" et que la "relevance" de la solution doit être expliquée en démontrant de quelle manière elle contribue à ce "purpose là"? Je pense donc qu'il faudrait être plus explicite sur ce qui est impliqué par "relevance" (cela découle probablement des prémisses de l'outil). [A=2]	Scope & relevance of criterion	90. See response no 86.
If we are serious with the premise that the context of use is important, then we can't say (and I don't believe) that facial recognition for admission is automatically illegitimate (although I believe this is most likely the case right now, in the present state of technology). [A=1]	Unhelpful example	91. See responses no 86 and 87.
This criterion is not very clear to me. The definition could be more concise and focus on the added value of a digital solution compared to the status quo. [A=3]	Clarity & scope of criterion	92. See response no 86.
A little unclear what is incorporated in "non-digital means" within this context. [A=4]	Wording	93. See response no 86.
The applications must be assessed for their appropriateness for the intended purpose(s) and impact at all levels macro meso and micro. [A=5]	The issue is unclear	94. We cannot address this comment.
This principle is not explained in a clear fashion. I don't understand it. [A=2]	Clarity of criterion	95. See response no 86.
Not sure to understand the phrasing. It's a criteria, a trend, a decision... [A=2]	Wording	96. See response no 86.
The criterion makes sense but the last sentence seems out of place. Also suggest rewording "not all D/AI solutions are relevant in and of themselves"--I am not sure that 'relevant' is the correct word here. The solution could be relevant but not be particularly useful. [A=4]	Scope of criterion	97. See response no 86

Le critère m'apparaît pertinent, mais comment le déterminer me semble plus difficile en termes binaires oui/non. Est-ce qu'une échelle claire déterminant ce qui est pertinent ou non sera établie? Cela me semblerait nécessaire. La dernière phrase ne me semble pas à la bonne place. [A=3]	Applicability of criterion	98. See responses no 86 and 87.

Exclusion criterion 1: General Availability stage not reached (A = threshold not reached)		
**Comment**	**Issue**	**Response**
Although it entails risks certain risks to wait until the GA stage (e.g., the risk that de-implementing a solution/technology after it has come into broad use might be more difficult), i agree that waiting until GA offers the advantage that you evaluate the actual broad operationalisation of the technology. [A=4]	Agreement	99. No specific action required.
Here, there could be an ambiguity if those who use the tool are trying an AI in a "restricted" pilot project context? Should they also reject the technology because it is not at the stage required for routine clinical use? [A=5]	Clarify the purpose of the tool and screening criteria.	100. We clarified why the criterion should be applied when the tool is used in a **formal evaluation**.
I believe this depends on who is using the tool. As a researcher involved in projects where we develop new D/AI solutions that might never reach GA stage, we would find it useful to use the tool to orient our work. Could the recommendation rather be in line with "keeping in mind that degree of responsibility can be established more meaningfully at the General Availability (GA) stage, if the solution has not reached GA stage yet, it should be reassessed at a later time" ? So, I get this would not be possible to use as an inclusion criterion. Je me demande si le fait de le mettre comme un critère d'inclusion/exclusion ferait que l'outil soit mis de côté par des personnes qui auraient pu en bénéficier parce qu'ils ne sont pas encore rendus au stade GA. [A=3]	Clarify the criterion's purpose	101. We clarified how the tool can be applied as a **design and/or procurement brief** at an earlier or later stage.
The concept of GA stage is not necessarily known, so I think that more background information, perhaps a link to a reference, would be useful. [A=3]	Add reference	102. Thank you. We added a hyperlink.
For health tools, can be useful to distinguish between research and clinical availability/implementation. [A=3]	Clarify the criterion's purpose	103. See responses no 100 and 101.
I think the D/AI solution can still be assessed, but in a controlled manner (pilot study) with ethical guidelines. [A=3]	Clarify the criterion's purpose	104. See responses no 100 and 101.
There may be benefits to performing preliminary assessments, before a D/AI Solution reaches the GA stage to identify macro/big picture issues. There should be some criteria that solutions must meet to move to commercialization and these will need to be assessed. [A=3]	Clarify the criterion's purpose	105. See responses no 100 and 101.
It would ideally be possible for developers to flag likely 'responsibility' issues as early as possible and design with these in mind. Could the tool (or a version of it) be designed with this in mind? [A=3]	Clarify the criterion's purpose	106. See responses no 100 and 101.
Même commentaire que précédemment: n'étant pas spécialiste d'IA, il m'est plus difficile de voir le.s problèmes avec le potentiel d'application de ce critère. [A=5]	No issue	107. No specific action required.
Blueprints for security and compliance tests should be enough to assess. Actual implementation is difficult to assess anyway. [A=2]	The issue is unclear	108. We cannot address the comment.

Participant who did not complete the Delphi survey		
Isn't it preferable to assess responsibility and reorient it if needed as early as possible? [A=1]	Clarify the criterion's purpose	109. See responses no 100 and 101.

Exclusion criterion 3: D/AI solutions that lack minimal responsibility requirements (A = threshold not reached)		
**Comment**	**Issue**	**Response**
Very relevant, but you might rethink how to more properly define the last element ("The AI relies on biased datasets"), since many could argue that the vast majority of AI solution are actually (at least currently) developed on partly biased datasets. You could require, for example, as an exclusion criteria for this case that "The AI does not have a strategy/reflexivity element, on how to minimise potential biases in the datasets used for its creation". (or along these lines). [A=4]	Wording: biased datasets	110. Thank you. We revised the set of practices described in this criterion.
Difficult to say right at the start that data protection measures have not been established. How would you use this criteria to rule out some innovation right at the start? [A=1]	Applicability: data protection	111. The information sources needed to apply the criteria are indicated.
Data reselling may not be a sound reason to exclusion - it could be done to help e.g., with new drug research, but if it is done with clear informed consent that may not be a problem. It not reselling that a problem, but it being done without full disclosure. deliberate deception implies knowledge of intent, which can be difficult to determine, whereas if it is just the result of poor design practice then this may be a reason for a poor score and corrective measure, but exclusion may be disproportionate. _relies_ on biased datasets implies we are taking about data somehow central to the design, whereas less central use of biased data, e.g., for testing, could also be interpreted as not reliance, but nevertheless introduce harms. Also, the implied approach places the burden of assessing bias on the assessor, whereas a better approach is to exclude if the approach and method of bias tests are not provided. [A=4]	Relevance: data reselling<br><br>Applicability: biased datasets	112. Outside a clinical research context, data reselling is not regulated. The literature shows that it cannot be part of responsible practices.<br>113. See responses no 110 and 111.
It's hard to define what is "minimally responsible" I'm uncomfortable with "Data reselling is the primary business model". For me, from the moment there is resale of data (whether it is the main or secondary activity), it raises a problem. The exclusion criteria, even the screening part in general, are already evaluation for me. I understand the idea of the screening, but it's kind of an "initial assessment on the 'musts' before moving forward. Secondly, the social irresponsibility and the minimum responsibility part required, overlaps a bit with the "Organizational value attributes" dimension, because it covers, at least partially, the business model (e.g., resale of data). Cybersecurity and personal data protection, and biased datasets refer to the technical assessment of technology. [A=4]	Agreement: data reselling<br>Link with business model attribute	114. We reworded the criterion. See responses no 110 and 111.<br>115. The link with the Business model attribute is not problematic at this stage in the application of the tool.
Je pense que le dernier point est difficilement applicable: The AI relies on biased datasets: The dataset used to train an AI solution may be biased, may produce results that cannot be generalized to the entire population of intended users, may lead to unfair decisions against particular individuals or groups or may entice discriminatory behaviors. When the appropriateness of the dataset used to train the algorithmic system has not been properly validated, the solution should be excluded from the assessment. - car ce qui constitue ou non un "biased" data-set peut être très variable d'un contexte disciplinaire à un autre. Je pense que c'est absolument essentiel d'encourager des datasets non-biaisés, mais je pense que vraiment bien saisir ce qui est ou non un data set biaisé dans différents contexte est difficile. Je ne sais pas si, comme pour le critère où on parle de "relevance" - il faudrait qu'il y ait	Applicability & clarity: biased datasets	116. We agree. See responses no 110 and 111.

une formulation plus du style "absence of compelling argument that the dataset has been developed as to minimize all potential biases". [A=3]		
Some of these practices are very difficult to prove, so it might be difficult to make it an explicit exclusion criteriion. [A=1]	Applicability: unspecified	117. See responses no 110 and 111.
This is perhaps unavoidable, but the final two criteria above strike me as impossible to measure consistently without a more precise rubric. Whether a product lacks cybersecurity or data protection measures, for example, is often not very clear. This isn't necessarily an objective measure, for what counts as a "proper measure" might vary greatly according to context and actual use of a product. It might also be worth considering that data protection measures (as well as the reliance of a particular AI on biased data) can be quite difficult to assess in the abstract. These criteria would benefit from a more thorough description of how they can be measured. [A=2]	Applicability: cybersecurity & biased datasets	118. See responses no 110 and 111.
À l'exception du critère data reselling, il me semble difficile d'avoir accès à des données/informations suffisamment précises pour juger des autres éléments : deliberately deceive their users, // qui juge de ce critère? dans l'absolu ou sur la base de plaintes d'utilisateurs? fail to meet established cybersecurity and personal data protection standards // comment le déterminer, une option serait de vérifier si il suit des protocoles de sécurité reconnus or rely on biased datasets for its AI // comment apprécier le datasets??? [A=1]	Applicability: deceptive, cybersecurity & biased datasets	119. See responses no 110 and 111.
It is not clear from this wording whether the problem is that reselling is the 'primary business model' or that this is not clear to the user. [A=3]	Clarity: data reselling	120. See response no 112.
La formulation du titre "lack minimal responsibility requirements" manque de clarté à deux égards. D'abord, parler de pratiques à risque / problématiques me semblerait plus clair que parler de responsibility requirement - une expression qui n'a pas de signification claire. D'autre part, il faudrait s'assurer de mieux marquer la différence entre le critère d'exclusion précédent et celui-ci, par exemple en précisant qu'il s'agit ici de risques pouvant mener à une conduite irresponsable spécifiquement liés à l'IA et au digital, alors que la question précédente couvrait tous les secteurs d'activité. Par ailleurs, affirmer que la solution sera exclue si "les revenus proviennent principalement de ces activités" semble un critère très flou. Un seuil a-t-il été établi pour déterminer le % de revenus acceptables ou non? Ce calcul peut par ailleurs être difficile à établir, notamment lorsqu'il s'agit de partenariats, filières, projets spécifiques (vs l'ensemble de l'entreprise), etc... [A=3]	Clarity: minimal requirements<br>Distinction with corporate social irresponsibility<br>Applicability: data reselling	121. Thank you for these clarifications. See responses no 110 and 111.
Biases in datasets are difficult to assess, making this criteria difficult to apply. [A=3]	Applicability: biased datasets	122. See responses no 110 and 111.
**Participant who did not complete the Delphi survey**		
It may be hard to evaluate/judge whether a dataset is biased. [A=3]	Applicability: biased datasets	123. See responses no 110 and 111.

The Assessment step

**I =** How important is this attribute? 1- *Least important; 5 - Most important*; **C =** Is this attribute clearly defined? 1- *Needs major revision; 5 - No revisions needed*

Definition of the Human Agency attribute (I = threshold reached; C = threshold not reached)		
**Comment**	**Issue**	**Response**
You may need to rethink the element "To fully understand a given algorithmic decision or advice", since a lot of AI solutions might not meet this, and there is disagreement (at least in ethics) on how "explainability/understandability" is relevant (or even on the definition of these terms). The element "To know how to contest such decision or advice" seems to me the most important. You spoke of digital literacy in the premises. i think that in the future we might need something like "AI literacy" (i.e., learn how to deal with advises, suggestions, recommendations etc. given by AI-solution". Also, you might want to specify whether the elements have to be present alternatively or cumulatively in the assessed AI solution. [I=4; C=3]	Wording<br>Element for scale	124. We agree and deleted “fully” from the definition.<br>125. Thank you for the suggestion. We formulated the scale accordingly.
Important in health because medical licences are only given to humans. [I=5; C=5]	Agreement	126. No specific action required.
The measures mentioned are all ex-post, i.e., once the D/AI application is already deployed. This missed the need to engage with affected stakeholders in the proposal and design of the application, e.g., through participatory design or by giving patient groups whose data is used in training and testing the application a stake in decisions about its deployment and use. On the latter we must remember that these applications are completely reliant on patient data, but once consent is given individuals lose any agency to guide how that data, even once transformed into an AI model, is used. Also, such agency offered to individuals is less powerful than what might be possible if exercised collectively, e.g., via patient groups acting as data unions or data co-ops. [I=4; C=3]	Timing of assessment<br>Stakeholders<br>Element for scale	127. See responses no 110 and 111.<br>128. Stakeholder engagement is in the Inclusiveness attribute.<br>129. Thank you for the suggestion. We formulated the scale accordingly.
I think we should add the "human guarantee" and accountability. Human Guarantee concept is one of the pillars of the European AI development policy among 6 other key principles: (i) technical robustness & safety, (ii) privacy & data governance, (iii) transparency, (iv) diversity, non-discrimination & fairness, (v) societal & environmental well-being, (vi) accountability. https://www.degaullefleurance.com/wp-content/uploads/2020/12/20201130- -DeviceMed- -Article-CTJ-on-Human-Guarantee-in-AI.pdf [I=5; C=4]	Scope of attribute<br>Element for scale	130. Thank you for the reference. We revised the attribute and scale accordingly.
"To be heard and have one’s rights protected when discrepancies arise." - it is not entirely clear to me between what the discrepancies are - discrepancies between the user's perception and the system's recommendation? Also, not entirely clear to me what rights discrepancies would infringe on, and whom would be the key stakeholder in charge of listening. This seems very important to me, but I am wondering how it would be addressed practically. I wonder if this is a point specific to each solution, or if it is a more general point that should be part of policies regulating D/AI at a societal level. [I=5; C=4]	Clarity of attribute<br>Element for scale	131. See responses no 124, 125 and 129.
Agency is not the best term in my opinion . Maybe human autonomy. [I=4; C=3]	Wording	132. See responses no 124, 125 and 129.
This is in my mind an exceptionally important consideration, though I worry that the capacity of a human user to engage in their own preferred course of action will often run up against the tendency of humans to reflexively trust automated processes. That human decision-makers might sometimes fail to scrutinize machines with the degree of care that the situation might demand might warrant consideration here. It might also be worth expanding on the criteria of fully understanding "a given algorithmic decision or advice," particularly through referencing rights to explanation that have been adopted in the European Union, Quebec, and elsewhere. [I=5; C=3]	Scope of attribute<br>Element for scale	133. We agree. See responses no 124, 125 and 129.

"fully understand" seems a steep standard in light of the literature on informed consent and perhaps what should be aimed for more in that is the provision of clear info at a certain grade-level also might acknowledge that there can be some trade-offs involved with agency and obtaining info of scientific value etc. [I=4; C=3]	Wording<br>Scope of attribute	134. See responses no 124, 125 and 129.
All efforts must be made to ensure data subjects etc. are informed and understand the risks and benefits for their acceptability / adoption of technologies. People should be able to change their minds where appropriate and able to if there is individual-facing technologies, though may be more difficult with technologies that are collectively used or clinician-facing. However, mechanisms should be put in place for regular monitoring of preferences of individuals for example through dynamic consent or data access committees/ representative panels. [I=5; C=4]	Dynamic consent	135. See the ELSIs and the Human agency attributes.
This is well stated but the procedures necessary to achieve these objectives will be difficult to implement. For example, enabling an individual to fully understand an algorithmic decision may require disclosure of proprietary information about the algorithm which developers may be reluctant to disclose. [I=4; C=4]	Applicability	136. The scale was developed and clarifies how the attribute can be applied.
Est-il utile de distinguer "fully understand a given algorithmic decision or advice" plutôt que "understand a given algorithmic decision or advice". Peut-on toujours comprendre les choses complètement ? [I=4; C=4]	Wording	137. See response no 124.
Is it realistic to expect users to "fully understand a given algorithmic decision or advice?" This criterion seems like it could be unjustifiably restrictive. [I=5; C=3]	Wording	138. See response no 124.
"by having full control over the way data about them is being collected and used" me semble un peu réducteur (d'ailleurs, l'affirmation suivante couvre beaucoup plus large). À reformuler pour ajouter " a better understanding of how a D/Ai solution work and having sufficient control..." (full control me semble par ailleurs utopique - sufficient est plus réaliste). [I=5; C=4]	Wording	139. See responses no 124, 125 and 129.

**High degree of responsibility for the Human Agency attribute**
As I mention before, the fact that the AI solution comes with a way (whether it's a component or a procedure) to teach how to deal with it (i called it "promoting AI literacy" in the previous comment) [I=4; C=3]
Clear procedures and training of staff for responding to concerns raised by users in a timely and engaging fashion. Notification and access points to enable users to raise concerns. Testing of explanations with contextually appropriate test users to ascertains if the explanations are understandable and useful for the target audience. [I=5; C=4]
This attribute seems in line with decision support systems rather than systems aimed at replacing human experts. From what I understand, a high degree of responsibility for this attribution would be achieved by a D/AI solution that makes a prediction (or recommends an action) using a model that is either transparent (e.g., decision tree) or that can be queried further for understanding its prediction, and that the prediction would be used to guide a human expert in their decision. The human expert could decide to act or not according to the model predictions and that could be logged to further improve the model. [I=5; C=5]
Ability to understand, double-check and invalidate/contest decisions taken by digital tools. [I=5; C=5]
Human in the loop + transparency. [I=5; C=5]
A high degree of responsibility would involve giving the data subjects of health data a real ongoing and non-repudiable say over how their data is used, including a stake in the control of such data. Supporting mechanisms for exercising this control collectively, with strong democratic controls, would also help balance the power between patients overall and those wielding D/AI technology. [I=4; C=3]

Ability for human to access more information if he or she so wishes to consider over a period of time with which the patient is comfortable to make a free decision in line with his or her values and beliefs. [I=5; C=5]
Technology makes it possible to leave the final choice (the freedom to do and act) to the human. That it does not in any way challenge the possibility of human action (e.g., patient, professional) [I=5; C=4]
I have very limited time to fulfill the study - answering this question would require more reflection time that I have. I am sorry to participate in this limited way but I hope the answers I provide, even if incomplete, will still be of some use to your team. [I=5; C=4]
If using this solution improves user agency, for example a watch that monitor and let you know your own Physical Activities, that improves your agency. The same watch that centralize the data and force adoptons other proprietary solutions, that decrease your agency. [I=3; C=1]
I think both procedures and property are important and need to be designed in such a way to assure the attribute of human agency. [I=5; C=4]
Involvement of end-users in the development of the digital solution and/or algorithm. [I=5; C=5]
Highly responsible AI would generally be reviewable, though it is important to note that certain systematically inscrutable products, such as unexplainable deep learning might not be susceptible to careful review. In these instances, it would not be responsible for developers to give users the impression that the AI product can be reviewed or explained if this is not so. It might in some instances be MORE responsible to signal a product's unexplainable character than to falsely or misleadingly suggest that review is possible. Use of unexplainable products may be responsible insofar as the product in question satisfies other criteria listed in this section, such as knowing when a product is in use. Attentive balancing of these factors would be important. [I=5; C=3]
Procedure for allowing agency Design that supports agency (such as indicators of when data is being collected and so forth). [I=4; C=3]
To fully understand a given algorithmic decision or advice this needs more work by both solution providers and organizations implementing them. [I=5; C=5]
I don't see the relation between "Procedure, component and property" and responsibility. [I=4; C=3]
Je ne suis pas certaine de comprendre la question. Selon ce que j'en comprends, je dirais: fournir une explication en amont de l'utilisation par une personne humain, en face-à-face avec l'utilisateur, et en aval, mettre en place une boucle de rétroaction pour réajuster en fonction des expériences et problèmes réels. [I=5; C=4]
**Participant who did not complete the Delphi survey**
When procedures allow these 5 items. [I=5; C=5]

**Definition of the Human-centered Interoperability attribute (I = threshold not reached; C = threshold not reached)**		
**Comment**	**Issue**	**Response**
I think an additional characteristic might have to be added, which could somehow ensure that the worry you rightly express (i.e., that the D/AI solution operates "without creating additional cognitive and administrative burden") does not materialise. E.g., a D/AI solution which allows a decision to be taken more quickly, but which requires much more time for inputting the data etc. This characteristic could be something like "the D/AI solution does not require additional chores to the patient and/or the healthcare (or social) worker, which would negatively disrupt the provision of care." [I=4; C=4]	Precision: additional chores	140. Thank you for the suggestion. We revised the attribute accordingly.
It should show how the human in the loop is operating. [I=5; C=4]	Human-in-the-loop	141. Thank you. It is now part of the scale.

This attribute seems to conflate interoperability, which yields benefits in terms of lifetime costs of systems (including procurement, testing, and QA costs) and the quality of user friendliness, which also confuses system user and data subjects (often not the same, e.g., patients vs clinical professionals). [I=4; C=1]	Scope of attribute	142. We aim to work with a limited number of attributes. We revised the definition to increase clarity.
In my opinion, this attribute could be subdivided into two. There is human-centred interoperability. But there is also the question of ergonomics and usability, which refers among other things to the "human-machine" interface. You can put them together in one attribute, but it could be two attributes as well. [I=4; C=4]	Scope of attribute<br>Usability	143. See response no 142.<br>144. Usability is addressed in the Frugality attribute.
The clause concerning maintenance activities could be stated somewhat more clearly. What interoperability impacts does this line contemplate? [I=3; C=4]	Wording	145. See responses no 140 and 142.
Generally, yes, although practically speaking, interoperability has been a goal for a long time in a number of informatics/data areas and has been elusive. [I=3; C=4]	Applicability	146. The scale clarifies how the attribute can be applied.
Not sure who you are referring to as the humans in the system: the patient-facing users in health care settings? or the end-users, i.e., patients. [I=4; C=3]	Clarity of attribute	147. See responses no 140 and 141.
One of the criteria is that "non-proprietary software solutions are used". That seems to me to incorrectly state the issue. I view the question as whether there are mechanisms to allow an individual to understand the decision that was reached and how it was reached. There are alternative ways of achieving this result than allowing only non-proprietary software solutions. The criterion should be whether these alternative ways deliver the desired result, rather than whether a particular mechanism is used. [I=3; C=3]	Disagreement: non-proprietary software	148. The literature indicates that proprietary software solutions create major inter-operability hurdles in health and social care systems.
Le principe d'interopérabilité est louable. Toutefois, il ne faudrait pas que des organisations très en retard en termes technologiques (celles avec des fax notamment ...) ne fassent pas de mises à jour de leur système. Sinon, il y a un risque de retarder le développement technologique en raison d'un retard chronique dans certaines organisations. De plus en indiquant : without creating additional cognitive and administrative burden. Ça sous-entend qu'il ne peut y avoir aucun impact avec la technologie. Il y en a toujours (même dans le cas d'une mise à jour d'une tablette ou d'un système d'exploitation). Par contre, ils ne sont pas tout le temps importants. Il faudrait nuancer cet élément pour que le coût soit raisonnable en fonction des avantages de la technologie. [I=1; C=4]	Wording (adding nuances)	149. See response no 140.
Not sure what you mean by "where its users evolve". [I=5; C=4]	Wording	150. See response no 140.
Même commentaire que précédemment: n'étant pas spécialiste de l'IA ou des technologies digitales, je peux difficilement voir les problèmes avec la formulation exacte de cet attribut. [I=5; C=5]	No issue	151. No specific action required.
What is described is mostly technical interoperability. [I=4; C=3]	Wording	152. See response no 140 and 142.

**High degree of responsibility for the <u>Human-centered Interoperability</u> attribute**
That it is evaluated by the patient/care-receiver and the (health)care provider as actually making the provision of care more seamless and to increase the (quality of) time dedicated to the care-receiver. [I=4; C=4]

A D/AI solution with a high degree of responsibility for this attribute would be available on all platforms (e.g., Windows, OSX, and Linux) and users would be able to export their data in a general, non-proprietary, format (e.g., CSV file). [I=5; C=5]
Very important criteria. This is one aspect that is not that explicit in the definition and explanation of the criteria: the digital or AI solutions need to be interoperable with existing information systems within an organizational context. This not only about the human component that is important but also the technical one. [I=5; C=5]
A high degree of responsibility would be characterised by having a clear, continuous register of the overall health system into which the D/AI application is integrating and the stakeholder goals which are addressed by those system goals so that gaps in stakeholder agency and participation can be identified, filled and monitored over time as more systems are added and integrated. [I=4; C=1]
A technology with fewer connection and adjustment steps with the user's technologies and infrastructure. It should also be as intuitive as possible. In other words, the technology should be developed with a "universal precaution" "approach to health literacy, in which organizations design communications strategies with the assumption that any patient may need literacy support, rather than seeking to identify subsets of low-literacy patients for special attention. Universal-precautions measures include writing actionable content,114 using plain language, using visuals such as pictographs, and minimizing text-based input." Link : https://academic.oup.com/jamia/article/25/8/1080/4996916. [I=4; C=4]
I don't see how to operationalize this idea. [I=2; C=2]
The presence of all aspects (Non-proprietary software solutions are used Data sharing functionalities are aligned with the capabilities and needs of different users; Users are swiftly informed about the impact of maintenance activities, updates or EOL transition on interoperability Data sharing functionalities robustly 'follow the patient' across non-clinical and clinical environments.) would represent a high degree of responsibility. [I=4; C=5]
Capacity and context alignment strike me as the most important factor in this section for assessing responsibility. [I=3; C=4]
Approachability, comfort for the users and demystifying the technology. [I=4; C=3]

**Definition of the Software Frugality attribute (I = threshold not reached; C = threshold not reached)**		
**Comment**	**Issue**	**Response**
I was expecting to see here something about software developed using open-source tools/languages, even a mention of open-source software. Maybe it lies within "Affordability"? In any case, I would add something about open-source software to clarify whether or not it influences this attribute. [I=5; C=3]	Scope of attribute	153. Thank you. Open source is part of the Business model attribute.
Too much focus on cost will be counterproductive - the issue is more about how this is funded. [I=3; C=4]	Cost vs. sources of funding	154. The literature indicates that a solution that was deliberately designed to reduce its cost is more valuable from a purchaser and/or end user perspective.
while the attribute mentions frugality, it need to acknowledge that the properties mentioned may conflict with each other and therefore trade-offs need to be made if the budget is an overriding issue. Also, this ignores the major cost issue of the compliance with medical device and safety requirements and regulations. [I=4; C=2]	Trade-offs b/w properties<br>Cost of regulation	155. We agree. The tool takes these trade-offs into account.
This should be better defined. I don't understand it fully and don't see the link with performance Optimized performance, which maximizes the fit between the software and the digital capacities in the context of use of the solution (e.g., adapted to settings where connectivity is compromised or data plans are unaffordable, etc.). [I=3; C=3]	Clarity	156. Thank you. We revised the attribute definition.

Perhaps affordability should be challenged by introducing the concept of "open source" technology. That would democratize the access to the D/AI solution software. [I=3; C=3]	Wording	157. See response no 153.
Me semble une question très (trop) chargée et par conséquent très complexe à évaluer adéquatement. [I=4; C=2]	Clarity & applicability	158. See response no 156.
Maybe refer to existing tools: https://digitalprinciples.org/ [I=3; C=4]	Existing tools	159. Thank you.

**High degree of responsibility for the Software Frugality attribute**
Procedures for testing the D/AI on the systems and infrastructure that is available in the use context. [I=5; C=5]
A software that anyone (in the targeted context) can easily use and access in its most recent version. [I=5; C=3]
I really like this criteria. It is much connected with the notion of accessibility. Make it cheap, yet with enough functionalities to make it accessible and usable by multiple individuals. [I=5; C=5]
These technologies are now mainly under "Software as Services" models. I don't know if this is relevant, but frugality should also cover the "service" dimension that accompanies these technologies. Services should be accessible, and value user empowerment (e.g., right to repair, bricolage if necessary). This dimension also refers to the "supply chain". But this has not yet been mentioned. [I=4; C=4]
Physical units per user. [I=3; C=4]
The presence of all 3 elements would constitute a high degree of responsibility. [I=5; C=5]
Products that achieve a reasonable cost-benefit balance would tend to be more responsible than those that do not. This might be an especially relevant consideration when products are used in the context of a public health system. [I=4; C=4]
In developed countries software/hardware frugality is not that important. [I=3; C=3]
Procedure - both in terms of mechanisms for payment/responsibility, and also assessing. [I=4; C=5]
See above [Perhaps affordability should be challenged by introducing the concept of "open source" technology. That would democratize the access to the D/AI solution software.]. [I=3; C=3]

**Definition of the Data Governance attribute (I = threshold reached; C = threshold reached)**		
**Comment**	**Issue**	**Response**
"Explicit compliance to the laws" is a bit of a broad element. How would this be certified? there are mechanisms (developed or in development) to certify this - especially in the healthcare domain - but they also include a risk that legal compliance becomes box ticking. So, if you want to keep this, maybe you could be more specific as to how this should be achieved in this context, and on its added value. Also, the element of "training programs" is a bit broad: what kind of training programs for good data stewardship? From a technical point of view? or legal? or ethical? [I=4; C=3]	Scope of attribute	160. Thank you for raising the issue. We revised the attribute and developed a scale that clarifies how it applies.
What about data management by users? For example, having a clear and understandable way for users to opt out of data sharing? Should that be part of data governance? [I=5; C=4]	User data management	161. We integrated this point in the attribute definition.

You introduce reference to safety and quality standards, which is positive, but these go beyond the scope of data governance and would be applicable to other attributes also. Also, there are data governance standards you could also be referencing. [I=4; C=2]	Add standards	162. We now refer to these standards.
It is not clear to me what "explicit compliance to the laws and regulatory frameworks where users are located" might tangibly mean in this context. One of the significant characteristics of digital health and AI is that the rules surrounding their use and design are often unclear or unsettled. There is significant uncertainty in Quebec and Canada, for example, about what precisely the law expects of D/AI developers, as well as D/AI users. As a criterion for measuring responsibility, therefore, legal and regulatory compliance may assume that the normative landscape at present is more developed than it is in reality. [I=5; C=2]	Gaps in regulation	163. We agree and have revised the exclusion criteria as well as the premises. The tool can help 'demand side' stakeholders to identify these potential gaps.
Element of expertise/training for members of committee. [I=5; C=4]	Element for scale	164. Thank you. It is in the scale.
The org and leaders need to actively work to prevent issues as well. So just like in health care, data stewardship should refer to an active stance of prevention (of breaches/cyber-attacks, misuse of data, leaks, etc.) So strengthening and prioritizing cybersecurity. Also, selection of in-house data stewards and the training should seek diversity of perspectives and be transparent and responsible in the process. Compliance with the organization's company policies, including employee codes of conduct, vendor agreements, ethics guidelines, etc. [I=5; C=3]	Scope of attribute	165. See response no 160.
Bonifier la formulation de la 1ere procédure en précisant que l'on cherche d'abord et avant tout à éviter les conflits d'intérêts, et si ce n'est pas possible, à les gérer adéquatement: "Fully active oversight committees whose members are not in conflict of interests. When conflicts of interests are present or not avoidable, they have to be publicly declared and adequately managed". [I=5; C=4]	Scope of attribute	166. See response no 160.

**High degree of responsibility for the Data Governance attribute**
The presence of certification systems on data handling, and also a certain investment (e.g., in terms of training programs) in 'human-ware' (i.e., on the skills in terms of responsible data governance of those that actually handle data). Finally, also the element of performing a data protection impact assessment (on the line of what is required - at least in Europe - by the GDPR) could be considered. Also, in terms of data infrastructure, an important element could be transparency on the origin of the infrastructural elements on which the D/AI solution is based (e.g., does it rely on infrastructure provided by the big companies active in the data field? if so, for what reasons?) [I=4; C=3]
Data governance should also include a clear strategy, with the necessary means and expertise, to ensure also a "risk governance" of what could happen with AI. The technology provider needs to have clear procedures and processes on what will be done in likely scenarios, but also in case of unintended incidents. An important point missing is the importance of transparency and communication in case of data incidents for example. Patients and other users have a right to know and be informed about what happens to their data, how it is used, and especially in case of incidents. We have seen how Desjardins struggled to "manage" and communicate with its customers in a transparent manner during the data incident. [I=5; C=4]
Supervision from institutional ethical committee. [I=2; C=3]
The four first elements seem very important. For the "Fully functional and active reporting systems", I'm not sure how this is relevant for data governance. [I=5; C=5]

Definition of the Programming and software eco-responsibility attribute (I = threshold not reached; C = threshold reached)		
**Comment**	**Issue**	**Response**
Unclear how this can easily (and exactly) be evaluated. [I=4; C=5]	Applicability	167. Participants are now invited to assess the scale that clarifies how this attribute applies. We also added the type of information that should be made available by D/AI solution developers.
While these are good goals, they need to be considered in relation to trade-off against other goals, e.g., going into standby mode more frequently may reduce responsiveness or availability. Also, it may require an assessment trade-off between capital cost and operational cost. [I=2; C=3]	Trade-offs<br>Costs	168. We agree. The tool enables one to document and assess these trade-offs. Economic issues are addressed in the Frugality attribute.
It seems that there is little attention to eco-friendliness in the step when the system is actually used. [I=4; C=2]	Environmental impacts of use	169. Thank you. We revised the attribute and scale accordingly.
I'm not sure most people understand the particularities that go into determining whether or not a D/AI solution is ecologically responsible. It would be good to explain or train on this. [I=2; C=3]	Awareness of D/AI solutions' footprint	170. We agree and this is why it should be part of the tool.
I think this criteria must be balanced against the benefits that the D/AI Solution delivers and that balancing is not reflected in this principle. [I=3; C=3]	Rationale of the tool	171. Thank you. See response no 167.
Considérer ajouter la minimisation des données utilisées (ce qui contribuerait par ailleurs à renforcer le respect de la vie privée) [I=5; C=5]	Data minimization	172. We integrated this suggestion.
Software developers are probably the wrong crowd to address this they typically have no control over these things. [I=2; C=4]	Control over carbon footprint	173. See responses no 167 and 168.

High degree of responsibility for the Programming and software eco-responsibility attribute
The adherence to one or more of the elements mentioned. [I=5; C=5]
Use of industry standard methods for benchmarking and reporting on energy consumption level and climate impact, including resources involved in the hardware production. [I=4; C=5]
There are emerging framework, for instance addressing carbon credits, that could be referenced here. [I=2; C=3]
A point on the supply chain is missing. It is responsible for much of the pollution of medical equipment and devices. Probably include the point on ISO 14025. Probably referring to "environmental sustainability" procurement: ISO 20400. [I=5; C=4]
Ideally a label of quality from governmental institution. [I=4; C=4]
The presence of the four practices would constitute a high level of responsibility, and a lower level would be given when one of the practice is absent. [I=5; C=5]
No additional comments here this follows closely on the hardware criterion above. [I=4; C=4]

Procedure - having framework for assessment and making sure that it is given placement in decisions for implementation [I=4; C=4]

Suggestions for additional premises, criteria, and attributes

**Additional premise: No =22; Yes =4**
perhaps you should say something about how this assessment sits within regulatory frameworks, e.g., GDPR and the impending AI Act in the EU, and how it will be updated to accommodate change to regulation in different jurisdictions. It's not clear at this point if the framework can even be taken and adapted or extended by people in different jurisdictions.
It would seem helpful at this stage to define responsibility as a background concept.
Equity in design
I would split premise 2 so you have individual responsibilities and collective responsibilities. Secondly, split premise 4 into digital literacies and access to the internet of things - both of these are related, however should be separate issues for assessment, as one can have access yet not the skills to use technologies and vice versa. Perhaps additional premises could relate to data / digital infrastructure enabling D/AI innovation and dissemination another about governance/regulation D/AI innovations would be subject to whether the D/AI solution is necessary / provides better solution to existing methods and lastly benefits for users / patients / health systems should outweigh benefits to commercial entities or that these should be proportionate to avoid exploitation of data subjects and users.
**Participants who did not complete the Delphi survey**
What about a commitment to building access to services when a given D/AI solution identifies a health concern, otherwise it simply raises anxiety in the user/patient.
Responsible D/AI should validate that there are no ethical violations or concerns of deploying a total on a population or sub population. Example, a prognosticating tool should be validated to purely rely on clinically relevant prognosis and not capture artefacts that merely reflect existing practices. Overall, this is different from existing premises and would require not just external validation but also require some adherence to normative claims
The data sources used to train AI can contain biases and inequities which can be perpetuated. AI developers can, and have a responsibility, to evaluate performance across subgroups and to build the best AI tools they can that perform well and *decrease* disparities.

**Additional inclusion criterion: No =24; Yes =2**
Perhaps this is more an add-on to item 3 but considering how the tool might be used within overall systems of care -- will it be used for resource allocation decisions, for example - and whether there need to be guidance in place before implementation then.
How have stakeholders been involved in the development and decision making of D/AI solutions? Engagement and involvement of stakeholders including members of the public, frontline workers etc. must be included to ensure the benefits and risks are appropriately understood and addressed. Key questions are: 1) Whether stakeholders have been included 2) Which stakeholders have been included and if others have been excluded - why? 3) How have stakeholders been included? 4) How much weight has been given to stakeholders in the decision-making? 5) Impact assessment of stakeholders in the decision-making.

**Additional exclusion criterion: No =22; Yes =4**
There is insufficient scientific evidence to support the claims regarding the ability of the D/AI. Or something to similar affect that aims to address the issues of 'AI snake-oil' such as claims regarding the use of face analysis AI to determine the mental or intentional state of people.

Does the technology provider allow easy access to the algorithm, including for verification purposes if necessary (e.g., transparency, explainability, black box).
It may not be a minimal responsibility criterion, but I think the business entity needs to carry out tests with the target users before starting to sell the products. That is not included here, but one could say it should be included in the minimal requirement.
Where there has been no stakeholder (public, citizen, user, frontline worker etc.) involvement in development and decision-making of the solution. Where there is no plan for future assessment / evaluation of D/AI solution for practice, e.g., machine learning algorithms for clinical practice would learn from future data and as such could change outputs compared to previous input data - these algorithms would need continued assessment of impact from risks and harms.

**Additional attribute for the Population Health value domain: No =24; Yes =2**
I did not want to answer NO, as if I was sure there is no additional attributes that could be required, but also, I do not have any in mind right now. But I am not closed to the idea that there might be more.
Whether it can be utilized equally by relevant subpopulations, particularly marginalized subpopulations

**Additional attribute for the Health System value domain: No =24; Yes =2**
"Human-machine" interface
Developers of D/AI must be representative of the populations the technologies will be applied in to ensure bias/discriminatory development is mitigated against.

**Additional attribute for the Economic value domain: No =25; Yes =1**
Location dependent access to software to deploy could be considered. Some health care settings are in under-served communities and therefore D/AI solutions might take time to reach them. Often decision making is not considering their needs at the early stages. Could relate also to socio-economic class distinctions (not sure if I saw that with the descriptors).

**Additional attribute for the Organizational value domain: No =26; Yes =0**

**Additional attribute for the Environmental value domain: No =25; Yes =1**
Circulation of people, talent (developers, designers, sales, support, etc.) to attract users and deploy systems. There is a cost for sure if people need to physically join each other to realize the project (carbon issue from flying or other transport) but also virtually (use of electricity and bandwidth).

B. 5. Dataset: Expert panel comments gathered at Round 2

The Screening step

**A =** How applicable is this exclusion criterion? 1- Least Applicable; 2; 3; 4; 5- Most Applicable

**Exclusion criterion 1: General Availability stage not reached (A = threshold reached)**		
**Comment**	**Issue**	**Response**
I am not an expert on the product development lifecycle so do not fully understand the nuances of RTM, GA etc. However, I selected 4 because it makes sense that relevant processes should have a chance to be completed before a formal evaluation is made. [A=4]	Agreement	N/A
Je ne suis pas tout à fait d'accord sur le fait que le degré de responsabilité devrait être reporté. Il est même central que ça soit garder pour éclairer justement les étapes précoces de développement et de "dialogue précoces (S'il y'en a) entre l'industrie et les agences d'évaluation et/ou acheteurs. L'outil pourrait alors servir de base pour soulever les éléments de responsabilités qui devraient davantage être respectés. Ça pourrait être une évaluation "formative" de la responsabilité, pas forcément "sommative" à ce stade. [A=4]	Tool as a design brief	174. We reformulated as follows: "No, thus exclude from a formal assessment using this tool (consider using it as a design or procurement brief)"
I think this criterion is quite applicable. I will say, however, that to me General Availability (GA) = commercialization could be limitative. As it could be developed in-house by a public organization, and its various IT services. Therefore, I would maybe broaden the definition of GA to make this criterion more applicable to different contexts. [A=3]	Non-commercial D/AI solutions	175. We added a footnote concerning in-house D/AI solutions: if security, usability, and compliance tests have been conducted, they can be formally assessed using this tool.

**Exclusion criterion 2: Nondisclosure of key D/AI risks (A = threshold not reached)**		
In the information quality assessment, has it been considered in addition to the quality of evidence underpinning the information, that the information is relayed in an understandable manner? Information that is public-facing must be also made available in lay language. [A=5]	Public-facing disclosures in lay terms	176. Thank you for raising the issue. It is covered in the original RIH Tool "Ethical, legal, and social issues" attribute and is therefore addressed in the Assessment step of the adapted tool.
I would (just for clarity purposes, and coherence with the way how questions/answers are worded in the first exclusion criteria) recommend to slightly review the wording of the question and first answer, to something like "Are there public disclosure statements regarding ALL three areas of concern (data reselling, cybersecurity and personal data protection, and AI training datasets)? - No (i.e., there is at least one of the three applicable areas of concern for which there are NO public disclosure statements), thus exclude from a formal evaluation using this tool Moreover, you may want to ensure to write EITHER "public disclosure statements" OR "clear disclosure statements ", whereas currently you have one wording in the explanation and the other in the question/answer section. [A=4]	Wording	177. Thank you for the suggestion. We choose: a) to isolate the area of concern that is only applicable to AI-based solutions; and b) reformulated as follows: "Are there clear public statements regarding these two areas of concern? No, disclosure is missing for at least one area of concern."
I believe combining reselling with Cybersecurity and AI training is not appropriate. [A=3]	Scope of criterion	178. This area of concern is now indicated as only applicable to AI-based solutions.
I am struggling to understand why this is an inclusion/exclusion criterion and not part of an assessment of (ir)responsibility. [A=2]	Rationale of the tool's components	179. Exclusion criteria are basic responsibility requirements. If they are not met, the D/AI solution cannot be considered responsible. The Screening step aims to avoid a situation where the tool would be applied to

		deliver a responsibility score to an 'irresponsible' solution.
The phrasing of the answers to the question "Question to be answered" is clearer in the first item: Yes No, thus exclude from a formal evaluation using this tool Here it is phrased. Exclude from a formal evaluation if the answer is 'no' for any of the three applicable areas of concern Yes, I understand it is more difficult bc three sub-questions that are to be answered. Maybe this could work: Yes No (Exclude from a formal evaluation if the answer is 'no' for any of the three applicable areas of concern) [A=4]	Wording	180. See responses 4 and 5.
Pour "To avoid any ambiguities regarding its core mission, …;". J'ai l'impression que la première phrase contredit la deuxième. Vous dites au début, qu'il faut que l'entreprise soit transparente (enlever l'ambiguïté) en ce qui a trait de sa mission (ou objectif commercial ? pour moi, la mission c'est autre chose). Après, dans la deuxième phrase, vous dites qu'il faut que l'entreprise ne devrait pas revendre les données (je suis absolument d'accord sur ce point). Ma question est : si une entreprise est très claire ET TRENSPARENTE sur le fait qu'elle revend les données, en échange d'un service gratuit. Est-ce qu'elle est responsable ou moins (sans dite irresponsable). Maintenant, dépendamment des contextes, il s'agit d'un positionnement philosophique. Dans un contexte libéral (dans le sens politique: la liberté de l'individu de disposer et vendre ses données -je carricature-), une personne va dire que ça lui convient qu'elle puisse bénéficier du service gratuitement et que ses données soient revendues (en abstraction de la capacité de l'individu de prendre une décision éclairée, etc.). Dans ce cas, là l'entreprise a fait son travail, car elle a bien précisé clairement qu'elle revend les données. C'est son modèle d'affaire. Pour "AI training datasets". Il serait probablement pertinent de mentionner aussi l'importance que l'entreprise puisse donner accès à son algorithme pour comprendre les processus derrières la prise de décision de l'algorithme. Aujourd'hui, de nombreuses entreprises refusent en mettant de l'avant la question de la compétition et du secret commercial. Ça les arrange en cas d'erreur ou préjudice sur le patient. [A=5]	Wording<br>Algorithms open for scrutiny	181. Thank you for these excellent suggestions. We reformulated as follows: "An organization producing a responsible D/AI solution publicly states that it does not sell user-related data."<br><br>182. Making the algorithms publicly accessible may increase robustness when highly skilled individuals are available to test/challenge the AI model. The tool emphasizes more concrete characteristics of responsibility (what has been done, rather than what could be done).
The phrasing of the exclusion criteria currently implies that you need all three to include/exclude. This could lead to more exclusion than necessary, by future readers/users. I personally believe, that while the three areas of concern are very relevant, they definitely target more AI systems that digital non-AI systems. Final remark - question: where does privacy fit? Solely in cybersecurity? This could be debated legally speaking. [A=2]	Wording<br>Privacy	183. Thank you. We added "privacy" in the second are of concern. See responses 4 and 5.

The Assessment step

I = How important is this attribute? 1- *Least important; 2; 3; 4; 5 - Most important;* **C =** Is this attribute clearly defined? 1- *Needs major revision; 2; 3; 4; 5 - No revisions needed;* **A=** Is the scale appropriate? 1- *Needs major revision; 2; 3; 4; 5 - No revisions needed*

**Human Agency attribute (C = threshold reached; A = threshold reached)**		
I would want to see an account of the effort made by developers etc. in ensuring human agency has been actively sought more than for example passively waiting for feedback if and when things go wrong (though accessibility to D/AI decision-making and change should be a multimodal approach). Also, the effort made in outreach with underrepresented groups relevant for the end use population us important. The optimal outcome is active outreach throughout the D/AI lifecycle. [C=4; A=4]	Underrepresented groups	184. Two attributes in the original RIH Tool consider benefits for, and engagement with underrepresented groups: "Inequalities" and "Inclusiveness."
I don't like the label human agency. I think the term doesn't match the sub criteria. I suggest Human engagement or partnership. [C=3; A=5]	Terminology	185. Though the term is not widely used, it brings forward the extent to which individuals have the power (capacity) to use a D/AI solution to achieve better 'Population health' outcomes.

The second paragraph in the attribute definition seemed opaque and unnecessary and made it difficult to follow the overall logic of the section. It is not clear why a statement needs to be made about what is not known, as this is an assessment tool, not a research tool. Suggest deleting it Referring to: "Though D/AI solutions may improve population health by facilitating a range of human decisions and actions, little is known about the way D/AI solutions affect in practice user behaviour, cognition and judgement (e.g., overreliance, avoidance, overconfidence, hypervigilance) and thus their impact on care seeking behaviours and on health and social care provision." Scale Level C could be more clearly worded e.g., "Procedures that EITHER enable users either to understand its outputs or decide and act in accordance with their own goals. There are no formal means for users to have their concerns acted upon" [C=2; A=4]	Clarity<br>Wording	186. We agree with these suggestions and slightly revised both the definition and the scale.
I fell this attribute is very clear, my concern is minor, I don't quite understand why peer pressure is part of the attribute: "decide their own preferred course of action without undue pressure from the D/AI solution itself and from peers" [C=4; A=5]	Peer pressure	187. Peer pressure may limit human agency in professionalized settings like clinics and hospitals, in schools or in workplaces.
Le seul défi que je vois ici est celui de la capacité des gens à prendre la décision (selon leurs objectifs: ça rappelle un peu la théorie de la justice et les "capabilités"). Les personnes et groupes ne sont pas égaux devant l'information "disponible" pour prendre une décision éclairée. Ça renvoi à la théorie de la rationalité limitée de Simon (entre autres): c'est l'idée selon laquelle la capacité de décision d'un individu est altérée par un ensemble de contraintes comme le manque d'information, des biais cognitifs ou encore le manque de temps. Dans cette optique, la personne a tendance à choisir des solutions satisfaisantes plutôt qu'optimales. Et là, ça renvoi encore à la notion des capabilités ("what a person is able to do or be"): ceux qui n'ont rien se contentent de rien, ceux qui ont peu se contentent de peu. Tout un défi. [C=4; A=4]	Theoretical precisions	188. We agree that capabilities vary across individuals and groups.
I think could have been added the option: D. include formal means to have users' concerns acted upon but does not have procedures that enable them either to understand its outputs or decide and act in accordance with their own goals. I think it is possible to be offered a way of formally complaint, or share concerns, or have the company supposedly integrate them. But the other procedures might be weak or worse absent. [C=4; A=3]	Scale	189. You are right, this combination is possible. See response 13.

**Care-centric interoperability attribute (I= threshold reached; C = threshold not reached; A = threshold reached)**		
sometimes "data exportation" is used, sometimes "data sharing". I believe that "data exchange" without proprietary protocols is better? [I=4; C=3; A=4]	Wording	190. We retrieved "data exportation."
I prefer the term 'is embedded' rather than aligns with In : e.g., 'following the patient' across care settings when relevant I would : patient' across care settings AND CLINICAL OR ADMINSTRATIVE WORK FLOW when relevant [I=5; C=3; A=5]	Wording	191. We reformulated the sentence for clarity.
Suggest switching the order of the first two paragraphs so the general rationale comes before the description of what the attribute refers to. -Not clear why 'aligns' is in bold text -This wording is awkward: "that are well-thought through administrative processes and clinical pathways" -Why is there nothing about digital infrastructures in part C of the scale [I=no response; C=2; A=3]	Structure<br>Wording<br>Scale misses an element	192. We chose not to reorder these paragraphs because the logic across the tool is to first define the attribute and then provide its rationale. We, however, revised the scale to increase the correspondence between the definition and the levels of the scale.
"Testing the D/AI solution in the context of use before its full deployment and regularly assessing how it interfaces with users' evolving digital" +> For me it seems that "before its full development" is assessing a phase that might be at the before G/A stage - and thus the D/AI solution would be excluded from formal assessment. Also, the "testing before its full deployment" is not mentioned in any of the scale levels A-D, so maybe it should not be present here (although I do believe it is an essential step -but it does not seem to help with the evaluation in the frame of this tool) When reading "B- Is periodically adjusted to fit users' digital infrastructures and aligns with their data management practices, but provides limited data sharing functionalities" I have a feeling that it blurs the line between the two first points of attribute definition. Because the first point says: "	Scale misses elements (testing, adjustments)<br>Inadequate data infrastructures or practices in health settings	193. We reformulated the attribute and the scale, and retrieved "testing the D/AI solution…"

aligns with user capabilities, needs, work processes and task allocation to minimize cognitive and administrative burden;" Here, ALIGN seems to refer to individuals' capabilities, needs, etc., ... While in the rating scale, ALIGN seem to refer to a system. It might well be that the system of data management practice in place is not optimal. Also, I believe part one of the first point " Designing a solution that is operable on widely available systems and devices", and last part of point 2 "and using non-proprietary software or solutions that facilitate data exportation" are quite similar. When reading the attribute, I can make a distinction between point one and two , but when I read B on the scale, suddenly I'm not so sure about the distinction. - So, I believe either something should be clarified in the attribute description - or in the way the scale point B is phrased. Maybe it is because "data exportation" is used in attribute description, but "data sharing" is used in the scale? Also, I wonder why nothing is mentioned about adjustment in C and D. I know you don't want to make a scale that looks for "defects" but rather looks for "what's there". But here, it seems to me that the main difference between B and C is in fact the absence of regular adjustment and clearly stating it would make the scale more transparent for users. Doing so would perhaps also allow you to have D on the scale stated in a similar way as you state it for other attributes "No particular human oversight procedures" e.g.: C-Either requires substantial adaptations to users' data management practices or provides limited data sharing functionalities and is not periodically adjusted to fit user's digital infrastructures D-No particular care-centric interoperability functionalities [I=5; C=4; A=3]	Wording	
Ici, vous soulevez carrément (du moins indirectement) la question de l'implantation de la technologie. Aucune technologie ne vient prête à l'utilisation ("plug and play"). Je suis totalement d'accord sur le fait que la technologie puisse s'adapter, etc. Je ne sais pas si c'est pertinent, mais je pense qu'il faudrait aussi que le fournisseur puisse s'engager à ce que la technologie évolue et s'adapte au besoin et réalités technologiques des utilisateurs finaux et qui peuvent changer/évoluer dans le temps. L'un des défis dont sont confrontées les organisations, c'est que certains fournisseurs les "enchainent" dans une relation de dépendance, ce qui ne leur permet plus d'intégrer d'autres technologies de fournisseurs différents, même à grande valeur ajoutée et plus "efficiente". Ça explique la situation d'infrastructures "désuètes" dans certaines organisations: elles sont incapables de les faire évoluer et intégrer de nouvelle tech. [I=5; C=4; A=4]	Developers' commitments and lock-ins<br>Inadequate infrastructures or practices in health settings	194. We agree that developers may increase interoperability problems.<br>195. See response 20.
The only question I have is "who would answer - Requires substantial adaptations to users' data management practices and provides limited data sharing functionalities ". If I am part of an organization, it feels like this would require a tremendous amount of insight, honesty, or transparency to state it during a self-assessment. Therefore, I would maybe rephrase it slightly to make it easier for this category of assessors to choose this option. If it is an external assessment, it completely makes sense. [I=5; C=4; A=4]	Applicability	196. We revised the level D of the scale.

**Software frugality attribute (I= threshold reached; C = threshold reached; A = threshold reached)**		
Attributes appears simply as software development good practices here; I do not clearly see the link with frugality.... maybe this? https://digitalprinciples.org/ [I=2; C=2; A=2]	Frugality in software	197. Thank you for the website reference, which is a great resource for D/AI solution developers. We relied on the frugal innovation literature to identify these three key characteristics.
One thing you may add in the definition of the attribute (i am unsure whether it would better fit under "affordability" or "optimized performance", probably the latter) is that the D/AI solution should not generate some kind of 'externalised' costs (for lack of a better word). Meaning that, for example, having a clinical D/AI solution which is affordable in itself (e.g., a clinical software that does not cost much), but requires a lot of (costly) hours of training for clinical personnel that wants to use it. I think you already point at this issue when you mention the example of "data plans being unaffordable", but you could be more explicit. Again, the key issue would be not having a D/AI solution which is cheap by 'externalising' costs thereto related (e.g., to the training needed for personnel who wish to use it, or e.g., to expensive data plans). [I=5; C=4; A=4]	Shifting financial and human costs to users	198. This is an excellent point. The notion that a frugal innovation must be "optimized for its context of use" as well as designed for increased "ease of use" can capture whether 'cost' shifting is an issue.

Suggest removing bold text from "characteristics" or bolding all of "frugal innovation characteristics" I like the simplicity of this scale and I wonder whether others that have three or four key elements could be similarly simplified. [I=4; C=4; A=5]	Appreciation of the scale	199. Thank you. Many of the original RIH attributes have similar scales.
Petite question : est-ce que la possibilité d'adapter la technologie à plusieurs utilisation fait partie de la frugalité. Une sorte d'optimisation et de maximisation du potentiel de la technologie. Au lieu d'acheter une technologie pour un usage. ? Ce point revient assez souvent dans l'implantation des technologies numériques. Par exemple, un dispositif de télépathologie a été utilisé par les cliniciens pour faire de l'autopsie, téléconsultation, télégynécologie, etc. Ces différents usages n'ont pas été planifiés par le MSSS ni les organisations au début du projet. Ils n'étaient même pas inclus dans les indicateurs d'utilisation de la technologie. [I=5; C=4; A=4]	Theoretical precisions	200. Fugal innovation designers certainly look for such opportunities because they increase the number of potential users while maintaining a focus of core functionalities.
As you state in the introduction, it is very possible that one criteria in a specific context makes it completely acceptable "For example, in certain situations, it may be legitimate to have a lower score on the Health relevance attribute to reach a higher score on the Eco-responsibility attribute (or vice versa)" I am openly wondering if the option "One characteristic of software frugality" is therefore that revealing of the total lack of frugality - if let's say in a specific context Affordability was the key, most difficult thing to accomplish and could in the said context demonstrates a major effort to be "frugal". Sorry if it does not make sense. [I=no response; C=5; A=5]	Theoretical precisions	201. Thank you for the question. A 'C' on the scale does not imply a lack of frugality (a D' does). "Affordability" of a given D/AI solution may indeed be appraised in view of the currently available solutions (or cost of not intervening).

**Data governance attribute (A = threshold reached)**		
This criterion might not be applicable uniformly across all solutions. What about a solution that trained on publicly available data? No data governance involved here, that would be NA. [A=2]	Applicability to publicly available datasets	202. This is an intriguing point! It suggests that datasets 'downloaded' from a public source would not require data governance. We rather believe that good data governance practices are an important responsibility feature, no matter the origin of the datasets.
À mon avis, il faudrait préciser qu'est-ce que vous entendez par "a third party". Je comprends à quoi vous faites références, mais il serait pertinent de rajouter le qualificatif "indépendant, externe, certifié, reconnu" . Il serait probablement pertinent que la gouvernance des données, puisse être vérifier par des "Crash testing" (mais il y'a d'autres types : Smoke testing / Comprehensive Testing"), qui permettent de vérifier, évaluer la solidité du logiciel, aussi bien le code que les éléments en lien avec la question de la gouvernance des données. "Crash testing is the innermost technique operates on each code check-in of the GUI software and it is executed frequently with an automated GUI testing intervention and performs quickly also. It reports the software crashes back to the developer who checked in the code. Smoke testing is the second technique operates on each day's GUI build and performs functional reference testing of the newly integrated version of the GUI, using the previously tested version as a baseline. Comprehensive Testing is the outermost third technique conducts detailed comprehensive GUI integration testing of a major GUI release and it is executed after a major version of GUI is available. Problems are reported to all the developers who are part of the development of the particular version" Voici la référence au besoin : http://www.airccse.org/journal/ijsea/papers/3112ijsea10.pdf [A=5]	Wording<br>Robustness testing procedures	203. We added 'independent' third-party.<br>204. There are indeed different strategies to ascertain whether data is robustly managed or not. Our scale captures organizational-level strategies that can include strategies at a more technical level.
I think "high-level team accountable to the board of directors" is maybe too narrow. You could add "high-level team accountable to a board of direction or any internal referring structure" [A=4]	Wording	205. We agree and have revised accordingly.

Programming and software eco-responsibility attribute (I= threshold reached; A = threshold not reached)		
This is mostly impossible to control by D/AI developers IMHO... [I=2; A=2]	Feasibility	206. Though they may not have the final say in corporate decisions regarding infrastructures, they choose the "programming, modelling or computation techniques."
I understand that it is difficult making an "exhaustive list" in the attribute, and thus you write "may include" and then list 'only' three (acknowledging that there may be more). However, a risk in doing this, is that it might be difficult to evaluate a tool which may have some "practices of programming and software eco-responsibility" which - however - differ from the three you listed. Thus, why not having (in the scale section) something like The D/AI solution relies on: A. "Three practices of programming and software eco-responsibility or more. If such practices differ from the ones explicitly listed in the attribute, there must be a clear explanation as to why they count as "practices of programming and software eco-responsibility" B. Two practices of programming and software eco-responsibility. If such practices differ from the ones explicitly listed in the attribute, there must be a clear explanation as to why they count as "practices of programming and software eco-responsibility" C. One practice of programming and software eco-responsibility. If such practice differs from the ones explicitly listed in the attribute, there must be a clear explanation as to why it counts as "practice of programming and software eco-responsibility" D. None of the programming and software eco-responsibility practices [I=4; A=3]	Wording<br>Other potential eco-responsible practices	207. We have revised the definition and the scale accordingly.
I feel it is strange that A is stating: "three practices or more" while only 3 types of practices are described. I believe this makes it a bit confusing because it leaves the door open to interpretation. Like, if I evaluate a D/AI solution that has either programming, techniques that substantially reduce the quantity of energy and time required to develop a D/AI solution (e.g., tinyML) but not modeling or computational techniques - I would rate that as "one practice"; and if this solution also uses highly energy efficient CPUs, that would also be "one practice" - the solution would be given a B for 2 practices. But if the D/AI solution has e. g. programming AND modeling AND computational techniques that ... - but nothing else, should I rate it as "one practice" (C) or would I count that as 3 practices and rate an A? [I=5; A=4]	Definition and counting of 'practices'	208. See response 34.
I absolutely love the attribute, love the propositions. But re-reading them a few times, I think they could be complemented by quite a few other practices. I recently worked on calculation methods of CO2 emissions of machine learning algorithms. And we tried to apply it to one of our small-scale AI projects. It faired poorly. That made me think that the options that you list might (in my view) make more sense for large-scale projects, where (in the best case scenario) all the information about what third-party services offer/use/do (e.g., AWS). But for smaller projects or tools that would use your tool, you might always get the worst answer because it is hard to get any of the options you highlighted (which is also a possibility that reflects poorly on the lack of consideration of eco-responsibility in AI for instance). [I=5; A=4]	Agreement<br>Other potential eco-responsible practices	209. Thank you for these insights. We agree that the practices we listed may not exhaust all possibilities but adopting a large-scale perspective on the organization's eco-responsibility practices is better aligned with the intent and scope of RIH.

Premises

I = How important is this premise? 1- Least important; 2; 3; 4; 5 - Most important; **C =** Is this premise clearly defined? 1- Needs major revision; 2; 3; 4; 5 - No revisions needed

The context of use largely shapes responsibility (I= threshold reached)		
ABSOLUMENT d'accord. Toutefois, si on se met un peu dans l'idée de la théorie de la structuration (les structures, ensemble de règles et de ressources, organisent les activités tout autant que les activités les organisent et leur donnent du sens et une finalité), le concept de "responsabilité" va en retour façonner le contexte. C'est une relation dynamique dans les deux sens. La prise en compte de la responsabilité pourrait à la fois être le produit et le producteur de son contexte. [I=4]	Agreement<br>Theoretical precisions	210. Thank you. This precision may be useful in future work on the implementation and uptake of the tool.

I would just add one missing element: moral differences and tolerance limits can vary greatly from one context/country to another. [I=5]	Moral variations	211. We added "moral" in the definition.
**Responsible D/AI solutions aim for collective benefits (I= threshold reached C= threshold reached)**		
Of course, support collective benefit BUT iNDIVIDUAL benefit deserves equal credit! Both individual and collective benefits are equally important and have equal equity implications! e.g. individuals in rural populations e.g. as long as everyone has access both rich and poor INDIVIDUALS receiving new virtual care e.g. primary care, covid care with o2 sensor, remote cardiovascular monitoring, HIV monitoring, dermatology remote diagnosis , computer controlled remote surgery, videos to show mothers how to replace gastric tubes for failure to thrive etc. .Should get full marks if they meet the other Responsibility Criteria! Our CIHR grants on stakeholders in systematic reviews and guidelines [with the GRADE group] and out Cochrane and Campbell Equity Group active in advocating for equal individual and community equity responsibility [although we use different words] Happy to discuss [I=3; C=1]	Individual benefits	212. Because many D/AI solutions are geared at delivering individual benefits and because the tool attributes do not create an imbalance towards D/AI solutions geared at collective benefits, we chose not to put on equal terms individual benefits and collective benefits in this premise.
My suggestion : As a result, they MIGHT overlook key opportunities to address through other types of D/AI solutions either the causes of ill-health in large groups of people (e.g., air pollution and cardiovascular diseases) or ways to reduce or eliminate health risks for the collectivity as a whole (e.g., legislation on soft drinks or ultra-processed foods). The examples given are not appropriate I think for D AI solutions. I suggest (e.g.: bad life habits) This paragraph is not clear. I don't see the link between one sentence to the other. It doesn't flow well hose who suffer from ill-health are exposed to health risks that cumulate over their life course, lead to more complex comorbidities and exacerbate the mental and physical "wear and tear of daily life." From a population health perspective, D/AI solutions should be shaped by a thorough understanding of Why are some people healthy and others not? The last sentence is important: Those who apply the tool should thus recognize that although a D/AI solution that provides individual health benefits is valuable, a responsible D/AI solution should aim for broader collective benefits. [I=5; C=3]	Examples not appropriate<br>Clarity	213. We revised the examples. Yet, we consider that "bad life habits" are not a good example because they may result from a lack of human agency and/or a lack of practicable options for certain social groups to engage in and maintain a 'healthy life' trajectory over their life course.<br>214. We improved both legibility and conciseness
Could be shortened [I=4; C=4]	Conciseness	215. See response 41.
I feel very divided about this premise. It seems to assume that D/AI solutions emerge from engineers that are (apart from "making AI") "discipline neutral". I am thinking for example about Parkinson's Disease (PD), where there is now a growing consensus that air pollutants might be part of etiology of the disease. Our team of speech language pathologists (SLPs), persons with PD and engineers are looking at a D/AI solution to make SLP services more accessible. We can recognize that a larger solution (not necessarily D/AI) is requested to address the systemic problematic leading to the development of the disease, but these solutions are much more long term and our own expertise are not adequate to address this larger problematic. I feel that there is a limit to the extent of population health perspective a specific D/AI tool is capable of including without exhausting the team. But I recognize and strongly believe that the population health perspective is crucial to embed in all our "disciplinary" health perspectives (and that is not specific to D/AI), so I wonder how this premise could be more appreciative of this fact without minimizing the need for D/AI solutions to address or be mindful of the larger population health perspective. Maybe it is something about the phrasing of the last section " Those who apply the tool should thus recognize that although a D/AI solution that provides individual health benefits is valuable, a responsible D/AI solution should aim for broader collective benefits." Who are "those who apply the tool"? It is the users and not the developers? I feel both the developers, and those who deploy and use the tools should recognize that. I feel we can have it as a premise that those who develop D/AI solutions should recognize this fact - but I don't feel we can push it on users - but rather that the developers should be transparent with this. What is "recognize"? - maybe give an example of how this could be recognized - is it through a value statement? Is it something else? I feel this premise very important, and the mere fact that is divides me makes me aware that there is work here to be done. So, I definitely think this premise should be maintained. [I=5; C=4]	Difficulty in adopting a population health approach<br>Wording	216. Thank you for this real-world example. A population health perspective can be supported by having public health experts on the team and by documenting the root causes of the problem one seeks to tackle.<br>217. We clarified what we meant by "those who apply the tool."

It speaks to my heart! It is also possible that a user might not completely agree with it. And therefore, the question could be: does a user need to agree with this premise to use it. Second question: is then your score of responsibility fully dependent on it (the answer is yes - but what if a user does not fully endorse your premise?). [I=5; C=5]	Agreement<br>Rationale of the premises	218. See response 48.
**D/AI solutions should tangibly improve current processes and means (I= threshold reached C= threshold reached)**		
One main issue is that there is a growing global shortfall in healthcare workforce numbers, so D/AI solutions may be the only course of action in some instances e.g., in low-risk applications such as back-office functions, though in high risk clinical applications ensuring the technologies work without negatively affecting patient safety and clinical burden are paramount. However, there does need to be due diligence in understanding the context in which they are to be implemented in and support provided for successful adoption. [I=5; C=5]	Agreement<br>Healthcare context	219. Thank you for your insight.
Suggest should demonstrate meaningful improvement in 'Health or Social Outcome ' or accept increased efficiency only if health and social outcomes not worsened [I=5; C=1]	Wording	220. See response 48 below.
Please specify: Those who apply the tool should thus examine whether the relevance of the D/AI solution is compelling and supported by research. I agree with this sentence, but it is a bit vague. How do you assess the relevance? [I=4; C=5]	Rationale of the premises	221. The premises are not used to assess responsibility but to guide evaluators' reflections on the broader issues raised by the D/AI solution under assessment. Only the rating scales of the attributes assess the degree of responsibility and are used to calculate the score.
**D/AI solutions modulate determinants of health (I= threshold reached C= threshold not reached)**		
Not sure if this aspect should be covered as determinants of health is a complex topic. [I=3; C=3]	Rationale of the premises	222. We agree about their complexity, but evaluators should be aware that determinants of health affect the responsibility of D/AI solutions.
Those who apply the tools should actively look for ways to support the underserved. Those who propel the technologies must plan for and identify potential inequities and offer ways to minimise and mitigate them. This may not be a straightforward process and could be done over time with bottom up approaches. [I=5; C=5]	Inequalities	223. Thank you. The "Inclusiveness" attribute of the original RIH Tool emphasizes user engagement and is part of the adapted tool.
Hmm - this sounds too much like an apple [pike/ motherhood statement but needs disaggregating into measurable desired and undesired behaviours e.g., computer /digital literacy should be checked and a program to ensure everyone receiving the AI is if necessary trained before the AI gets a high Responsibility Score [I=5; C=1]	Rationale of the premises	224. See response 48 above.
I struggled to understand whether this was meant to be about digital solutions that address the social determinants of health or about the social determinants of access to digital technologies. I think it is the latter, but the title made me think it was the former so I was a bit confused. [I=no response; C=2]	Clarity	225. We eliminated the ambiguity.
À mon avis, le numérique n'est pas juste un modulateur des autres déterminant. C'est un déterminant qui devrait être abordé en tant que tel. À la limite, elle peut être les deux, selon les contextes : modulateur d'autres déterminants ou un déterminant à part entière Lien : https://www.nature.com/articles/s41746-022-00663-0 https://www.nature.com/articles/s41746-021-00413-8?mc_cid=714c29799f&mc_eid=98117185bf&mc_cid=113a62c674&mc_eid=bce6925769 https://jamanetwork.com/journals/jama/fullarticle/2785583 [I=4; C=5]	Clarity	226. See response 52.

**End of survey comments**
Thank you for this huge work! I look forward to using this tool.

Thank you for the involvement in the development of the tool! I hope my comments are of use.
1. Congrats on this excellent introductory video. Really like your Value framework 3. My major interest in in the inequality, equity sections as you will see from my comments. I would like to challenge several issues. 4. Look forward to seeing how this applies to a diversified sample of D/AI solutions
Thank you for this important work.
Bravo! Thanks for this much-needed work! Un grand merci, c'est un travail incroyable. I think I would advocate for a separate tool just for AI, Data and Robotics systems. It feels that a lot of questions and concerns (throughout your tool) leads to these technologies and could benefit from a specific adaptation / version of your future tool. (I should state that I understand why it makes sense to include them in a larger context - but still).
Juste BRAVO pour un aussi complet et conséquent travail.

C. Phase 3- Supplementary material

C. 1. Selection and documentation process of the D/AI solutions

The objective of Phase 3 was to assess the reliability of the Tool and bring measurement revisions if needed. Following Gwet's recommendations, an error margin of ±0.20 was used to determine our sample size, that is, 25 D/AI solutions [36]. To create a balanced and diversified sample of real-world solutions, we followed three steps.

Step 1: Preliminary list of D/AI solutions

We reviewed 5 relevant sources of information describing several types of D/AI solutions to define and test eligibility criteria:

- 34 solutions: AI-based devices approved in the United States of America by the Food and Drug Agency (FDA) from 2019 to 2021 [43, 44]
- 28 solutions: The World Health Organisation Classification of Digital Health Interventions [41]
- 30 solutions: Areas For Future Action in the Responsible AI Ecosystem [42]
- 47 solutions: The Canadian Agency for Drugs and Technologies in Health (CADTH) Health Technology Trends to Watch in 2021 [47]
- 291 solutions: AI for Sustainable Development Goals (AI4SDGs) Think Tank [48]

We searched the Internet and included 45 D/AI solutions using the following 4 eligibility criteria. The solution: 1) meets the definition of a digital solution operating with or without AI; 2) addresses at least one determinant of health; 3) is already available for use (in the Americas, Africa, Asia, Europe, or Oceania); and 4) its developer makes freely available the information required to document the criteria and attributes of the Tool in English, French, or Portuguese on its website.

Step 2: Final sample of D/AI solutions

Based on the preliminary list of D/AI solutions, our team members, co-researchers, and collaborators validated the selection of 25 solutions that constituted a balanced and diversified sample by including solutions operating with or without AI, pursuing different purposes, developed by diverse organizations, and used in different regions. Table 4 shows how the final sample met our diversification criteria.

Criteria	Source of diversity	Number of solutions
**Type of D/AI solution**	Operating with AI	13
	Operating without AI	12
**Purpose**	Prevention	5
	Self-care and self-management	5
	Diagnosis	5
	Treatment	5
	Health and social services management	5
**Context of use**	Clinical settings	8
	Non-clinical settings	13
	Clinical and non-clinical settings	4
**Regions**	Africa	3
	Americas	7
	Asia	2
	Europe	1

	Oceania	1
	More than one continent	14
**Type of developer**	For-profit organizations (include private hospitals and clinics)	14
	Not-for-profit organizations (include universities and NGOs)	8
	Public agency or informal (user-led) associations	3

Table 4. Overview of the sample of D/AI solutions used to assess interrater agreement

Step 3. Documenting the 25 D/AI solutions for assessing interrater agreement

For the two raters (RRO and LR) to apply the Tool as intended, we searched the website of each solution in our final sample to collect information addressing the Tool's criteria and attributes (terms of refence, privacy or sustainability policy, user guides, governance structure, annual reports). We tabulated relevant excerpts for all 25 D/AI solutions in an Excel 'scorecard' that both raters completed independently. Because start-ups tended to share less detailed documentation than large firms, PL adapted the content found on other developers' websites for the scorecard to contain all the information needed to score each criterion and attribute for all 25 solutions.

We provide below anonymized examples of information found or adapted for each screening criterion and assessment attribute.

Nondisclosure of D/AI risks (applicable to both digital and AI-based solutions)

Developers do not disclose the non-resale of data. Personal data relating to users are kept for a period of 6 months, then deleted. The statistics resulting from the use of this information can be kept for up to 25 months, then deleted, or anonymized. The user has the right to access, rectify, delete or export his or her data, or to oppose the processing of his or her data by [name of company] (or to request its limitation).

Nondisclosure of D/AI risks (applicable only to AI-based solutions)

[Name of company] is the most extensively validated AI technology in the world. It has been validated in a pivotal, prospective, multicenter clinical trial against the rigorous clinical reference standard using the Early Treatment Diabetic Retinopathy Study (ETDRS) grading scale by experts at the University of Wisconsin Reading Center. It has been tested in a clinical validation study on over 100,000 patient visits, one of the largest data sets used to test any available diabetic retinopathy screening technology, in demanding, real-world clinical environments using images captured in everyday practice. It has also been independently validated by UK NHS in a study with over 30,000 patients.

GA stage not reached

[Name of product] is available for purchase on the developers' website. It has obtained US FDA clearance.

Human agency

We work with a network of vetted linguists to translate messages and evaluate system accuracy to ensure our translations are of the highest quality. Overall, 99% of the messages sent through [name of product] offer human translation support. In-product features like on-demand "help me understand" human-reviewed translation and videos with translated captions are just a few examples of innovative tools we've built into [name of product] to support understandable, accessible communication. Teachers and families find in-app tips and guidance to support positive relationship-building. Data for personalization: we measure feedback

and actions of families and educators to refine what in-app coaching content is helpful and useful to build capacity, and for whom, allowing us to eventually personalize the experience.

Care-centric interoperability

The [name of product] is used widely as a job aid by skilled birth attendants working in the periphery of the health system in low- and middle-income countries. The app works offline once downloaded, so healthcare workers in even the most remote settings can always refer to it. The app consists of 12 content modules addressing key interventions of childbirth emergencies and preventative procedures (infection prevention, prolonged labour, removal of placenta, etc.), all aligned to international clinical guidelines. The app can be used during various types of in-service trainings as a teaching aid for the training instructor and as a study aid for the training participants to support their on-going professional development.

Data governance

Machine learning model governance and transparency are fundamental to [name of product] predictive power. Our company strongly believes that data driven solutions are only as good as the data they rely on, that transparency and accountability are the only path to trustworthy AI, and that vast amounts of data means endless possibilities for data disorganization. Because our company's predictive power relies on optimizing the data lifecycle (e.g., facilitating rapid and consistent labeling, processing, and querying for operational machine learning), our Head of ML Science is accountable to the Board of directors for ensuring that [name of product] data governance practices remain aligned with a high-quality data-driven mission. This includes regularly reviewing how employees as well as any (authorized or non-authorized) third parties access our databases, report on, and correct any uses that deviate from our policy.

Programming and software eco-responsibility

Our Sustainability team develops and implements our environmental strategy. The team is responsible for setting targets to reduce energy use by our programming units and reporting annually on our progress. Our Chief Financial Officer is responsible for facilitating the ongoing assessment and audit of our ISO 14001:2015 Environmental Management System (EMS). In 2019, we completed the required external audits to certify our EMS to the updated ISO 14001:2015 standard.

C. 2. Characteristics of the final sample of 25 D/AI solutions

ID	Description	Internet *	Purpose	Context of use	Main user	Region*	Developer
S.01	A desktop and mobile phone application operating with AI	Yes	To translate text and audio into American Sign Language (ASL) and Brazilian Sign Language (Libras)	Non-clinical setting	Portuguese or English speaker living with a hearing impairment	Americas	For-profit organization
S.02	A mobile phone application operating with AI. It requires a device paired to the mobile phone to capture the cardiac signals on the fingertips (proprietary hardware platform)	No	To record, store, and transfer single-channel electrocardiogram (ECG) rhythms	Non-clinical setting	Person experiencing cardiac arrhythmia that requires monitoring	Americas	For-profit organization
S.03	A desktop cloud-based software (proprietary software platform) operating with AI. It requires a device that captures the retinal images	Yes	To enable automated diabetic retinopathy screening	Clinical setting	Non-specialist clinician dedicated to the DR screening	Americas; Europe	For-profit organization
S.04	A software operating with AI embedded in smart glasses/electronic eyewear (proprietary hardware platform)	No	To enhance visual acuity up to 20/20	Non-clinical setting	Person living with central vision loss	Americas	For-profit organization
S.05	A mobile phone application operating with AI (wayfinding application). It requires an GPS access and a geolocation infrastructure (on the site)	Yes	To offer step-by-step guidance for people living with a disability to get around	Non-clinical setting	Person living with visual, hearing, cognitive or mobility impairment	Americas; Europe	For-profit organization
S.06	A chatbot software (proprietary software platform) for website operating with AI (Artificial Intelligence chatbot symptom checker)	Yes	To automate the interaction between users and providers in healthcare facilities	Non-clinical and clinical settings	User or manager of a health care facility	Americas; Asia	For-profit organization
S.07	A chatbot embedded in a mobile phone application operating with AI	Yes	To check users' symptoms and concerns to offer a relevant course of action	Non-clinical setting	User of a health care facility	Americas	For-profit organization
S.08	A mobile phone application operating with AI	No	To generate a score based on physical and social infrastructure parameters to assist people in making a safer decision in terms of mobility	Non-clinical setting	Person concerned about mobility safety issues	Asia	Not-for-profit organization
S.09	A solar-powered acoustic streaming cloud-based software operating with AI (proprietary software platform) embedded in a proprietary hardware. It requires GPS access	Yes	To enable the bio-acoustic monitoring of rainforests	Non-clinical setting	Authorities or researchers	Africa; Americas; Europe	Not-for-profit organization
**S.10**	A desktop and mobile phone application operating with AI	Yes	To enable communication via text message between teachers and parents or guardians in more than 100 languages	Non-clinical setting	School administrator, teacher and parents or guardians	Americas; Asia	Not-for-profit organization
**S.11**	A data-centric platform (proprietary software platform) operating with AI	Yes	To analyze data from a health care facility to offer personalized and adapted interventions	Clinical setting	Clinician and manager of a health care facility	Africa; Europe	Not-for-profit organization
**S.12**	A mobile phone application operating with AI (machine learning platform)	Yes	To provide skilled birth attendants in low- and middle-income countries access to evidence-based clinical guidelines to handle the most common childbirth emergencies	Non-clinical and clinical settings	Skilled birth attendant	Africa	Not-for-profit organization
**S.13**	A mobile phone application operating with AI. It requires GPS access	Yes	To find and alert the contacts of people infected with the coronavirus	Non-clinical setting	Authorities and general population	Asia	Public agency

ID	Description	Internet *	Purpose	Context of use	Main user	Region*	Developer
**S.14**	A mobile phone (only android) app operating without AI. It requires access to proprietary desktop software	No	To enable healthcare data collection in an offline mode	Clinical setting	Manager of a health care facility	Africa; Asia	Not-for-profit organization
**S.15**	A desktop and mobile phone application operating without AI	Yes	To enable the self-management of people living with chronic lung disease	Non-clinical and clinical settings	Person living with chronic lung disease and clinician	Europe; Oceania	For-profit organization
**S.16**	A (proprietary) software operating without AI embedded in a dual-energy detector for fixed or portable X-ray machines	No	To enhance the image capture quality of conventional x-ray machines	Clinical setting	Clinician or manager of a health care facility	Americas	For-profit organization
**S.17**	A mobile phone application operating without AI. The smartphone camera must be connected to a colposcope	No	To enhance the image capture quality and documentation of conventional colposcopes	Clinical setting	Clinician	Africa; Americas; Asia; Europe	For-profit organization
**S.18**	A (proprietary) software operating without AI embedded in a (proprietary) bioprinter	No	To print multicellular tissues with complex geometries and incorporate vasculature into thick tissues	Clinical setting	Clinician or researcher	Americas	For-profit organization
**S.19**	A mobile phone application operating without AI. The smart phone must be connected to an ultrasound probe (proprietary hardware)	No	To enable image capture with a portable ultrasound device (point-of-care)	Clinical setting	Clinician	Americas; Asia	For-profit organization
**S.20**	A desktop and mobile phone application operating without AI	Yes	To enable the development of customized apps for healthcare facilities in low-infrastructure environments	Clinical setting	Manager of a health care facility	Africa	Informal association
**S.21**	A desktop and mobile phone application operating without AI	Yes	To enable objective indicators of mental state to be catalogued by a person responsible for someone with intellectual disabilities who has communication impairments	Non-clinical and clinical settings	Person responsible for someone with intellectual disabilities and communication difficulties	Oceania	Not-for-profit organization
**S.22**	A desktop and mobile phone application operating without AI	No	To enable indigenous and other local communities to map and share place-based stories	Non-clinical setting	Indigenous and local communities or researchers	Africa	Informal association
**S.23**	A desktop and mobile phone application operating without AI. It requires GPS access	No	To enable to document and record GPS points of significant places in local communities	Non-clinical setting	Indigenous and local communities or researchers	Africa; Asia	Not-for-profit organization
**S.24**	A (proprietary) software embedded in a virtual reality helmet (proprietary hardware)	No	To offer a virtual reality-based treatment for people living with chronic lower back pain	Non-clinical setting	Person living with chronic back pain	Americas	For-profit organization
**S.25**	A desktop and mobile phone cloud-based (proprietary) software operating without AI	Yes	To enable to continuously monitor the microclimate and manage every process on a vertical farm	Non-clinical setting	Manager of a vertical farm	Europe	For-profit organization

Table 5. Characteristics of the 25 D/AI solutions

Notes: * Whether Internet connection is required to operate the D/AI solution. ** The continent where the solution is currently in use according to the developer's website.

C. 3. Dataset: Raters' scores on each component of the Tool

	Inclusion criterion		Exclusion criterion 2a		Exclusion criterion 2b		Exclusion criterion 3		Human agency		Care-centric interoperability		Software frugality		Data governance		Programming & software eco-responsibility	
**Solution**	**Rater 1**	**Ratter 2**	**Rater 1**	**Ratter 2**	**Rater 1**	**Ratter 2**	**Rater 1**	**Ratter 2**	**Rater 1**	**Ratter 2**	**Rater 1**	**Ratter 2**	**Rater 1**	**Ratter 2**	**Rater 1**	**Ratter 2**	**Rater 1**	**Ratter 2**
**S.01**	YES	YES	YES	YES	NO	NO	YES	YES	4	4	2	2	4	4	2	2	4	4
**S.02**	YES	YES	YES	YES	NO	NO	YES	YES	1	1	5	5	1	2	4	4	4	4
**S.03**	YES	YES	NO	NO	NO	NO	YES	YES	1	1	5	5	2	2	4	4	2	2
**S.04**	YES	YES	NO	NO	NO	NO	YES	YES	4	4	5	5	2	2	1	1	2	2
**S.05**	YES	YES	NO	NO	NO	NO	YES	YES	5	5	2	2	4	4	4	4	2	2
**S.06**	YES	YES	YES	YES	NO	NO	YES	YES	4	4	5	5	2	2	1	1	1	1
**S.07**	YES	YES	YES	YES	NO	NO	YES	YES	2	4	1	1	1	1	5	5	4	4
**S.08**	YES	YES	NO	NO	YES	NO	YES	YES	4	4	5	4	1	1	1	2	1	1
**S.09**	YES	YES	NO	NO	NO	NO	YES	YES	2	2	4	4	2	4	4	4	4	4
**S.10**	YES	YES	YES	YES	YES	NO	YES	YES	4	4	2	2	2	2	5	4	2	2
**S.11**	YES	YES	NO	NO	YES	YES	YES	YES	1	1	4	4	1	1	4	2	2	2
**S.12**	YES	YES	YES	YES	NO	NO	YES	YES	1	1	2	4	2	2	5	4	4	4
**S.13**	YES	YES	YES	YES	NO	NO	YES	YES	2	2	4	4	2	2	1	1	4	4
**S.14**	YES	YES	NO	NO	N/A	N/A	YES	YES	5	5	5	5	5	5	4	4	2	2
**S.15**	YES	YES	YES	YES	N/A	N/A	YES	YES	2	2	4	5	1	1	5	5	4	4
**S.16**	YES	YES	NO	NO	N/A	N/A	YES	YES	2	2	2	2	2	4	1	1	2	2
**S.17**	YES	YES	YES	YES	N/A	N/A	YES	YES	2	2	4	4	2	4	5	5	2	2
**S.18**	YES	YES	NO	NO	N/A	N/A	YES	YES	1	1	1	2	1	2	4	4	1	2
**S.19**	YES	YES	YES	YES	N/A	N/A	YES	YES	1	1	5	5	2	4	4	4	2	2
**S.20**	YES	YES	NO	NO	N/A	N/A	YES	YES	4	4	2	5	5	5	4	4	4	4
**S.21**	YES	YES	NO	NO	N/A	N/A	YES	YES	4	5	5	4	1	1	4	4	2	2
**S.22**	YES	YES	NO	NO	N/A	N/A	YES	YES	4	4	2	2	4	5	1	4	1	1
**S.23**	YES	YES	NO	NO	N/A	N/A	YES	YES	4	4	4	4	2	2	4	4	1	1
**S.24**	YES	YES	YES	YES	N/A	N/A	YES	YES	1	1	2	1	1	2	4	2	1	1
**S.25**	YES	YES	NO	NO	N/A	N/A	YES	YES	1	1	1	1	2	4	4	4	2	2
								**Median**	2.0	2.0	4.0	4.0	2.0	2.0	4.0	4.0	2.0	2.0
								**Min.**	1	1	1	1	1	1	1	1	1	1
								**Max.**	5	5	5	5	5	5	5	5	4	4

Table 6. Raters' scores for each of the 25 D/AI solutions

Note: One-scale level gap identified in yellow and two-scale level gap identified in orange.

C. 4. Revision of the 'Human agency' attribute

As we first reached a "moderate agreement" for the 'Human agency' attribute (Table 7), we decided to revise the definition and perform a second interrater agreement assessment. This was aligned with our Phase 3 objective of bringing measurement revision if needed.

Percent agreement					Gwet's AC_2				
N	%	SE	95% CI	*P* value	Coefficient	SE	95% CI	*P* value	Interpretation
25	44%	0.10132	0.231 to 0.649	<.001	0.48998	0.19197	0.094 to 0.886	<.05	Moderate agreement

Table 7. First interrater agreement assessment results for the 'Human agency' attribute

Informed by the diverging interpretations of the two raters, the elements in **red** were deleted and those in **blue** were introduced in the revised and definitive version, while the scale remain unchanged.

Previous version	Definitive version	
**Definition**	**Definition**	**Scale**
Refers to the capacity of individuals and groups to actively and independently decide and act in accordance with their own goals when using a D/AI solution.<br>Though D/AI solutions may improve population health by facilitating a range of human decisions and actions, they may affect user behaviour, cognition, and judgement (e.g., overreliance, overconfidence, hypervigilance) and thus have unexpected impacts on health and social care seeking and provision behaviours.<br>Responsible D/AI solutions can support human agency towards proper health and social care by enabling individuals and groups:<br>• To understand the measures, recommendations, or decisions of a D/AI solution (e.g., data visualization, plain language recommendation, transparency if an AI-based solution is unexplainable);<br>• To discuss with health and social care managers and/or clinical staff the measures, recommendations, or decisions of a D/AI solution (e.g., dedicated point of service);<br>• To act in accordance with their own goals without undue pressure from the D/AI solution itself and from peers (e.g., freedom to use one's judgement or override an AI-based decision, clinical guidelines);<br>• To have their concerns acted upon through a formal committee for audit, review, appeal, and redress mechanism (e.g., ombudsman, committee).	Refers to the capacity of individuals and groups to actively and independently decide and act in accordance with their own goals when using a D/AI solution.<br>Though D/AI solutions may improve population health by facilitating a range of human decisions and actions, they may affect user behaviour, cognition, and judgement (e.g., overreliance, overconfidence, hypervigilance) and thus have unexpected impacts on health and social care seeking and provision behaviours.<br>Responsible D/AI solutions can support human agency towards proper health and social care by actively enabling individuals and groups:<br>• To understand the measures, recommendations, decisions, or outputs of a D/AI solution (e.g., data visualization, plain language recommendation, transparency if an AI-based solution is unexplainable);<br>• To discuss with managers and/or dedicated staff the measures, recommendations, decisions, or outputs of a D/AI solution (e.g., point of service);<br>• To act in accordance with their own goals without undue pressure from the D/AI solution itself and from peers (e.g., freedom to use one's judgement or override an AI-based decision, clinical guidelines);<br>• To have their concerns acted upon through an appeal, audit, review, or redress mechanism (e.g., ombudsman, committee).	The D/AI solution is accompanied by:<br>A. Three of the described enablers or more<br>B. Two of the described enablers<br>C. One of the described enablers<br>D. None of the described enablers

Table 8. Changes brought to the 'Human agency' attribute for the final version of the Tool
